# Stepwise Acetylene Insertion and Ammonia Activation at a Digallene and Diindene

**DOI:** 10.1002/anie.202509661

**Published:** 2025-07-04

**Authors:** Álvaro García‐Romero, Israel Fernández, Jose M. Goicoechea

**Affiliations:** ^1^ Department of Chemistry Indiana University 800 East Kirkwood Ave. Bloomington Indiana 47405 USA; ^2^ Departamento de Química Orgánica and Centro de Innovación en Química Avanzada (ORFEO‐CINQA), Facultad de Ciencias Químicas Universidad Complutense de Madrid Madrid 28040 Spain

**Keywords:** Ammonia activation, Cyclization reactions, Digallenes, Diindenes, Main‐group chemistry

## Abstract

Sequential [2 + 2] cycloaddition reactions between acetylene and the digallene and diindene compounds (ETer)_2_ (E = Ga, In; Ter = 2,6‐Dipp_2_‐C_6_H_3_; Dipp = 2,6‐diisopropylphenyl) are described. Careful control of the reaction conditions leads to selective formation of four‐ and six‐membered rings with 2π E_2_C_2_ and 4π E_2_C_4_ cores, respectively. A structural analysis of the heterocycles by single crystal X‐ray diffraction suggests limited electronic delocalization within the rings, which is borne out in their reactivity. For example, the six‐membered cyclohexadiene analogues exhibit Lewis‐acidic behavior and can form stable, isolable adducts with ammonia. Upon heating, these adducts transform into the corresponding bimetallic triel amides with concomitant generation of ethene.

## Graphical abstract

The stepwise insertion of acetylene into the E═E bonds of digallenes and diindenes is reported. This leads to the selective formation of four‐ and six‐membered rings with 2π E_2_C_2_ and 4π E_2_C_4_ cores. The six‐membered cyclohexadiene analogues are Lewis‐acidic and can form stable, isolable adducts with ammonia, which—upon heating—undergo ammonolysis releasing ethylene.

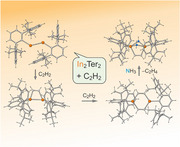

## Introduction

A general trait of transition‐metal complexes, particularly organometallic species, is their ability to stoichiometrically and catalytically activate small molecule substrates such as dihydrogen.^[^ ^1^ ^]^ Over the course of the last twenty years, an increasing body of work has shown that certain compounds of the main‐group elements (typically in low oxidation states) are also capable of such reactivity. Power's landmark report in 2005 that the heavier alkyne analogue (GeTer)_2_ (Ter = 2,6‐Dipp_2_‐C_6_H_3_; Dipp = 2,6‐diisopropylphenyl) was able to activate dihydrogen birthed a renaissance in organometallic main‐group chemistry.^[^ ^2^ ^]^ Since then, significant attention has been paid to main‐group species capable of mimicking the behavior of transition metals.^[^ ^3^ ^]^ Compounds with multiple bonds between the heavier p‐block elements continue to be of relevance in this field and have been shown to activate a number of industrially relevant substrates besides H_2_ including, for example, NH_3_, N_2_O, CO_2_ and C_2_H_4_.^[^ ^4^, ^5^, ^6^, ^7^ ^]^

**Figure 1:**
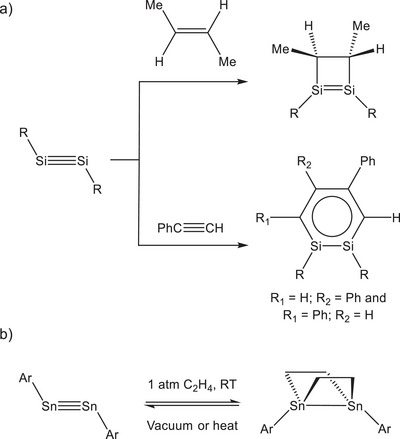
a) Addition of 2‐butene and phenylacetylene to the disilyne RSi≡SiR (R = Si*^i^* Pr[CH(SiMe_3_)_2_]_2_); b) Reversible cycloaddition of ethylene to the distannynes ArSn≡SnAr (Ar = Ter or 2,6‐Tipp_2_‐C_6_H_3_; Tipp = 2,4,6‐triisopropylphenyl).

Reactions involving alkenes and alkynes are particularly interesting due to their enormous industrial relevance. Transiently generated compounds with E═E double bonds (E = Si, Ge) have been known to undergo [2 + 2] cycloaddition reactions with alkenes since the early 1990s.^[^ ^8^, ^9^, ^10^ ^]^ Sekiguchi's isolation of an isolable compound with a Si≡Si triple bond in 2004 allowed for a more methodical study of such transformations.^[^ ^11^ ^]^ Cycloaddition reactions of disilynes, such as RSi≡SiR (R = Si*^i^* Pr[CH(SiMe_3_)_2_]_2_), with alkenes are known to stereo‐specifically afford four‐membered 1,2‐disilacyclobutenes (Figure 1a).^[^ ^12^, ^13^ ^]^ By contrast, RSi≡SiR reacts with phenylacetylene to afford 1,2‐disilabenzenes with aromatic Si_2_C_4_ rings.^[^ ^13^ ^]^ In 2009, Power and co‐workers described the reversible cycloaddition of ethene with the distannyne (SnAr)_2_ (Ar = Ter or 2,6‐Tipp_2_‐C_6_H_3_; Tipp = 2,4,6‐triisopropylphenyl). Regeneration of the distannyne, with concomitant release of ethylene, can be accomplished by applying reduced pressure or heating (Figure 1b).^[^ ^14^ ^]^ Since then, numerous examples of alkene and alkyne activation reactions by group 14 compounds have been reported including compounds with E═E double bonds,^[^ ^15^ ^]^ E≡E triple bonds,^[^ ^16^, ^17^ ^]^ and by monomeric carbene analogues.^[^ ^18^, ^19^, ^20^, ^21^ ^]^

**Figure 2:**
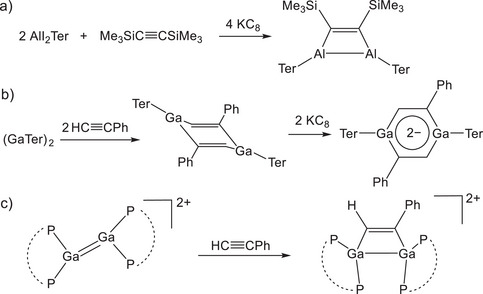
Reported cyclization reactions involving heavier group 13 alkene analogues to afford: a) an Al_2_C_2_ cyclobutene, b) a Ga_2_C_4_ cyclohexadiene and digallatabenzene, and c) a Ga_2_C_2_ dicationic digallacyclobutene.

Cyclization reactions between compounds of the group 13 elements and alkenes or alkynes are far less common.^[^ ^22^, ^23^ ^]^ In 2006, Cui and co‐workers reported the synthesis of a 1,2‐dialuminacyclobutene with a 2π Al_2_C_2_ core, which was generated by the in‐situ reduction of AlI_2_Ter with KC_8_ in the presence of Me_3_SiC≡CSiMe_3_ (Figure 2a).^[^ ^24^ ^]^ In 2017, Inoue described the isolation of the first stable diaalumene and showed that it could also undergo [2 + 2] cycloaddition reactions with phenylacetylene.^[^ ^25^ ^]^ For gallium‐containing compounds, Power was the first to show that the digallene (GaTer)_2_ reacts with phenylacetylene to produce the digallacyclohexadiene (GaTer)_2_(HCCPh)_2_. This compound can be readily reduced to afford the digallatabenzene K_2_[(GaTer)_2_(HCCPh)_2_] (Figure 2b).^[^ ^26^ ^]^ Most recently, Krossing and co‐workers have explored [2 + 2] cycloaddition reactions of alkynes with dicationic digallenes.^[^ ^27^, ^28^, ^29^ ^]^ For instance, the reaction of phenylacetylene with the digallene [Ga(dipf)]_2_^2+^ (dipf = 1,1′‐bis(diisopropylphosphino)ferrocene) leads to the formation of the digallacyclobutene [Ga_2_(dipf)_2_(HCCPh)]^2+^ containing a 2π Ga_2_C_2_ core (Figure 2c). Addition of excess phenylacetylene does not lead to the formation of a digallacyclohexadiene.^[^ ^28^ ^]^ During the preparation of this manuscript, Cowley reported on the reversible addition of ethene to digallenes.^[^ ^30^ ^]^ In addition to these transformations, heavier group 13 alkene analogues have shown outstanding potential for cycloaddition reactions of polyolefins.^[^ ^31^ ^]^ To the best of our knowledge, no [2 + 2] cycloadditions of alkenes or alkynes to diindenes have been described, despite extensive efforts. For example, Krossing has described reactions of [In(dipf)]_2_^2+^ with such substrates as “futile”, giving rise to indium(0) precipitates.^[^ ^27^ ^]^

The examples described above illustrate the ability of heavier alkene analogues to undergo single and double [2 + 2] cycloaddition reactions with C≡C triple bonds, however, the factors which govern the formation of four‐ or six‐membered ring systems remain poorly understood. Inspired by these studies, we decided to explore these reactivity patterns more closely, with a particular focus on accessing the elusive indium derivatives. Herein, we describe the formation of unsubstituted cyclic compounds with 2π E_2_C_2_ and 4π E_2_C_4_ (E = Ga, In) cores. This can be accomplished by the treatment of (GaTer)_2_ and (InTer)_2_^[^ ^32^, ^33^ ^]^ with acetylene. We further explored the Lewis acidic behavior of the E_2_C_4_ (E = In, Ga) rings by their reactions with ammonia, which afforded simple adducts that, upon heating, undergo ammonolysis to produce ethylene and the corresponding bimetallic gallium and indium amides.

## Results and Discussion

### Acetylene Insertion

**Scheme 1:**
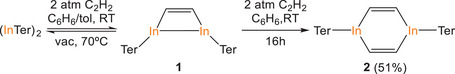
Synthesis of (InTer)_2_(C_2_H_2_) (**1**) and (InTer)_2_(C_2_H_2_)_2_ (**2**).

Power's synthesis of a gallium‐containing cyclohexadiene^[^ ^26^ ^]^ inspired us to attempt the synthesis of an indium analogue. Treatment of a solution of (InTer)_2_ in benzene with 2 atm of acetylene at room temperature results in a color change of the reaction mixture from deep red to yellow. Immediate removal of the volatiles and recrystallization from hexane at −35 °C resulted in the formation of a mixture of deep red and yellow crystals, both of which were analyzed by single crystal X‐ray diffraction. The deep red crystals correspond to the starting material (InTer)_2_.^[^ ^33^ ^]^ The yellow crystals were identified as compound (InTer)_2_(C_2_H_2_) (**1**), which features a four‐membered In_2_C_2_ ring resulting from the [2 + 2] cycloaddition of acetylene with the In═In bond of the precursor (Scheme 1). Formally, this reaction represents an oxidation of the indium(I) centers in (InTer)_2_ to indium(II).

By contrast, prolonged treatment of (InTer)_2_ with 2 atm of acetylene results in the formation of compound (InTer)_2_(C_2_H_2_)_2_ (**2**), containing a six‐membered In_2_C_4_ ring arising from the insertion of two acetylene molecules in the In═In bond (see structural discussion below). To gain further insight into the formation of **1** and **2**, a solution of (InTer)_2_ was monitored by ^1^H NMR spectroscopy after treatment with 2 atm of acetylene. After 5 min, the ^1^H NMR spectrum reveals quantitative formation of **1**, which shows a characteristic singlet resonance at 9.71 ppm corresponding to the alkenyl protons. Over time, the ^1^H NMR spectra revealed gradual consumption of **1** and the formation of a new product, **2**. The reaction was complete after a period of 16 h at room temperature (Figure S44). The ^1^H NMR spectrum of **2** displays a singlet at 6.85 ppm corresponding to the alkenyl protons, the integral of which is consistent with a 1:2 (InTer)_2_/C_2_H_2_ reaction (see SI).

Interestingly, attempts to isolate **1** as a compositionally pure solid resulted in mixtures of both **1** and (InTer)_2_. Reaction mixtures can be monitored by NMR spectroscopy until there is evidence for quantitative formation of **1**; however, removal of the volatiles invariably gives rise to mixtures of the targeted product and (InTer)_2_. This suggests that the [2 + 2] cycloaddition reaction is reversible. Fine‐tuning of the conditions allows for quantitative retro‐cyclization of **1** to yield (InTer)_2_ and free acetylene under reduced pressure and vigorous stirring of a toluene solution at 70 °C (Figure S45). To the best of our knowledge, the only known example of reversible acetylene activation by a compound of the group 13 elements was described by Fedushkin in 2010.^[^ ^34^ ^]^ However, in this case, the reactivity was facilitated by redox noninnocent ligands and was not associated with the Ga─Ga bond.

**Figure 3:**
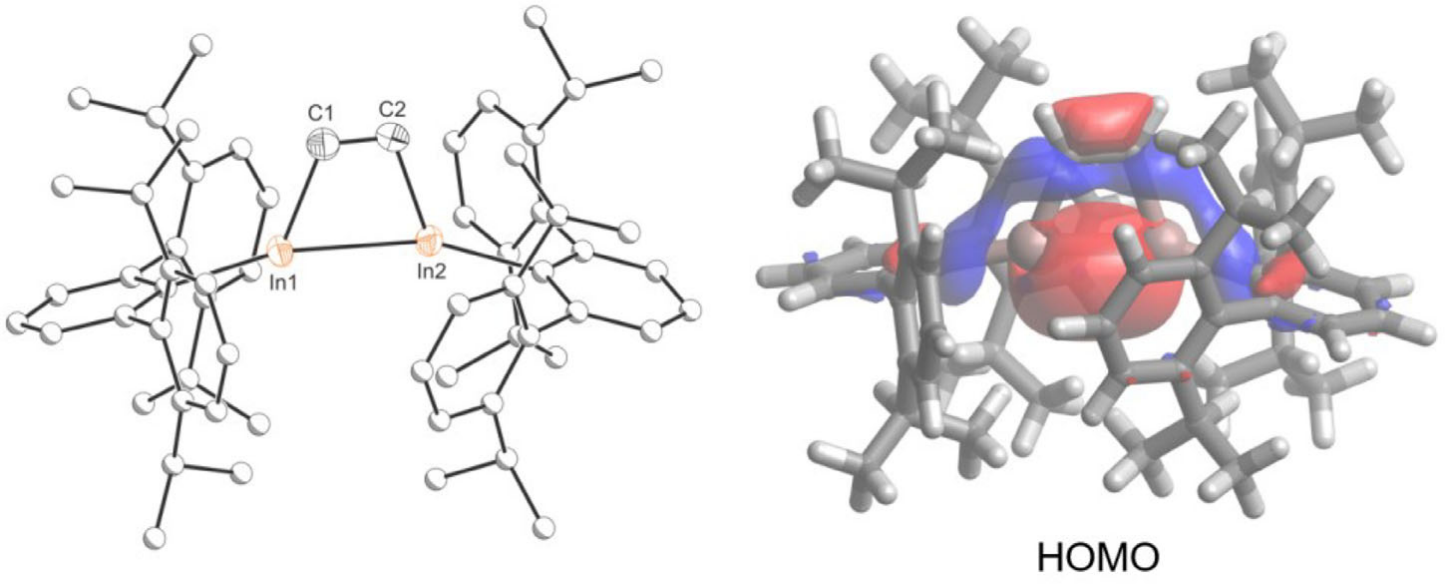
Single crystal X‐ray structure (left) and HOMO (right, 0.04 isovalue) of **1**. Thermal ellipsoids set at 50% probability level; hydrogen atoms omitted for clarity. Carbon atoms of Ter are depicted as spheres of arbitrary radius.

The solid‐state structure of **1** (Figure 3, left) shows the presence of a twisted four‐membered In_2_C_2_ ring.^[^ ^35^ ^]^ The trapezoidal‐like In_2_C_2_ core reveals obtuse In‐C‐C angles (109.22(16) and 109.56(16)°) and acute C‐In‐In angles (70.26(6) and 70.65(6)°), consistent with the mismatch in covalent radii between carbon and indium. The inter‐ring C–C distance (1.325(3) Å) suggests significant double bond character [Σ_cov_(C═C) = 1.34 Å] while the In–C bond lengths (2.1808(19) and 2.199(2) Å) are in line with single bonds [Σ_cov_(In─C) = 2.17 Å].^[^ ^36^, ^37^ ^]^ The In─In distance (2.7906(2) Å) is slightly shorter than expected for an In─In single bond (Σ_cov_(In─In) = 2.84 Å)^[^ ^37^ ^]^ but comparable to other previously reported In^II^–In^II^ single bonds (e. g. (Tipp)_2_In─In(Tipp)_2_, 2.775(2) Å; Tipp = 2,4,6‐triisopropylphenyl).^[^ ^38^ ^]^ To better understand the electronic structure of **1**, density functional theory (DFT) calculations at the dispersion corrected CPCM‐(RI)‐PBE0‐D3(BJ)/def2‐SVP level were performed (see the Supporting Information for full computational details). The highest occupied molecular orbital (HOMO) primarily reflects the σ‐bonding interaction between the indium(II) centers (Figure 3, right). The calculated Mayer bond order for the In─In bond is 0.74, which is consistent with an In─In single bond.

To evaluate the stability of **1** in solution, excess acetylene was immediately removed after the reaction had reached completion through six freeze‐pump‐thaw cycles. In the absence of acetylene, compound (In^II^Ter)_2_(C_2_H_2_) (**1**) readily disproportionates yielding (In^I^Ter)_2_ and (In^III^Ter)_2_(C_2_H_2_)_2_ (**2**) over a period of ∼1 week at room temperature (Figure S46).

**Figure 4:**
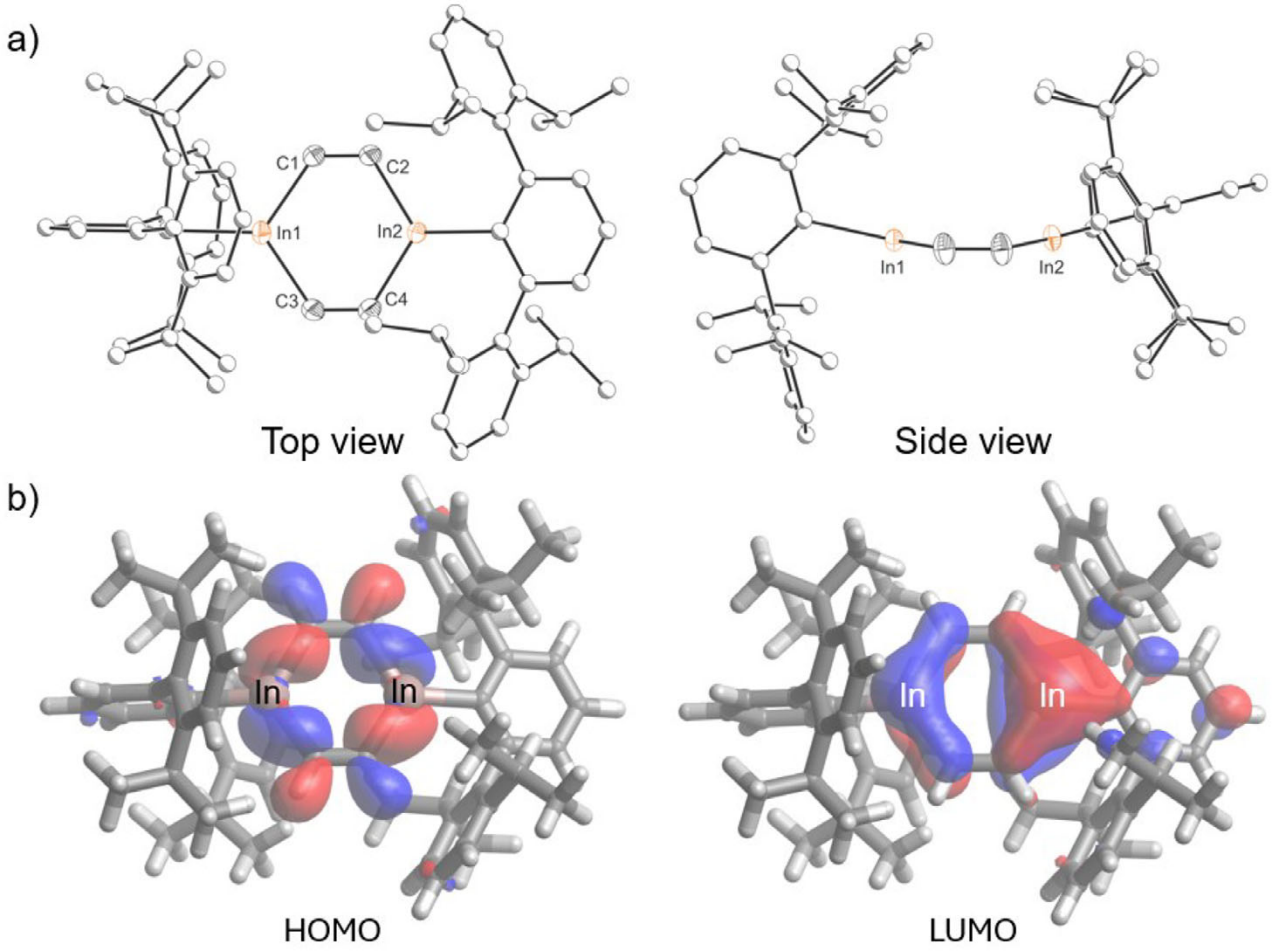
a) Single crystal X‐ray structure; and b) frontier orbitals (0.04 isovalue) of **2**. Thermal ellipsoids set at 50% probability level; hydrogen atoms omitted for clarity. Carbon atoms of Ter are depicted as spheres of arbitrary radius.

As mentioned above, the reaction of (InTer)_2_ with 2 atm of acetylene for 16 h affords **2** as a compositionally pure yellow solid in 51% yield (Scheme 1). Compound **2** remains intact even under vacuum at 70 °C in toluene solution, indicating the irreversible nature of this reaction. Yellow crystals of **2** suitable for X‐ray diffraction were obtained from a concentrated hexane solution at −35 °C. The 4π In_2_C_4_ ring in **2** adopts a boat‐like conformation (Figure 4a). The fold angles between the C1‐C2‐C3‐C4 plane and the C1‐In1‐C3 and C2‐In2‐C4 planes are 9.3 and 13.1°, respectively. The distorted hexagonal‐like ring In_2_C_4_ ring reveals In‐C‐C angles in the range of 122.5(2)–124.4(2)°, and C‐In‐C angles of 111.52(11) and 111.78(11)°. The inter‐ring C═C bonds (1.335(4) and 1.341(4) Å; cf. Σ_cov_(C═C) = 1.34 Å) and In─C bonds (2.144(3)–2.153(3) Å, cf. Σ_cov_(In─C) = 2.17) are as expected for localized C═C and In─C bonds. In contrast to **1**, **2** does not exhibit an In─In bond (*d*_In─In_ = 3.703(1); cf. Σ_cov_(In─In) = 2.84 Å). One of the central rings of the terphenyl groups bonded to the indium centers is nearly co‐planar to the central In_2_C_4_ ring (14.4°), while the other one is perpendicular (92.8°). DFT calculations were used to interrogate the nature of the highest occupied molecular orbital (HOMO) and the lowest unoccupied molecular orbital (LUMO) for **2** (Figure 4b). The HOMO is predominantly composed of In─C σ‐bonding interactions in the central ring, with some participation from the C─H bonds, whereas the LUMO has significant indium p‐orbital character (ca. 40%), suggesting that the compound has electrophilic character.

**Figure 5:**
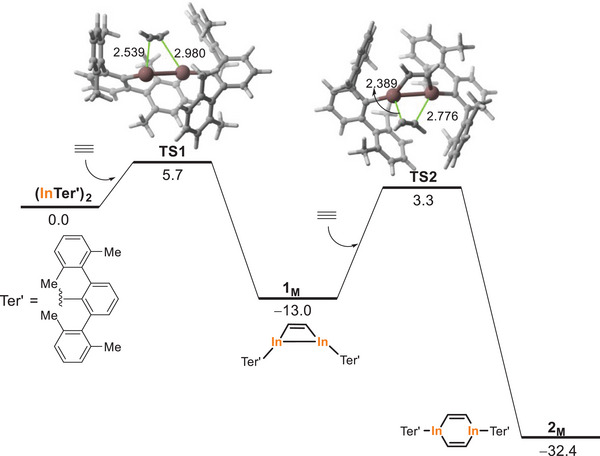
Computed reaction profile for the formation of **1**_**M**_ and **2**_**M**_. Relative free energies (Δ*G*, at 298 K) and bond distances are given in kcal mol^−1^ and angstroms, respectively. All data have been computed at the CPCM‐(RI)‐PBE0‐D3(BJ)/def2‐SVP level.

Additional DFT calculations were carried out to support the reversible formation of **1** and the irreversible nature of the reaction leading to **2**. Figure 5 shows the computed reaction profile for the formation of these species starting from (InTer')_2_, a model system of (InTer)_2_ where the bulky isopropyl substituents in the Ter groups were replaced by methyl groups. In both cases, it is found that the corresponding [2 + 2] cycloaddition reactions occur in a concerted manner through the corresponding four‐membered transition states **TS1** and **TS2**, respectively, leading to the exergonic formation of **1**_**M**_ and **2_M_** (Δ*G*_R_ = −13.0 and −19.4 kcal mol^−1^, respectively). Interestingly, the free activation barrier required for the formation of **1_M_** is rather low (Δ*G* ^≠^ = 5.7 kcal mol^−1^), which is not only compatible with a process occurring at room temperature but also confirms the reversible nature of this process (the barrier for the retro‐cyclization is only ΔG^≠^ = 18.7 kcal mol^−1^). At variance, the barrier for the formation of **2_M_** is much higher (Δ*G* ^≠^ = 16.3 kcal mol^−1^) which, together with the high exergonicity of the process, makes the possible retro‐cyclization unfeasible (Δ*G* ^≠^ = 35.7 kcal mol^−1^), therefore supporting the irreversible nature of this transformation.

**Scheme 2:**
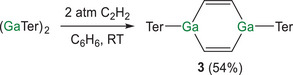
Synthesis of (GaTer)_2_(C_2_H_2_) (**3**).

Having shown that the reaction between acetylene and (InTer)_2_ results in the formation of both four‐ and six‐membered rings, we turned our attention to the reactivity of (GaTer)_2_. Specifically, we were intrigued to explore whether a cyclic four‐membered ring could be synthesized. Treatment of a benzene solution of (GaTer)_2_ with 2 atm of acetylene at room temperature results in immediate discoloration of the reaction mixture (Scheme 2). In this case, the ^1^H NMR spectrum is consistent with the formation of (GaTer)_2_(C_2_H_2_)_2_ (**3**), revealing a singlet resonance at 6.69 ppm corresponding to the alkenyl protons. Integration of this resonance against those arising from the Ter substituents suggests a 1:2 (GaTer)_2_/C_2_H_2_ stoichiometry (see Supporting Information).

**Figure 6:**
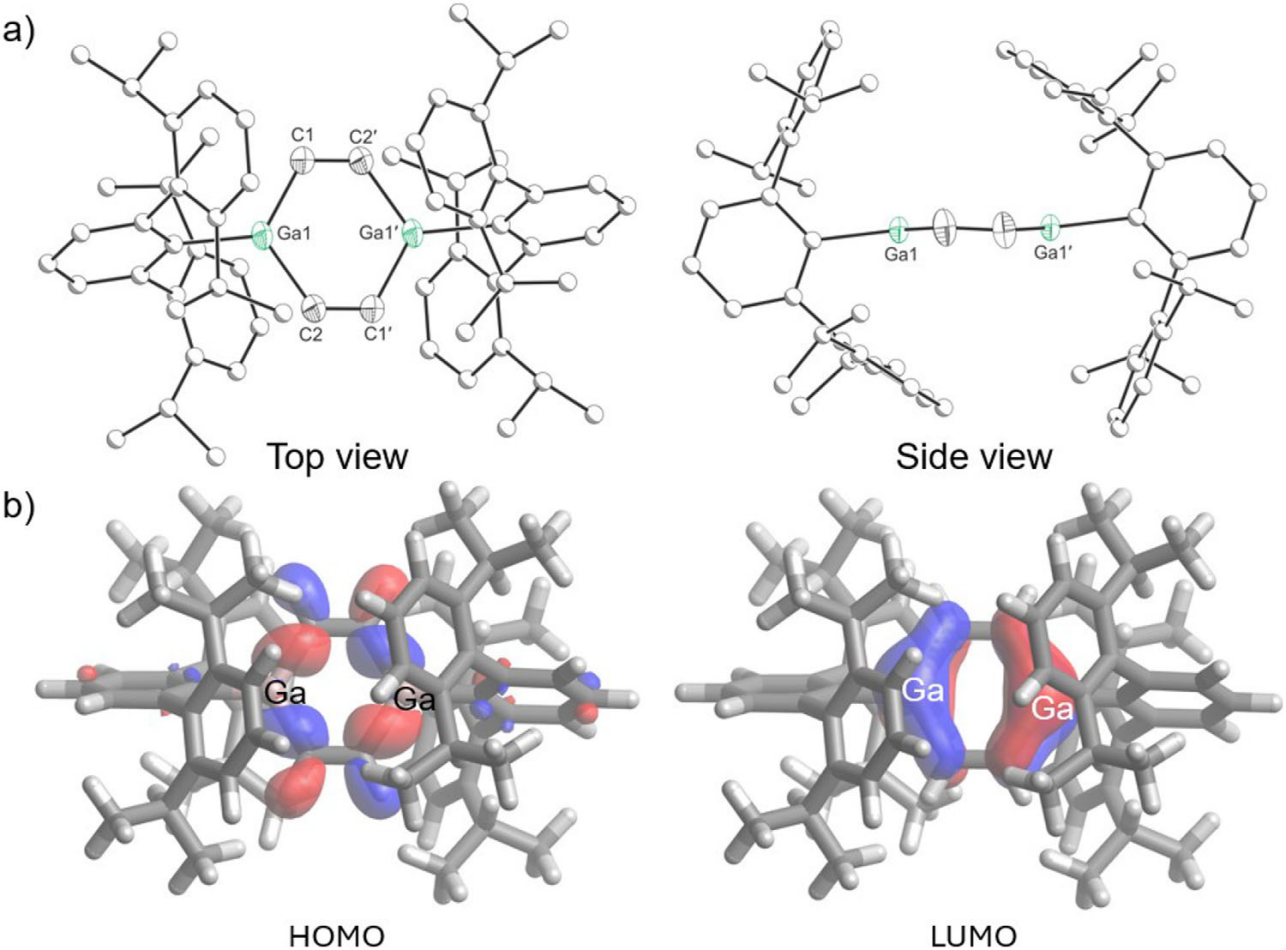
a) Single crystal X‐ray structure; b) and frontier orbitals (bottom, 0.04 isovalue) of **3**. Thermal ellipsoids set at 50% probability level; hydrogen atoms omitted for clarity. Carbon atoms of Ter are depicted as spheres of arbitrary radius.

Compound **3** was isolated in 54% yield as a colorless powder. Crystals suitable for X‐ray diffraction were obtained by slow evaporation of a concentrated hexane solution at −35 °C. The solid‐state structure of **3** shows two independent molecules in the asymmetric unit, both of which feature nearly planar Ga_2_C_4_ six‐membered rings (mean deviations from plane: 0.02 and 0.04 Å; Figure 6a). This planarity contrasts with the previously reported Ga_2_C_4_ ring in (GaTer)_2_(HCCPh)_2_, which has a flattened‐chair conformation.^[^ ^26^ ^]^ The Ga_2_C_4_ rings reveal C−C (1.343(3), 1.351(3) Å) and Ga–C bond lengths (1.935(3)–1.957(2) Å), which are in good agreement with (GaTer)_2_(HCCPh)_2_ [*d*_C═C_ = 1.353(2), *d*_Ga─C _= 1.959(2) and 1.967(2) Å]. DFT calculations reveal that the frontier molecular orbitals of **3** are comparable to those of **2** (Figure 6b).

**Scheme 3:**
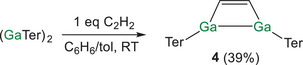
Synthesis of (GaTer)_2_(C_2_H_2_) (**4**).

The enhanced reactivity of (GaTer)_2_ toward acetylene can be attributed to the increased stability of the +3 oxidation state for gallium relative to indium.^[^ ^39^ ^]^ In light of this result, we questioned whether the hypothetical gallium analogue of **1**, (GaTer)_2_(C_2_H_2_) could be obtained. This prompted us to explore the stoichiometric reaction of (GaTer)_2_ with acetylene. Addition of 1 equivalent of acetylene to (GaTer)_2_ results in the quantitative conversion to (GaTer)_2_(C_2_H_2_) (**3**) as evidenced by ^1^H NMR spectroscopy (Scheme 3). The ^1^H NMR spectrum of **3** reveals a singlet resonance at 9.36 ppm, corresponding to the alkenyl protons (see SI). In contrast to **1**, compound **4** is stable in benzene solution at room temperature and no reversibility or disproportion to **3** and (GaTer)_2_ was observed by ^1^H NMR over a period of 3 days.

**Figure 7:**
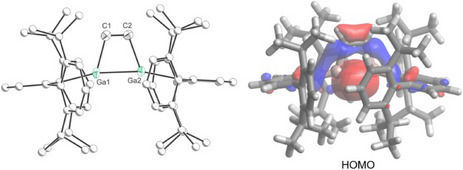
Single crystal X‐ray structure (left) and HOMO (right, 0.04 isovalue) of **4**. Thermal ellipsoids set at 50% probability level; hydrogen atoms omitted for clarity. Carbon atoms of Ter are depicted as spheres of arbitrary radius.

Compound **4** was isolated in 39% yield as colorless crystals. The solid‐state structure of **4** reveals a 2π Ga_2_C_2_ four‐membered ring (Figure 7, left). The trapezoidal‐like Ga_2_C_2_ core reveals obtuse C‐C‐Ga angles (105.8(2) and 106.6(2)°) and acute C‐Ga‐Ga angles (72.74(10) and 73.17(9)°). The C–C distance (1.332(5) Å) is consistent with the presence of a localized double bond while the Ga–C bond lengths (both 1.990(3) Å) are in line with the value expected for a single bond [Σ_cov_(Ga─C) = 1.99 Å].^[^ ^35^ ^]^ The Ga–Ga distance (2.4787(4) Å) suggests the presence of a bond (Σ_cov_(Ga─Ga) = 2.48 Å) and is comparable with other previously reported Ga^II^─Ga^II^ single bonds (e.g., ITerGa─GaTerI, *d*_Ga─Ga_ = 2.492(2) Å).^[^ ^32^ ^]^ The computed HOMO primarily reflects the σ‐bonding interaction between the two gallium centers (Figure 7, right). Additionally, the calculated Mayer bond order for the Ga─Ga bond is 0.79, again corroborating the presence of a single bond.

These results demonstrate how, under the appropriate conditions, the reaction of (InTer)_2_ and (GaTer)_2_ with acetylene can lead to the selective formation of 2π E_2_C_2_ or 4π E_2_C_4_ rings (E = In, Ga). While the reaction of (In‐Ter)_2_(C_2_H_2_) (**1**) with acetylene to form (InTer)_2_(C_2_H_2_)_2_ (**2**) requires several hours, the reaction of (GaTer)_2_(C_2_H_2_) (**4**) is significantly faster. This makes the isolation of **4** more challenging, requiring precise stoichiometric control of the added acetylene.

### Ammonia Coordination

While transition metals are known to readily form coordination complexes with ammonia, organometallic compounds of the heavier main group elements are less prone to form such adducts. This is because the E─C bonds in such species can readily undergo uncontrolled ammonolysis reactions. Careful tuning of ligand steric and electronic properties allows for more controlled reactivity. In the context of the group 13 elements, the controlled activation of ammonia has been shown to be viable in a handful of cases.^[^ ^40^, ^41^, ^42^, ^43^ ^]^ This reactivity makes the isolation of ammonia adducts of such elements challenging. Examples of structurally authenticated ammonia complexes of gallium are rare,^[^ ^44^, ^45^, ^46^ ^]^ while those of indium are rarer still.^[^ ^47^ ^]^

**Scheme 4:**
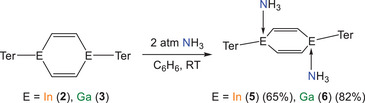
Synthesis of (InTer)_2_(C_2_H_2_)_2_(NH_3_)_2_ (**5**) and (GaTer)_2_(C_2_H_2_)_2_(NH_3_)_2_ (**6**).

Our DFT calculations revealed that compounds **2** and **3** have energetically accessible LUMOs, and that these are predominantly composed of triel element p orbitals. This prompted us to explore their Lewis acidity. The treatment of benzene solutions of **2** or **3** with 2 atm of NH_3_ gives rise to the formation of **5** and **6** in 65 and 82% yields, respectively (Scheme 4). Compounds **5** and **6** show a characteristic singlet resonance at 0.18 and 0.31 ppm, respectively, corresponding to the NH_3_ moieties (see SI). Furthermore, the ^1^H–^1^H NOESY NMR spectra of **5** and **6** reveal interactions between the NH_3_ protons and those of the Ter ligands, indicating that they are proximal. These results are strongly indicative of adduct formation between **2**/**3** and ammonia in solution (Figures S23 and S32).

**Figure 8:**
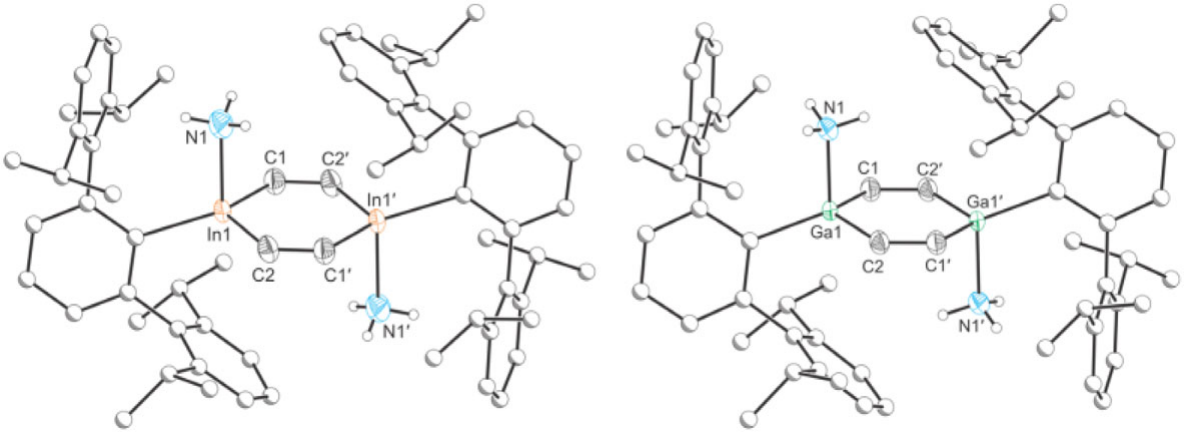
Single crystal X‐ray structures of **5** (left) and **6** (right). Thermal ellipsoids set at 50% probability level; hydrogen atoms (except those of NH_3_) and solvent of crystallization are omitted for clarity. Carbon atoms of Ter are depicted as spheres of arbitrary radius.

The solid‐state structure of **5** confirms coordination of an NH_3_ molecule to each of the indium centers (Figure 8, left). The In_2_C_4_ ring adopts a nearly planar conformation with a distorted hexagonal structure (In‐C‐C angles: 122.50(15) and 123.09(15)°; C‐In‐C angle 114.27(7)°). The In─N bond (2.357(2) Å) is slightly longer than the sum of the covalent radii (Σ_cov_(In─N) = 2.13 Å), and perpendicular to the planar ring (N‐In‐C angles: 90.06(9) and 95.11(9)°). The inter‐ring bond distances are comparable to those of **2** (C═C bond: 1.340(3) Å; and In─C bonds 2.160(2) and 2.162(2) Å). The FTIR spectrum of **5** displays two bands at 3278 and 3368 cm^−1^ due to the N─H stretching modes of the NH_3_ groups (Figure S55), in good agreement with previously reported ammonia complexes of gallium.^[^ ^45^ ^]^ The solid‐state structure of **6** is similar to that of **5** (Figure 8, right). Worth noting is the Ga–N distance (2.115 (1) Å), which again is slightly longer than expected for a Ga─N single bond (Σ_cov_ (Ga─N) = 1.99 Å) but in good agreement with previously reported Ga─NH_3_ adducts (e.g., Ga─N 2.082(5) Å). In this case, the FTIR spectrum also shows the corresponding bands for the N─H stretching modes at 3267 and 3357 cm^−1^ (Figure S57).

### Intramolecular Ammonolysis

**Scheme 5:**
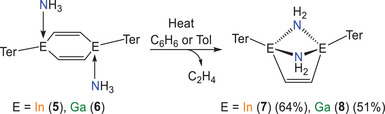
Synthesis of (InTer)_2_(C_2_H_2_)(μ‐NH_2_)_2_ (**7**) and (GaTer)_2_(C_2_H_2_)(μ‐NH_2_)_2_ (**8**).

**Figure 9:**
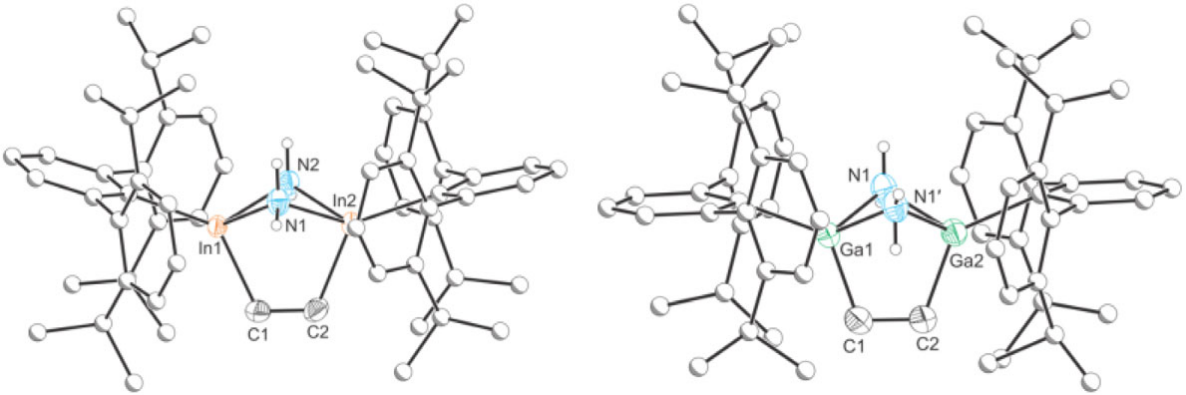
Single crystal X‐ray structure of **7** (left) and **8** (right). Thermal ellipsoids set at 50% probability level; hydrogen atoms (except those of NH_2_ groups) omitted for clarity. Carbon atoms of Ter are depicted as spheres of arbitrary radius.

The coordination of ammonia to Lewis acidic metals is known to decrease its N─H bond dissociation energy, enabling both hydrogen atom abstraction and deprotonation reactions.^[^ ^48^, ^49^ ^]^ Adduct formation is also known to play an important role in the formal oxidative‐addition of ammonia at low oxidation‐state main group compounds, although examples of structurally authenticated adducts that subsequently undergo bond activation reactions are rare.^[^ ^41^, ^44^, ^50^, ^51^, ^52^, ^53^ ^]^ In view of the well‐defined solid‐state structures of **5** and **6**, in which alkenyl and ammonia groups are bonded to the same triel atom, we were intrigued to see whether we could induce N─H bond cleavage in such compounds. Heating a solution of **5** at 80 °C for a period of 6 h, results in the quantitative formation of the indium diamide (InTer)_2_(C_2_H_2_)(μ‐NH_2_)_2_ (**7**) with concomitant evolution of ethylene (Scheme 5). In situ ^1^H NMR spectroscopy confirms the generation of ethylene (singlet at 5.25 ppm) and the presence of the characteristic doublet resonances at −0.96 and −1.86 ppm (^2^ *J*_H─H_ = 9.6 Hz) corresponding to the NH_2_ protons of the amide fragments in **7** (Figure 9, left). It is worth noting that the formal oxidation state of indium in both reagents and products remains constant.

Compound **7** was isolated in 64% yield as a colorless powder. Crystals suitable for X‐ray diffraction were grown from a concentrated hexane solution at −35 °C. The X‐ray structure of **7** reveals two indium centres bridged by a C_2_H_2_ and two NH_2_ moieties (Figure 9, left). The In–N distances range between 2.205(4) and 2.216(4) Å and are similar to previously reported dimeric indium amides (e.g [Me_2_InN(H)CH_2_Ph]_2_ *d*_In─N_ = 2.205(9)–2.234(6)).^[^ ^54^ ^]^ The C═C double bond and In─C single bond distances (1.337(9); 2.157(5) and 2.165(6) Å, respectively) in **7** are comparable to those found in the parent compound **5**. FTIR studies revealed two weak bands at 3309 and 3380 cm^−1^ due to the N─H stretching modes of the NH_2_ moieties (Figure S58), similar to those observed for the tin amide {SnTer(μ‐NH_2_)}_2_.^[^ ^50^ ^]^ The unusual morphology of **7** is analogous to bicyclo[2.1.1]hex‐2‐ene.^[^ ^55^ ^]^

The synthesis of (GaTer)_2_(C_2_H_2_)(μ‐NH_2_)_2_ (**8**) requires much more energetic conditions than its indium‐containing analogue. A toluene solution of **6** required heating at 110 °C for 8 days to reach full conversion as evidenced by ^1^H NMR spectroscopy (Scheme 5). In this case, the formation of ethylene and the appearance of two doublet resonances at −0.57 and −1.54 ppm was also observed in the final ^1^H NMR spectrum (see ESI). Colorless crystals suitable for X‐ray diffraction were grown from a concentrated hexane solution of **8** at −35 °C. Despite the existence of positional disorder, the structure was found to be very similar to that of **7** (Figure 9, right). The Ga–N distances range between 1.9965(16) and 2.0366(17) Å and are similar to previously reported dimeric gallium amides (e.g [GaAr(μ‐NH_2_)H]_2_ (Ar = 2,6‐(2,6‐*^i^* Pr_2_C_6_H_3_‐4‐(Me_3_Si)C_6_H_2_), *d*_Ga–N_ = 1.986(2)–1.988(2)).^[^ ^41^ ^]^ In the FTIR spectrum, two weak bands arising from the N─H stretching vibrations can be found at 3313 and 3383 cm^−1^ (Figure S46).

In order to obtain further insight into the ammonolysis reaction of **5**, the deuterated analogue (InTer)_2_(C_2_H_2_)_2_(ND_3_)_2_ (**5_D_**) was prepared in 62% yield by the reaction of **2** with 1 atm of ND_3_. Compound **5_D_** shows an identical ^1^H NMR spectrum to that of **5** with the notable absence of a resonance for NH_3_ (Figure S25). Instead, the ^2^H{^1^H} NMR spectrum shows a singlet resonance at 0.10 ppm, arising from the ND_3_ molecules (Figure S26). The FTIR spectrum displays two bands at 2511 and 2376 cm^−1^ due to the N─D stretching modes (Figure S56). Thermolysis of **5_D_** requires more elevated temperatures than its nondeuterated analogue **5** due to the primary kinetic isotope effect.^[^ ^56^ ^]^ Heating a solution of **5_D_** at 110 °C in toluene for a period of 12 h (or at 80 °C for 30 h in benzene solution), results in the formation of the indium diamide (InTer)_2_(C_2_H_2_)(μ‐ND_2_)_2_ (**7_D_**) accompanied by the release of *d*_2_‐ethene as evidenced by NMR spectroscopy. In situ ^2^H{^1^H} NMR experiments confirm the generation of deuterated ethene (observed as a singlet at 5.26 ppm, Figure S51). The presence of residual protons in the ammonia of **5_D_** (≈6%) leads to the formation of several ethene isotopologues as revealed by ^1^H NMR spectroscopy, C_2_D_2_H_2_, C_2_DH_3_ and C_2_H_4_ (Figure S50). The low concentration in the solution phase, along with signal overlap and the complex splitting of the ethene signals (*J*_H–D_ < 2 Hz) preclude further quantitative analysis.

**Figure 10:**
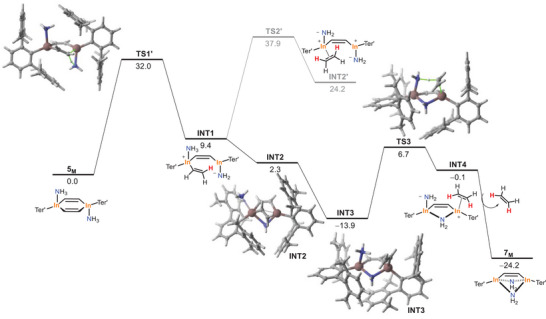
Computed reaction profile for the formation of **7_M_** from **5_M_**. Relative free energies (Δ*G*, at 298 K) are given in kcal mol^−1^. All data have been computed at the CPCM‐(RI)‐PBE0‐D3(BJ)/def2‐SVP level.

In addition to the labelling experiments discussed above, DFT calculations were also carried out to explore the mechanism involved in the ammonolysis reaction. To this end, the transformation of **5_M_**, a model of compound **5** where again the isopropyl substituents were replaced by methyl groups, to **7_M_** and ethene was studied. As shown in Figure 10, species **5_M_** is transformed into intermediate **INT1** through **TS1'**, a saddle point associated with the proton migration from the coordinated NH_3_ to the adjacent carbon atom with concomitant rupture of the In─C bond. This endergonic step (Δ*G*_R_ = 9.4 kcal mol^−1^) exhibits an activation barrier of 32.0 kcal mol^−1^, which is, therefore, consistent with the temperature required in the experiments (80 °C). From **INT1**, a similar proton migration from the other In─NH_3_ moiety can be envisaged. However, our calculations indicate that this process (involving **TS2'**) is very unlikely in view of the high barrier and endergonicity computed for this step. Alternatively, we found that **INT1** can directly evolve into **INT3** (via **INT2**) in an essentially barrierless process (due to the highly exergonicity of the process, Δ*G*_R_ = −23.3 kcal mol^−1^) where the readily formed, highly nucleophilic NH_2_^─^ fragment binds the other indium center. From **INT3**, an analogous proton migration from the In─NH_3_ group to the adjacent carbon atom produces **INT4** via **TS3**, with a feasible activation barrier of 20.6 kcal mol^−1^. Final exergonic ethene displacement by the NH_2_^–^ ligand produces the observed bimetallic amide‐bridged species **7_M_** with concomitant release of a molecule of ethene. Our calculations therefore suggest that the initial In─C protonolysis becomes the rate‐determining step of the transformation, which is consistent with the labelling experiments discussed previously.

## Conclusions

We have shown that sequential [2 + 2] cycloaddition reactions are possible between the heavier alkene analogues (ETer)_2_ (E = Ga, In) and acetylene. These stepwise insertion reactions afford cyclic compounds with electronically interesting 2π E_2_C_2_ and 4π E_2_C_4_ cores. All these cyclic compounds display bond metric data and reactivity profiles that suggest localized C═C double and E─C single bonds. This can be probed experimentally by reaction of the cyclohexadiene analogues (ETer)_2_(C_2_H_2_)_2_ (E = In (**2**), Ga (**3**)) with ammonia which affords stable adducts in both solution and the solid state. Adduct formation serves to weaken the N─H bonds of the coordinated ammonia molecules as thermal treatment of the adducts (**5** and **6**) results in the loss of ethylene and the formation of bimetallic amide‐bridged dimers.

## Experimental Section

### General synthetic methods

All reactions and product manipulations were carried out using standard Schlenk‐line techniques under an inert atmosphere of argon, or in a dinitrogen filled glovebox (MBraun UNIlab glovebox maintained at <0.1 ppm H_2_O and <0.1 ppm O_2_). (GaTer)_2_^[^ ^32^ ^]^ and (InTer)_2_^[^ ^33^ ^]^ were synthesized according to previously reported synthetic procedures. Benzene (anhydrous, Sigma Aldrich), toluene (Fisher Chemical, HPLC grade), hexane (Fisher Chemical, HPLC grade), and pentane (Fisher Chemical, HPLC grade) were purified using a pure process technology (PPT) solvent purification system (SPS). C_6_D_6_ (Aldrich, 99.5%) and *d*_8_‐toluene (Aldrich, 99%) were distilled over sodium/benzophenone. All dry solvents were stored under argon in gas‐tight ampoules over activated 3 Å molecular sieves.

### Characterization techniques

NMR spectra were acquired on a Bruker 500 MHz Avance Neo, a Varian 400 MHz Inova NMR spectrometer, or a Varian 600 MHz Inova NMR spectrometer. Chemical shifts (δ) are reported in parts per million (ppm). ^1^H and ^13^C NMR spectra are referenced to TMS using the most downfield protio‐solvent resonance (^1^H NMR C_6_D_6_: δ = 7.16 ppm, ^13^C NMR C_6_D_6_: δ = 128.06 ppm, ^1^H NMR *d*_8_‐toluene: δ = 7.09 ppm). ^2^H{^1^H} spectra are referenced to the naturally abundant deuterated solvent resonances (^2^H NMR C_6_ *D*H: δ = 7.16 ppm, ^2^H NMR C_6_H_5_C*D*H_2_: δ = 2.09 ppm). See Supporting Information for a full assignment of NMR resonances. High‐resolution mass spectra were recorded on a Thermo Q‐Exactive Plus (APCI‐Orbitrap, positive ion mode) instrument at the Mass Spectrometry Facility of the Department of Chemistry of Indiana University. IR spectra were acquired on a Thermo Scientific Nicolet Summit FTIR spectrometer with a diamond ATR stage. A solution/suspension of the samples in pentane was drop‐casted onto the ATR crystal and dried by evaporation inside of a glovebox under a dinitrogen atmosphere prior to the collection of spectra. X‐ray diffraction data were collected with a Bruker D8 Venture diffractometer equipped with a PhotonII detector and IµS sources, using Mo K_α_ radiation and various experiment temperatures, collection strategies, and exposure times. Details are summarized in the CIF and the Supporting Information.

### Synthesis of (InTer)_2_(C_2_H_2_) (1)

A solution of (InTer)_2_ (20 mg, 0.019 mmol) in benzene (0.6 ml) in an NMR tube equipped with a J. Young valve was degassed using the freeze‐pump‐thaw method. Then, the headspace was replaced by 2 atm of acetylene. The solution immediately lightened in color, from deep red to yellow. Conversion to **1** was quantitative by ^1^H NMR spectroscopy. *Compound **1** could not be isolated as a pure solid material; it affords **2** in the presence of excess of acetylene. In the absence of acetylene, compound **1** disproportionates to (InTer)_2_ and **2**. ^1^H NMR (500 MHz, C_6_D_6_): δ (ppm) 9.71 (s, 2H; *H*C = C*H*), 7.27–7.17 (m, 10H; *meta*‐Ter, *para*‐Ter, *para*‐Dipp), 7.06 (d, ^3^ *J*_H─H_ = 7.8 Hz, 8H; *meta*‐Dipp), 3.05 (sept, ^3^ *J*_H─H_ = 6.9 Hz, 8H; C*H*(CH_3_)_2_), 1.13 (d, ^3^ *J*_H─H_ = 6.9 Hz, 24H; CH(C*H*_3_)_2_), 1.10 (d, ^3^ *J*_H─H_ = 6.9 Hz, 24H; CH(C*H*_3_)_2_). ^13^C{^1^H} NMR (125 MHz, C_6_D_6_): δ (ppm) 202.5 (s; H*C *= *C*H), 170.8 (s; *ipso*‐Ter), 146.9 (s; *ortho*‐Dipp), 146.0 (s; *ortho*‐Ter), 141.8 (s; *ipso*‐Dipp), 128.7, 127.8 and 127.4 (s; *meta*‐Ter, *para*‐Ter, *para*‐Dipp), 123.4 (s; *meta*‐Dipp), 30.6 (s; *C*H(CH_3_)_2_), 25.5 (s; CH(*C*H_3_)_2_), 24.1 (s; CH(*C*H_3_)_2_). ^1^H NMR (400 MHz, tol‐d_8_): δ (ppm) 9.61 (s, 2H; *H*C = C*H*), 7.28–7.09 (m, 10H; *meta*‐Ter, *para*‐Ter, *para*‐Dipp, overlapped with toluene resonance), 7.02 (d, ^3^ *J*_H─H_ = 7.8 Hz, 8H; *meta*‐Dipp, overlapped with toluene resonances), 3.00 (sept, ^3^ *J*_H─H_ = 6.9 Hz, 8H; C*H*(CH_3_)_2_), 1.10 and 1.08 (two d, ^3^ *J*_H─H_ = 6.9 Hz, 48H; CH(C*H*_3_)_2_). HR‐MS [APCI‐Orbitrap, positive ion mode]: *m*/*z* for C_62_H_77_In_2_O [**1 **+ O + H]^+^ Calcd: 1067.4046. Found: 1067.4059 (1.16 ppm error).

### Synthesis of (InTer)_2_(C_2_H_2_)_2_ (2)

A solution of (InTer)_2_ (49.8 mg, 0.049 mmol) in benzene (0.6 ml) in an NMR tube equipped with a J. Young valve was degassed using the freeze‐pump‐thaw method. The headspace was subsequently replaced by 2 atm of acetylene. The solution immediately lightened in color, from deep red to a yellow solution of **1**. After 16 h in the presence of acetylene conversion to **2** was quantitative by ^1^H NMR spectroscopy. All volatiles were removed under a dynamic vacuum. The resulting yellow solid was washed with pentane or hexane (3 × 0.2 ml) at −35 °C to yield **2** as a yellow powder. Yield 26.7 mg (0.025 mmol, 51%). Single crystals of **2** suitable for single crystal X‐ray diffraction were obtained by slow evaporation from a concentrated hexane solution of the product at −35 °C. ^1^H NMR (500 MHz, C_6_D_6_): δ (ppm), 7.27–7.19 (m, 10H; *meta*‐Ter, *para*‐Ter, *para*‐Dipp), 7.10 (d, ^3^ *J*_H─H_ = 7.8 Hz, 8H; *meta*‐Dipp), 6.85 (s, 4H; *H*C = C*H*), 3.03 (sept, ^3^ *J*_H─H_ = 6.9 Hz, 8H; C*H*(CH_3_)_2_), 1.17 (d, ^3^ *J*_H─H_ = 6.9 Hz, 24H; CH(C*H*_3_)_2_), 1.06 (d, ^3^ *J*_H─H_ = 6.9 Hz, 24H; CH(C*H*_3_)_2_). ^13^C{^1^H} NMR (125 MHz, C_6_D_6_): δ (ppm) 178.3 (s; H*C *= *C*H), 161.7 (s; *ipso*‐Ter), 147.9 (s; *ortho*‐Ter) 146.7 (s; *ortho*‐Dipp), 142.7 (s; *ipso*‐Dipp), 128.8, 127.5, and 127.3 (s; *meta*‐Ter, *para*‐Ter, *para*‐Dipp), 123.6 (s; *meta*‐Dipp), 30.5 (s; *C*H(CH_3_)_2_), 25.6 (s; CH(*C*H_3_)_2_), 23.6 (s; CH(*C*H_3_)_2_). HR‐MS [APCI‐Orbitrap, positive ion mode]: *m*/*z* for C_64_H_79_In_2_ [**2 **+ H]^+^ Calcd: 1077.4253. Found: 1077.4256 (0.18 ppm error).

### Synthesis of (GaTer)_2_(C_2_H_2_)_2_ (3)

A solution of (GaTer)_2_ (54.7 mg, 0.058 mmol) in benzene (0.6 ml) in an NMR tube equipped with a J. Young valve was degassed using the freeze‐pump‐thaw method. Then, the headspace was replaced by 2 atm of acetylene. The solution immediately lightened in color, from deep green/brown to colorless. Conversion to **3** was deemed to be quantitative by ^1^H NMR spectroscopy. All volatiles were removed in vacuo. The resulting colorless solid was washed with pentane or hexane (3 × 0.2 ml) at −35 °C to yield **3** as a colorless powder. Yield 31.1 mg (0.031 mmol, 54%). Single crystals of **3** suitable for single crystal X‐ray diffraction were obtained by slow evaporation from a concentrated hexane solution of the product at −35 °C. ^1^H NMR (500 MHz, C_6_D_6_): 7.34–7.20 (m, 10H; *meta*‐Ter, *para*‐Ter, *para*‐Dipp), 7.13 (d, ^3^ *J*_H─H_ = 7.9 Hz, 8H; *meta*‐Dipp), 6.69 (s, 4H; *H*C = C*H*), 3.02 (sept, ^3^ *J*_H─H_ = 6.9 Hz, 8H; C*H*(CH_3_)_2_), 1.14 (d, ^3^ *J*_H─H_ = 6.9 Hz, 24H; CH(C*H*_3_)_2_), 1.07 (d, ^3^ *J*_H─H_ = 6.9 Hz, 24H; CH(C*H*_3_)_2_). ^13^C{^1^H} NMR (125 MHz, C_6_D_6_): δ (ppm) 167.1 (s; H*C *= *C*H), 153.5 (s; *ipso*‐Ter), 146.9 (s; *ortho*‐Dipp), 146.7 (s; *ortho*‐Ter), 141.5 (s; *ipso*‐Dipp), 128.7, 127.7 and 127.5 (s; *meta*‐Ter, *para*‐Ter, *para*‐Dipp), 123.4 (s; *meta*‐Dipp), 30.5 (s; *C*H(CH_3_)_2_), 26.0 (s; CH(*C*H_3_)_2_), 23.1 (s; CH(*C*H_3_)_2_). HR‐MS [APCI‐Orbitrap, positive ion mode]: *m*/*z* for C_64_H_79_Ga_2_ [**3 **+ H]^+^ Calcd: 987.4679. Found: 987.4690 (1.08 ppm error).

### Synthesis of (GaTer)_2_(C_2_H_2_) (4)

A solution of (GaTer)_2_ (24.4 mg, 0.026 mmol) in benzene (0.6 ml) in an NMR tube equipped with a J. Young valve was degassed using the freeze‐pump‐thaw method. Then, the headspace was replaced with ∼1 equivalent of acetylene and monitored by ^1^H NMR spectroscopy until complete conversion of the starting material. During this process, the solution gradually lightened in color, from deep green/brown to colorless. All volatiles were removed under a dynamic vacuum. Single crystals of **4** suitable for single crystal X‐ray diffraction were obtained from a concentrated hexane solution (0.3 ml) of the product at −35 °C. Yield 9.8 mg (0.010 mmol, 39%). ^1^H NMR (500 MHz, C_6_D_6_): δ (ppm) 9.36 (s, 2H; *H*C = C*H*), 7.26–7.18 (m, 6H; *para*‐Ter, *para*‐Dipp), 7.10 (d, ^3^ *J*_H─H_ = 7.5 Hz, 4H; *meta*‐Ter), 7.04 (d, ^3^ *J*_H─H_ = 7.8 Hz, 8H; *meta*‐Dipp), 2.98 (sept, ^3^ *J*_H─H_ = 6.9 Hz, 8H; C*H*(CH_3_)_2_), 1.08 and 1.07 (two d, ^3^ *J*_H─H_ = 6.9 Hz, 48H; CH(C*H*_3_)_2_). ^13^C{^1^H} NMR (125 MHz, C_6_D_6_): δ (ppm) 197.3 (s; H*C *= *C*H), 156.5 (s; *ipso*‐Ter), 147.0 (s; *ortho*‐Dipp) 145.3 (s; *ortho*‐Ter), 140.5 (s; *ipso*‐Dipp), *meta*‐Ter, *para*‐Ter and *para*‐Dipp overlapped with benzene resonance, 123.3 (s; *meta*‐Dipp), 30.6 (s; *C*H(CH_3_)_2_), 25.6 (s; CH(*C*H_3_)_2_), 24.0 (s; CH(*C*H_3_)_2_). ^1^H NMR (400 MHz, tol‐d_8_): δ (ppm) 9.28 (s, 2H; *H*C = C*H*), 7.22–7.16 (m, 6H; *para*‐Ter, *para*‐Dipp), 7.06 (d, ^3^ *J*_H─H_ = 7.5 Hz, 6H; *meta*‐Ter, overlapped with toluene resonance), 6.99 (d, ^3^ *J*_H─H_ = 7.8 Hz, 8H; *meta*‐Dipp_,_ overlapped with toluene resonance), 2.92 (sept, ^3^ *J*_H─H_ = 6.9 Hz, 8H; C*H*(CH_3_)_2_), 1.05 and 1.04 (two d, ^3^ *J*_H─H_ = 6.9 Hz, 48H; CH(C*H*_3_)_2_). ^13^C{^1^H} NMR (100 MHz, yol‐d_8_): δ (ppm) 197.4 (s; H*C *= *C*H), 156.4 (s; *ipso*‐Ter), 146.9 (s; *ortho*‐Dipp) 145.3 (s; *ortho*‐Ter), 140.5 (s; *ipso*‐Dipp, *para*‐Ter and *para*‐Dipp, overlapped with toluene resonance), 127.4 (s; *meta*‐Ter), 123.2 (s; *meta*‐Dipp), 30.5 (s; *C*H(CH_3_)_2_), 25.5 (s; CH(*C*H_3_)_2_), 24.0 (s; CH(*C*H_3_)_2_). HR‐MS HR‐MS [APCI‐Orbitrap, positive ion mode]: *m*/*z* for C_62_H_77_Ga_2_O [**4 **+ O + H]^+^ Calcd: 975.4480. Found: 975.4489 (0.84 ppm error).

### Synthesis of (InTer)_2_(C_2_H_2_)_2_(NH_3_)_2_ (5)

A solution of (InTer)_2_(C_2_H_2_)_2_ (**2**) (25.2 mg, 0.023 mmol) in benzene (0.6 ml) in an NMR tube equipped with a J. Young valve was degassed using the freeze‐pump‐thaw method. Then, the headspace was replaced by 2 atm of ammonia. The solution immediately lightened in color, from yellow to colorless forming **5**. All volatiles were removed in vacuo. The resulting colorless solid was washed with pentane or hexane (3 × 0.2 ml) at −35 °C to yield **5** as a colorless powder. Yield 16.8 mg (0.015 mmol, 65%). Single crystals of **5** suitable for single crystal X‐ray diffraction were obtained by slow evaporation from a concentrated benzene solution of the product at room temperature. ^1^H NMR (500 MHz, C_6_D_6_): δ (ppm) 7.39 (s, 4H; *H*C = C*H*), 7.22 (t, ^3^ *J*_H─H_ = 7.4 Hz, 2H; *para*‐Ter), 7.18–7.12 (m, 8H; *meta*‐Ter, *para*‐Dipp, overlapped with benzene resonance), 7.07 (d, ^3^ *J*_H─H_ = 7.7 Hz, 8H; *meta*‐Dipp), 3.12 (sept, ^3^ *J*_H─H_ = 6.9 Hz, 8H; C*H*(CH_3_)_2_), 1.27 (d, ^3^ *J*_H─H_ = 6.9 Hz, 24H; CH(C*H*_3_)_2_), 1.04 (d, ^3^ *J*_H─H_ = 6.9 Hz, 24H; CH(C*H*_3_)_2_), 0.18 (s, 6H, InN*H*_3_). ^13^C{^1^H} NMR (125 MHz, C_6_D_6_): δ (ppm) 173.6 (s; H*C *= *C*H), 159.6 (s; *ipso*‐Ter), 148.2 (s; *ortho*‐Ter), 147.9 (s; *ortho*‐Dipp), 144.5 (s; *ipso*‐Dipp, *meta*‐Ter and *para*‐Dipp overlapped with benzene resonance), 125.7 (s; *para*‐Ter), 122.8 (s; *meta*‐Dipp), 30.5 (s; *C*H(CH_3_)_2_), 25.6 (s; CH(*C*H_3_)_2_), 23.3 (s; CH(*C*H_3_)_2_). HR‐MS [APCI‐Orbitrap, positive ion mode]: not detected.

### Synthesis of (InTer)_2_(C_2_H_2_)_2_(ND_3_)_2_ (5_D_)

A solution of (InTer)_2_(C_2_H_2_)_2_ (**2**) (30 mg, 0.027 mmol) in benzene (0.6 ml) in an NMR tube equipped with a J. Young valve was degassed using the freeze‐pump‐thaw method. Then, the headspace was replaced by 1 atm of deuterated ammonia (ND_3_). The solution immediately lightened in color, from yellow to colorless forming **5_D_**. All volatiles were removed in vacuo. The resulting colorless solid was washed with pentane (3 × 0.2 ml) at −35 °C to yield **5_D_** as a colorless powder. Yield 19.4 mg (0.017 mmol, 62%). ^1^H NMR (500 MHz, C_6_D_6_): δ (ppm) 7.39 (s, 4H; *H*C = C*H*), 7.22 (t, ^3^ *J*_H─H_ = 7.4 Hz, 2H; *para*‐Ter), 7.18–7.12 (m, 8H; *meta*‐Ter, *para*‐Dipp, overlapped with benzene resonance), 7.07 (d, ^3^ *J*_H─H_ = 7.7 Hz, 8H; *meta*‐Dipp), 3.12 (sept, ^3^ *J*_H─H_ = 6.9 Hz, 8H; C*H*(CH_3_)_2_), 1.27 (d, ^3^ *J*_H─H_ = 6.9 Hz, 24H; CH(C*H*_3_)_2_), 1.04 (d, ^3^ *J*_H─H_ = 6.9 Hz, 24H; CH(C*H*_3_)_2_), 0.15 (residual In‐N*H*D_2_). ^2^H{^1^H} NMR (61 MHz, C_6_H_6_): δ (ppm) 0.10 (s; In‐N*D*_3_). ^1^H NMR (500 MHz, Tol‐d_8_): δ (ppm) 7.28 (s, 4H, *H*C = C*H*), 7.20 (t, ^3^ *J*_H─H_ = 7.4 Hz, 2H, *para*‐Ter), 7.14 (t, ^3^ *J*_H─H_ = 7.6 Hz, 4H, *para*‐Dipp), 7.08 (d, ^3^ *J*_H─H_ = 7.4 Hz, 4H, *meta*‐Ter, overlapped with benzene resonance), 7.04 (d, ^3^ *J*_H─H_ = 7.6 Hz, 8H, *meta*‐Dipp), 3.07 (sept, ^3^ *J*_H─H_ = 6.9 Hz, 8H, C*H*(CH_3_)_2_), 1.23 (d, ^3^ *J*_H─H_ = 6.9 Hz, 24H, CH(C*H*_3_)_2_), 1.04 (d, ^3^ *J*_H─H_ = 6.9 Hz, 24H, CH(C*H*_3_)_2_), 0.09 (residual In‐N*H*D_2_). ^2^H{^1^H} NMR (92 MHz, Tol): δ (ppm) 0.05 (s; In‐N*D*_3_).

### Synthesis of (GaTer)_2_(C_2_H_2_)_2_(NH_3_)_2_ (6)

A solution of (GaTer)_2_(C_2_H_2_)_2_ (**3**) (30 mg, 0.030 mmol) in benzene (0.6 ml) in an NMR tube equipped with a J. Young valve was degassed using the freeze‐pump‐thaw method. Then, the headspace was replaced by 2 atm of ammonia. All volatiles were removed in vacuo. The resulting colorless solid was washed with pentane or hexane (3 × 0.2 ml) at −35 °C to yield **6** as a colorless powder. Yield 25.5 mg (0.024 mmol, 82%). Single crystals of **6** suitable for single crystal X‐ray diffraction were obtained by slow evaporation from a concentrated benzene solution of the product at room temperature. ^1^H NMR (500 MHz, C_6_D_6_): δ (ppm) 7.30 (s, 4H; *H*C = C*H*), 7.19 (t; ^3^ *J*_H─H_ = 7.7 Hz, 2H; *para*‐Ter), 7.13 (t, ^3^ *J*_H─H_ = 7.4 Hz, 4H; *para*‐Dipp), 7.05 (d, ^3^ *J*_H─H_ = 7.7 Hz, 4H; *meta*‐Ter), 7.03 (d, ^3^ *J*_H─H_ = 7.4 Hz, 8H; *meta*‐Dipp), 3.13 (sept, ^3^ *J*_H─H_ = 6.9 Hz, 8H; C*H*(CH_3_)_2_), 1.28 (d, ^3^ *J*_H─H_ = 6.9 Hz, 24H; CH(C*H*_3_)_2_), 1.03 (d, ^3^ *J*_H─H_ = 6.9 Hz, 24H; CH(C*H*_3_)_2_), 0.31 (s, 6H; GaN*H*_3_). ^13^C{^1^H} NMR (125 MHz, C_6_D_6_): δ (ppm) 164.4 (s; H*C *= *C*H), 152.5 (s; *ipso*‐Ter), 148.0 (s; *ortho*‐Dipp), 147.0 (s; *ortho*‐Ter), 144.4 (s; *ipso*‐Dipp), 128.6 (s; *para*‐Dipp), 127.6 (s; *meta*‐Ter), 125.3 (s; *para*‐Ter), 122.6 (s; *meta*‐Dipp), 30.4 (s; *C*H(CH_3_)_2_), 25.6 (s; CH(*C*H_3_)_2_), 23.1 (s; CH(*C*H_3_)_2_). HR‐MS [APCI‐Orbitrap, positive ion mode]: *m*/*z* for C_64_H_82_Ga_2_N [**6**‐NH_3 _+ H]^+^ Calcd: 1002.4961. Found: 1002.4961 (0.74 ppm error).

### Synthesis of (InTer)_2_(C_2_H_2_)(μ‐NH_2_)_2_ (7)

A solution of (InTer)_2_(C_2_H_2_)_2_(NH_3_)_2_ (**5**) (16.3 mg, 0.014 mmol) in benzene (0.6 ml) in an NMR tube equipped with a J. Young valve was heated at 80 °C for 6 h. All volatiles were removed in vacuo. The resulting colorless solid was washed with pentane or hexane (3 × 0.2 ml) at −35 °C to yield **7** as a colorless powder. Yield 10.1 mg (0.009 mmol, 64%). Single crystals of **7** suitable for single crystal X‐ray diffraction were obtained from a concentrated hexane solution of the product at −35 °C. ^1^H NMR (500 MHz, C_6_D_6_): δ (ppm) 7.53 (s, 2H; *H*C = C*H*), 7.26–7.19 (m, 10H; *meta*‐Ter, *para*‐Ter, *para*‐Dipp), 7.10 (d, ^3^ *J*_H─H_ = 7.7 Hz, 8H; *meta*‐Dipp), 2.96 (sept, ^3^ *J*_H─H_ = 6.9 Hz, 8H; C*H*(CH_3_)_2_), 1.15 (d, ^3^ *J*_H─H_ = 6.9 Hz, 24H; CH(C*H*_3_)_2_), 1.08 (d, ^3^ *J*_H─H_ = 6.9 Hz, 24H; CH(C*H*_3_)_2_), −0.96 (d, 2H, ^2^ *J*_H─H_ = 9.6 Hz; N*H*_2_), −1.86 (d, 2H, ^2^ *J*_H─H_ = 9.6 Hz; N*H*_2_). ^13^C{^1^H} NMR (125 MHz, C_6_D_6_): δ (ppm) 167.7 (s; H*C *= *C*H), 155.6 (s; *ipso*‐Ter), 148.8 (s; *ortho*‐Ter), 146.8 (s; *ortho*‐Dipp), 143.6 (s; *ipso*‐Dipp, *meta*‐Ter or *para*‐Dipp overlapped with benzene resonance), 127.4 (s; *meta*‐Ter or *para*‐Dipp), 127.2 (s; *para*‐Ter), 123.1 (s; *meta*‐Dipp), 30.6 (s; *C*H(CH_3_)_2_), 25.6 (s; CH(*C*H_3_)_2_), 23.3 (s; CH(*C*H_3_)_2_). HR‐MS [APCI‐Orbitrap, positive ion mode]: *m*/*z* for C_62_H_81_N_2_In_2_ [**7 **+ H]^+^ Calcd: 1083.4471. Found: 1083.4455 (−1.53 ppm error).

### *S*ynthesis of (InTer)_2_(C_2_H_2_)(μ‐ND_2_)_2_ (7_D_)

A solution of (InTer)_2_(C_2_H_2_)_2_(ND_3_)_2_ (**5_D_**) (4 mg, 0.0035 mmol) in toluene (0.6 ml) in an NMR tube equipped with a J. Young valve was heated at 110 °C for 12 h (or at 80 °C for 30 h in benzene). All volatiles were removed in vacuo. Due to the small reaction scale, compound **7_D_** was isolated without further purification. ^1^H NMR (500 MHz, C_6_D_6_): δ (ppm) 7.53 (s, 2H; *H*C = C*H*), 7.26–7.19 (m, 10H; *meta*‐Ter, *para*‐Ter, *para*‐Dipp), 7.10 (d, ^3^ *J*_H─H_ = 7.7 Hz, 8H; *meta*‐Dipp), 2.97 (sept, ^3^ *J*_H─H_ = 6.9 Hz, 8H; C*H*(CH_3_)_2_), 1.15 (d, ^3^ *J*_H─H_ = 6.9 Hz, 24H; CH(C*H*_3_)_2_), 1.08 (d, ^3^ *J*_H─H_ = 6.9 Hz, 24H; CH(C*H*_3_)_2_), −0.98 (br, residual In‐N*H*D‐In), −1.88 (br, residual In‐N*H*D‐In). ^1^H NMR (500 MHz, Tol‐d_8_): δ (ppm) 7.41 (s, 2H; *H*C = C*H*), 7.26–7.17 (m, 10H; *meta*‐Ter, *para*‐Ter, *para*‐Dipp), 7.07 (d, ^3^ *J*_H─H_ = 7.7 Hz, 8H; *meta*‐Dipp), 2.92 (sept, ^3^ *J*_H─H_ = 6.9 Hz, 8H; C*H*(CH_3_)_2_), 1.12 (d, ^3^ *J*_H─H_ = 6.9 Hz, 24H; CH(C*H*_3_)_2_), 1.06 (d, ^3^ *J*_H─H_ = 6.9 Hz, 24H; CH(C*H*_3_)_2_), −1.04 (br, residual In‐N*H*D‐In), −1.96 (br, residual In‐N*H*D‐In). ^2^H{^1^H} NMR (92 MHz, Tol): δ (ppm) −1.05 (br, 2D, In‐N*D*_2_‐In), −2.01 (br, 2D, In‐N*D*_2_‐In).

### Synthesis of (GaTer)_2_(C_2_H_2_)(μ‐NH_2_)_2_ (8)

A solution of (GaTer)_2_(C_2_H_2_)_2_(NH_3_)_2_ (**6**) (25.5 mg, 0.025 mmol) in toluene (0.6 ml) in an NMR tube equipped with a J. Young valve was heated at 110 °C for 8 days. All volatiles were removed under vacuum. The resulting colorless solid was washed with pentane or hexane (3 × 0.2 ml) at −35°C to yield **8** as a colorless powder. Yield 12.7 mg (0.013 mmol, 51%). Despite extensive efforts, a pure sample of **8** could not be isolated (unknown impurity <15%). Single crystals of **8** suitable for single crystal X‐ray diffraction were obtained from a concentrated hexane solution of the product at −35 °C. ^1^H NMR (500 MHz, C_6_D_6_): δ (ppm) 7.42 (s, 2H; H*C *= *C*H), 7.27–7.16 (m, 10H; *meta*‐Ter, *para*‐Ter, *para*‐Dipp, overlapped with benzene resonance), 7.07 (d, ^3^ *J*_H─H_ = 7.7 Hz, 8H; *meta*‐Dipp), 2.90 (sept, ^3^ *J*_H─H_ = 6.9 Hz, 8H; C*H*(CH_3_)_2_), 1.11 (d, ^3^ *J*_H─H_ = 6.9 Hz, 24H; CH(C*H*_3_)_2_), 1.06 (d, ^3^ *J*_H─H_ = 6.9 Hz, 24H; CH(C*H*_3_)_2_), −0.57 (d, 2H, ^2^ *J*_H─H_ = 9.9 Hz; NH_2_), −1.54 (d, 2H, ^2^ *J*_H─H_ = 9.9 Hz, 2H; N*H*_2_). ^13^C{^1^H} NMR (125 MHz, C_6_D_6_): δ (ppm) 163.1 (s; H*C *= *C*H), 147.8 (s; *ortho*‐Ter), 146.8 (s; *ipso*‐Ter), 146.7 (s; *ortho*‐Dipp), 142.4 (s; *ipso*‐Dipp, *meta*‐Ter and *para*‐Dipp overlapped with benzene resonance), 127.1 (s; *para*‐Ter), 122.7 (s; *meta*‐Dipp), 30.5 (s; *C*H(CH_3_)_2_), 25.7 (s; CH(*C*H_3_)_2_), 23.2 (s; CH(*C*H_3_)_2_). HR‐MS [APCI‐Orbitrap, positive ion mode]: *m*/*z* for C_62_H_81_N_2_Ga_2_ [**8 **+ H]^+^ Calcd: 993.4897. Found: 993.4922 (2.51 ppm error).

## Supporting Information

The Supporting Information for this article contains spectra, computational information, crystallographic data, and coordinates for all optimized structures (.xyz). The authors have cited additional references within the Supporting Information.^[^ ^57^, ^58^, ^59^, ^60^, ^61^, ^62^, ^63^, ^64^, ^65^, ^66^, ^67^, ^68^, ^69^ ^]^

## Conflict of Interests

The authors declare no conflict of interest.

## Supporting information



Supporting Information

## Data Availability

The data that support the findings of this study are available in the Supporting Information of this article.
