# Supplementary material for: Stepwise Acetylene Insertion and Ammonia Activation at a Digallene and Diindene
Source: Angew Chem Int Ed Engl. 2025 Jul 4;64(34):e202509661. doi: 10.1002/anie.202509661 (PMC12363634; doi:10.1002/anie.202509661)
Supplement: Supplementary file 1 — Supporting Information [file ANIE-64-e202509661-s001.docx]

Contents

[1. NMR spectra 2](#_Toc200368692)

[1.1 NMR spectra of compound **1** 2](#_Toc200368693)

[1.2 NMR spectra of compound **2** 5](#_Toc200368694)

[1.3 NMR spectra of compound **3** 8](#_Toc200368695)

[1.4 NMR spectra of compound **4** 11](#_Toc200368696)

[1.5 NMR spectra of compound **5** 15](#_Toc200368697)

[1.6 NMR spectra of compound **5_D_** 18](#_Toc200368698)

[1.7 NMR spectra of compound **6** 21](#_Toc200368699)

[1.8 NMR spectra of compound **7** 24](#_Toc200368700)

[1.9 NMR spectra of compound **7_D_** 27](#_Toc200368701)

[1.10 NMR spectra of compound **8** 30](#_Toc200368702)

[2. *In situ* NMR Studies 32](#_Toc200368703)

[3. Infrared spectra 36](#_Toc200368704)

[4. High resolution mass data 41](#_Toc200368705)

[5. X-ray crystallographic studies 45](#_Toc200368706)

[6. Computational details 69](#_Toc200368707)

[7. References 105](#_Toc200368708)

# NMR spectra

## NMR spectra of compound **1**

**Figure S1.** Compound **1**, (InTer)_2_(C_2_H_2_), with the atom labelling used for the assignment of NMR spectra.

**^1^H NMR (500 MHz, C_6_D_6_):** δ (ppm) 9.71 (s, 2H, H_12_), 7.27-7.17 (m, 10H, H_3_+H_4_+H_8_), 7.06 (d, ^3^*J*_H–H_ = 7.8 Hz, 8H, H_7_), 3.05 (sept, ^3^*J*_H–H_ = 6.9 Hz, 8H, H_9_, CH(CH_3_)_2_), 1.13 (d, ^3^*J*_H–H_ = 6.9 Hz, 24H, H_10_ or H_11_, CH(CH_3_)_2_), 1.10 (d, ^3^*J*_H–H_ = 6.9 Hz, 24H, H_10_ or H_11_, CH(CH_3_)_2_).

**^13^C{^1^H} NMR (125 MHz, C_6_D_6_):** δ (ppm) 202.5 (s, C_12_), 170.8 (s, C_1_), 146.9 (s, C_6_) 146.0 (s, C_2_), 141.8 (s, C_5_), 128.7, 127.8 and 127.4 (s, C_3,_ C_4_ and C_8_), 123.4 (s, C_7_), 30.6 (s, C_9_), 25.5 (s, C_10_ or C_11_, CH(CH_3_)_2_), 24.1 (s, C_10_ or C_11_, CH(CH_3_)_2_).

**^1^H NMR (400 MHz, Tol-d_8_):** δ (ppm) 9.61 (s, 2H, H_12_), 7.28-7.09 (m, 10H, H_3_+H_4_+H_8_, overlapped with toluene resonance), 7.02 (d, ^3^*J*_H–H_ = 7.8 Hz, 8H, H_7_, overlapped with toluene resonances), 3.00 (sept, ^3^*J*_H–H_ = 6.9 Hz, 8H, H_9_, CH(CH_3_)_2_), 1.10 and 1.08 (two d, ^3^*J*_H–H_ = 6.9 Hz, 48H, H_10_ and H_11_, CH(CH_3_)_2_).


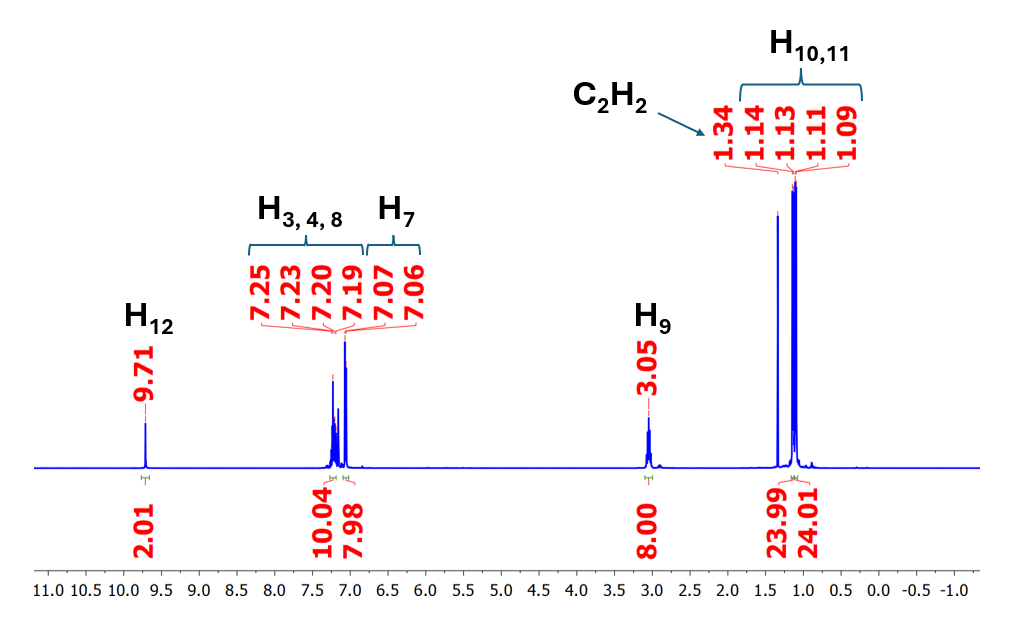


**Figure S2.** ^1^H NMR (298 K, C_6_D_6_, 500 MHz) spectrum of **1**. Note: acetylene resonance at 1.34 ppm.


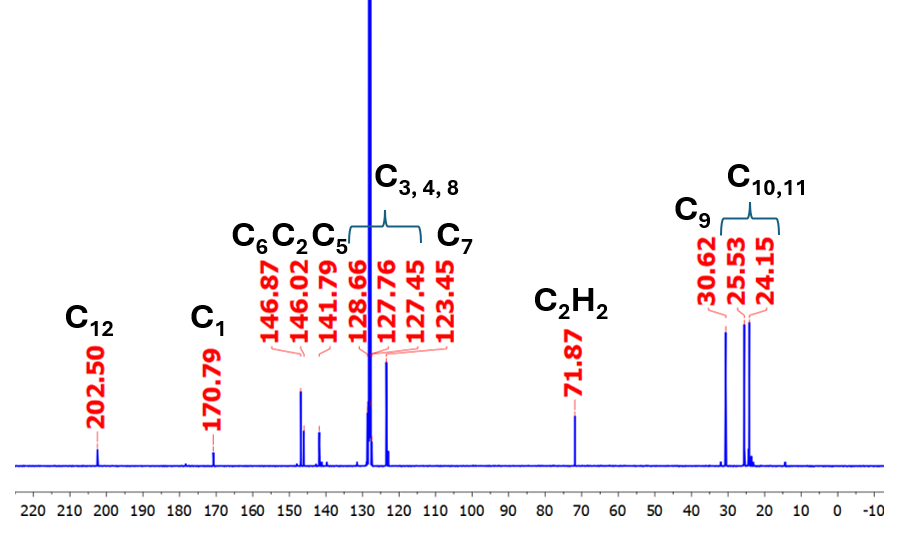


**Figure S3.** ^13^C{^1^H} NMR (298 K, C_6_D_6_, 125 MHz) spectrum of **1**. Note: acetylene resonance at 71.87 ppm.


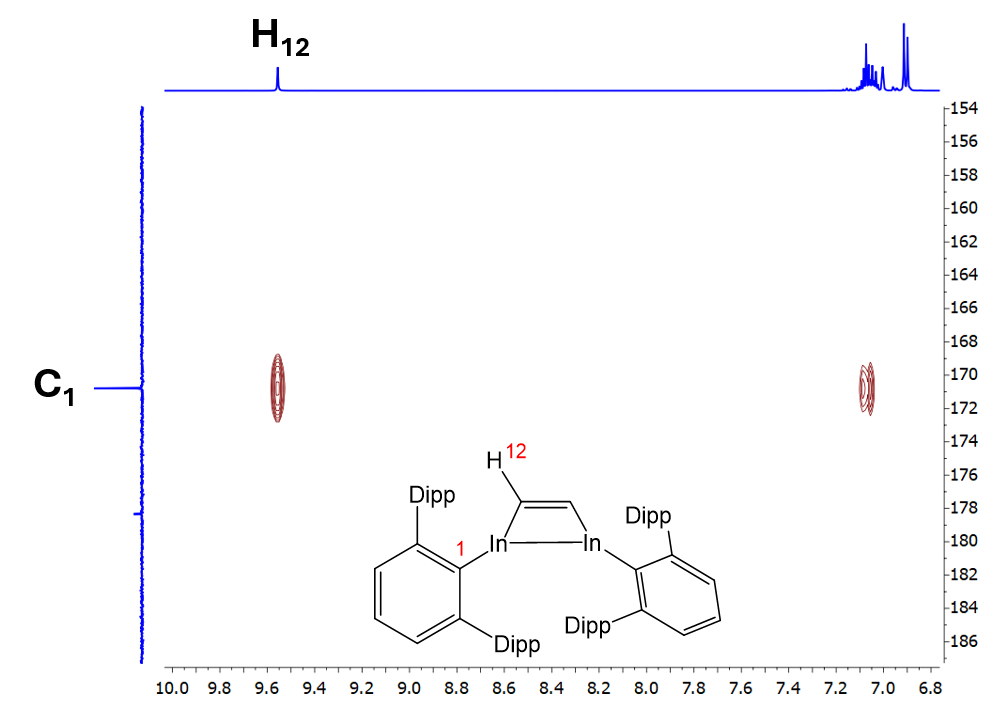


**Figure S4.** Selected region of the ^1^H-^13^C HMBC (298K, C_6_D_6_ 125 MHz) spectrum of **1** showing the correlation between H_12_ and C_1_.
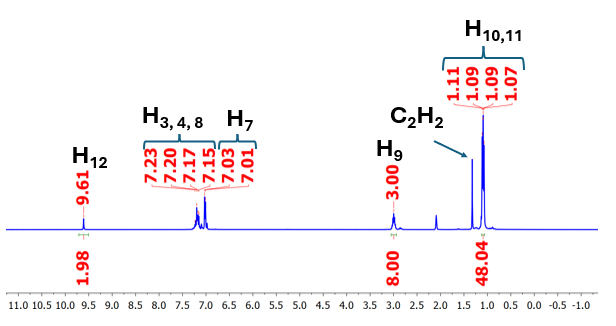


**Figure S5.** ^1^H NMR (298 K, Tol-d_8_, 400 MHz) spectrum of **1**.

## NMR spectra of compound **2**

**Figure S6.** Compound **2**, (InTer)_2_(C_2_H_2_)_2_, with the atom labelling used for the assignment of NMR spectra.

**^1^H NMR (500 MHz, C_6_D_6_):** δ (ppm), 7.27-7.19 (m, 10H, H_3_+H_4_+H_8_), 7.10 (d, ^3^*J*_H–H_ = 7.8 Hz, 8H, H_7_), 6.85 (s, 4H, H_12_), 3.03 (sept, ^3^*J*_H–H_ = 6.9 Hz, 8H, H_9_, CH(CH_3_)_2_), 1.17 (d, ^3^*J*_H–H_ = 6.9 Hz, 24H, H_10_ or H_11_, CH(CH_3_)_2_), 1.06 (d, ^3^*J*_H–H_ = 6.9 Hz, 24H, H_10_ or H_11_, CH(CH_3_)_2_).

**^13^C{^1^H} NMR (125 MHz, C_6_D_6_):** δ (ppm) 178.3 (s, C_12_), 161.7 (s, C_1_), 147.9 (s, C_2_) 146.7 (s, C_6_), 142.7 (s, C_5_), 128.8, 127.5 and 127.3 (s, C_3,_ C_4_ and C_8_), 123.6 (s, C_7_), 30.5 (s, C_9_), 25.6 (s, C_10_ or C_11_, CH(CH_3_)_2_), 23.6 (s, C_10_ or C_11_, CH(CH_3_)_2_).


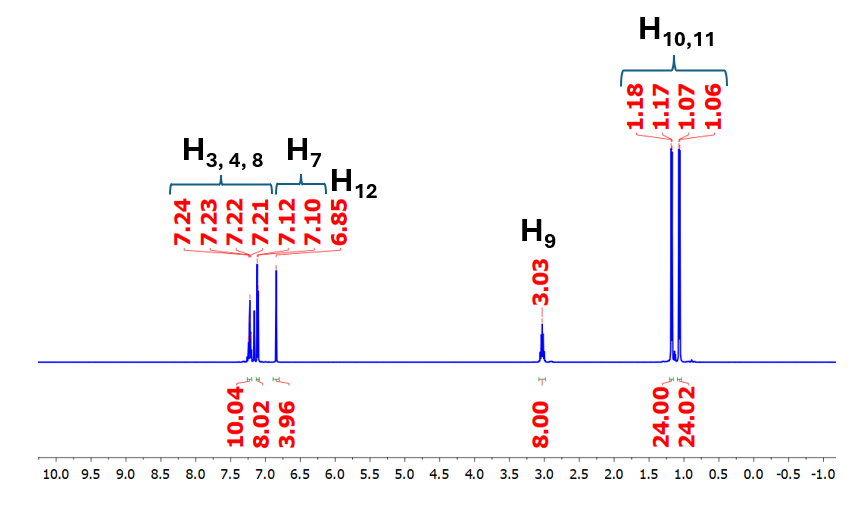


**Figure S7.** ^1^H NMR (298 K, C_6_D_6_, 500 MHz) spectrum of **2**.


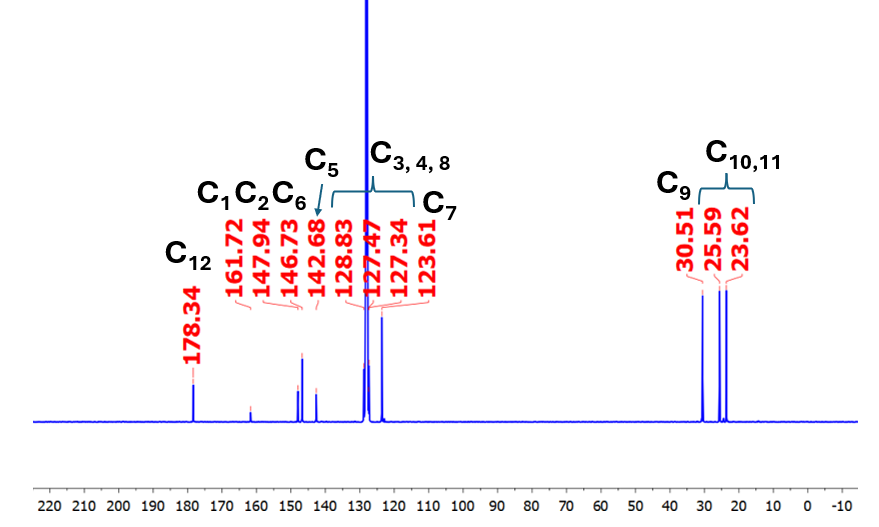


**Figure S8.** ^13^C{^1^H} NMR (298 K, C_6_D_6_, 125 MHz) spectrum of **2**.


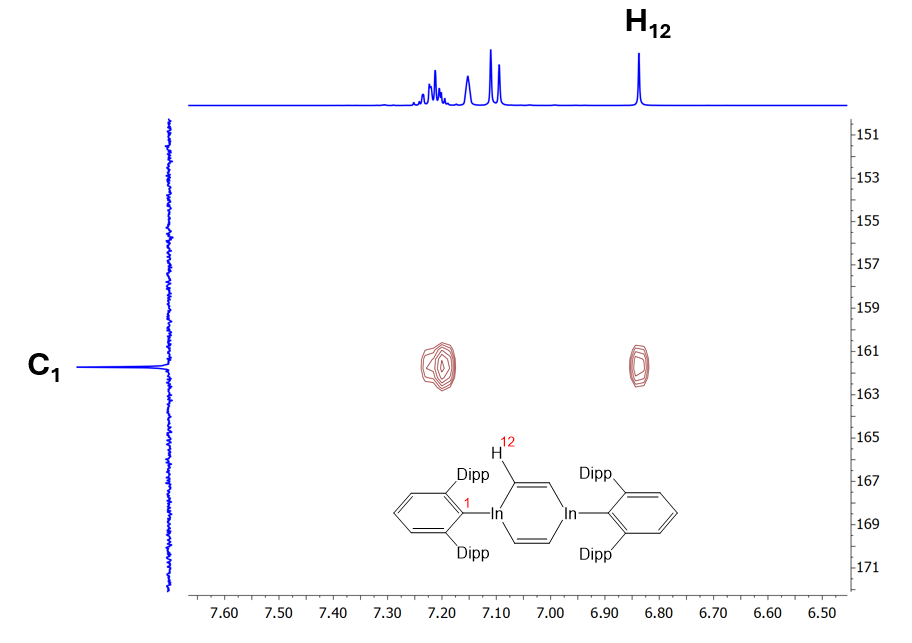


**Figure S9.** Selected region of the ^1^H-^13^C HMBC (298K, C_6_D_6_ 125 MHz) spectrum of **2** showing the correlation between H_12_ and C_1_.

## NMR spectra of compound **3**

**Figure S10.** Compound **3**, (GaTer)_2_(C_2_H_2_)_2_, with the atom labelling used for the assignment of NMR spectra.

**^1^H NMR (500 MHz, C_6_D_6_):** δ (ppm), 7.34-7.20 (m, 10H, H_3_+H_4_+H_8_), 7.13 (d, ^3^*J*_H–H_ = 7.9 Hz, 8H, H_7_), 6.69 (s, 4H, H_12_), 3.02 (sept, ^3^*J*_H–H_ = 6.9 Hz, 8H, H_9_, CH(CH_3_)_2_), 1.14 (d, ^3^*J*_H–H_ = 6.9 Hz, 24H, H_10_ or H_11_, CH(CH_3_)_2_), 1.07 (d, ^3^*J*_H–H_ = 6.9 Hz, 24H, H_10_ or H_11_, CH(CH_3_)_2_).

**^13^C{^1^H} NMR (125 MHz, C_6_D_6_):** δ (ppm) 167.1 (s, C_12_), 153.5 (s, C_1_), 146.9 (s, C_6_) 146.7 (s, C_2_), 141.5 (s, C_5_), 128.7, 127.7 and 127.5 (s, C_3,_ C_4_ and C_8_), 123.4 (s, C_7_), 30.5 (s, C_9_), 26.0 (s, C_10_ or C_11_, CH(CH_3_)_2_), 23.1 (s, C_10_ or C_11_, CH(CH_3_)_2_).


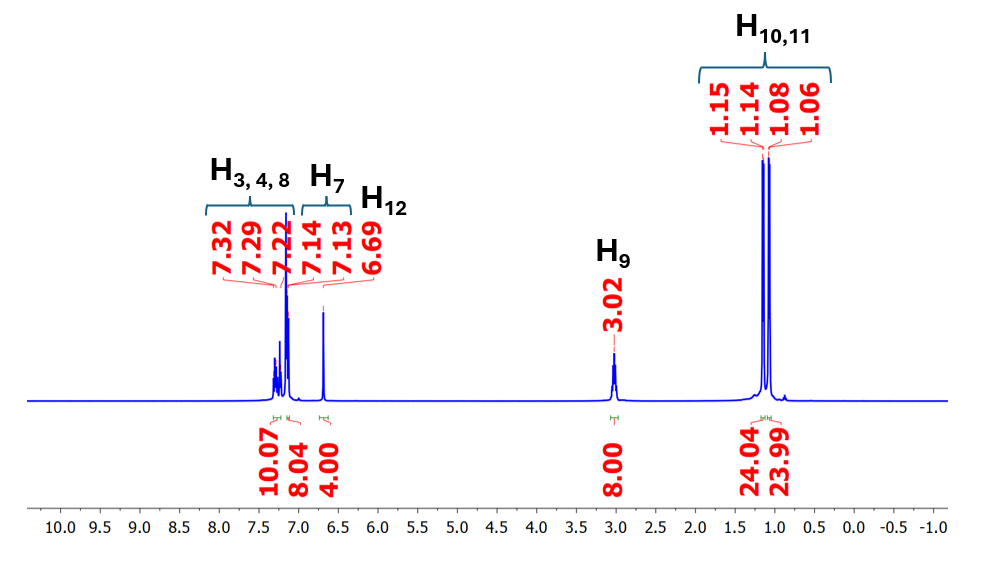


**Figure S11.** ^1^H NMR (298 K, C_6_D_6_, 500 MHz) spectrum of **3**.


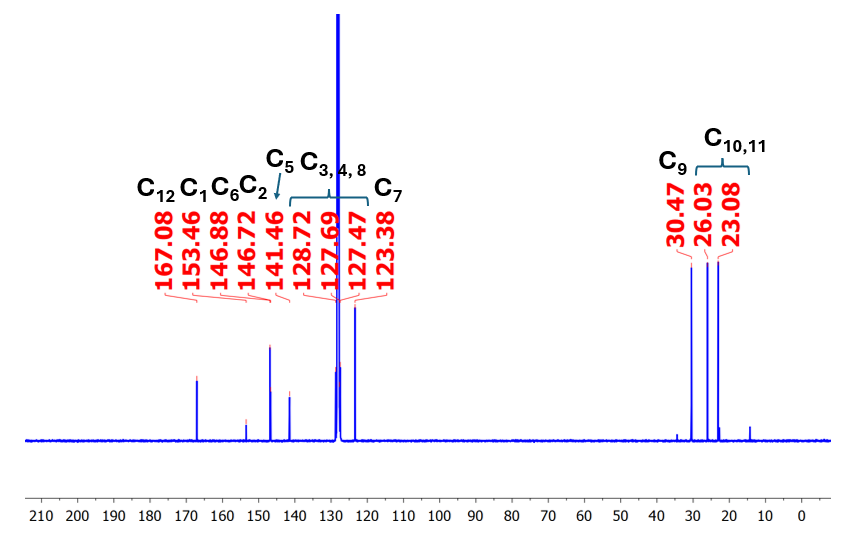


**Figure S12.** ^13^C{^1^H} NMR (298 K, C_6_D_6_, 125 MHz) spectrum of **3**.


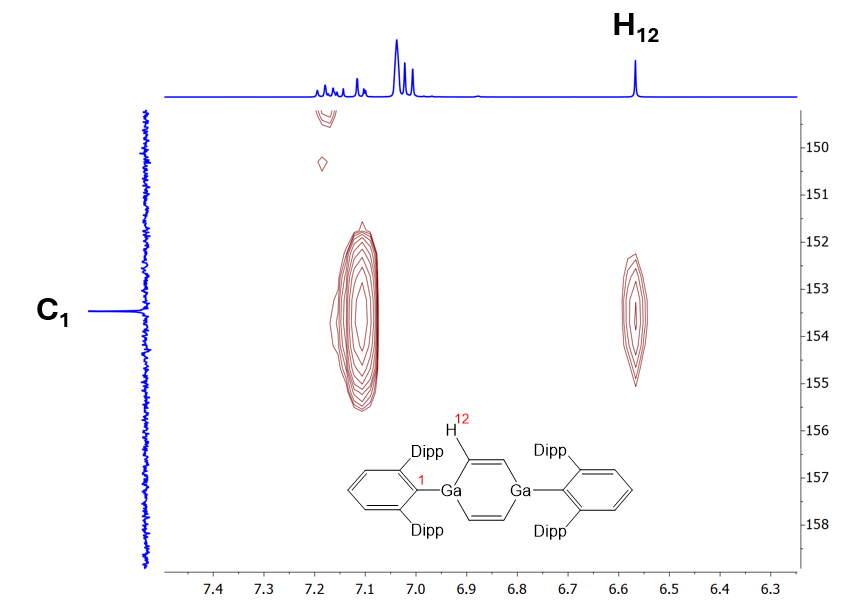


**Figure S13.** Selected region of the ^1^H-^13^C HMBC (298K, C_6_D_6_ 125 MHz) spectrum of **3** showing the correlation between H_12_ and C_1_.

## NMR spectra of compound **4**

**Figure S14.** Compound **4**, (GaTer)_2_(C_2_H_2_), with the atom labelling used for the assignment of NMR spectra.

**^1^H NMR (500 MHz, C_6_D_6_):** δ (ppm) 9.36 (s, 2H, H_12_), 7.26-7.18 (m, 6H, H_4_+H_8_), 7.10 (d, ^3^*J*_H–H_ = 7.5 Hz, 4H, H_3_), ), 7.04 (d, ^3^*J*_H–H_ = 7.8 Hz, 8H, H_7_), 2.98 (sept, ^3^*J*_H–H_ = 6.9 Hz, 8H, H_9_, CH(CH_3_)_2_), 1.08 and 1.07 (d, ^3^*J*_H–H_ = 6.9 Hz, 48H, H_10_ and H_11_, CH(CH_3_)_2_).

**^13^C{^1^H} NMR (125 MHz, C_6_D_6_):** δ (ppm) 197.3 (s, C_12_), 156.5 (s, C_1_), 147.0 (s, C_6_) 145.3 (s, C_2_), 140.5 (s, C_5_), C_3,_ C_4_ and C_8_ overlapped with benzene resonance, 123.3 (s, C_7_), 30.6 (s, C_9_), 25.6 (s, C_10_ or C_11_, CH(CH_3_)_2_), 24.0 (s, C_10_ or C_11_, CH(CH_3_)_2_).

**^1^H NMR (400 MHz, Tol-d_8_):** δ (ppm) 9.28 (s, 2H, H_12_), 7.22-7.16 (m, 6H, H_4_+H_8_), 7.06 (d, ^3^*J*_H–H_ = 7.5 Hz, 6H, H_3_, overlapped with toluene resonances), 6.99 (d, ^3^*J*_H–H_ = 7.8 Hz, 8H, H_7,_ overlapped with toluene resonances), 2.92 (sept, ^3^*J*_H–H_ = 6.9 Hz, 8H, H_9_, CH(CH_3_)_2_), 1.05 and 1.04 (d, ^3^*J*_H–H_ = 6.9 Hz, 48H, H_10_ and H_11_, CH(CH_3_)_2_).

**^13^C{^1^H} NMR (100 MHz, Tol-d_8_):** δ (ppm) 197.4 (s, C_12_), 156.4 (s, C_1_), 146.9 (s, C_6_) 145.3 (s, C_2_), 140.5 (s, C_5_), C_4_ and C_8_ overlapped with toluene resonance, 127.4 (s, C_3_), 123.2 (s, C_7_), 30.5 (s, C_9_), 25.5 (s, C_10_ or C_11_, CH(CH_3_)_2_), 24.0 (s, C_10_ or C_11_, CH(CH_3_)_2_).


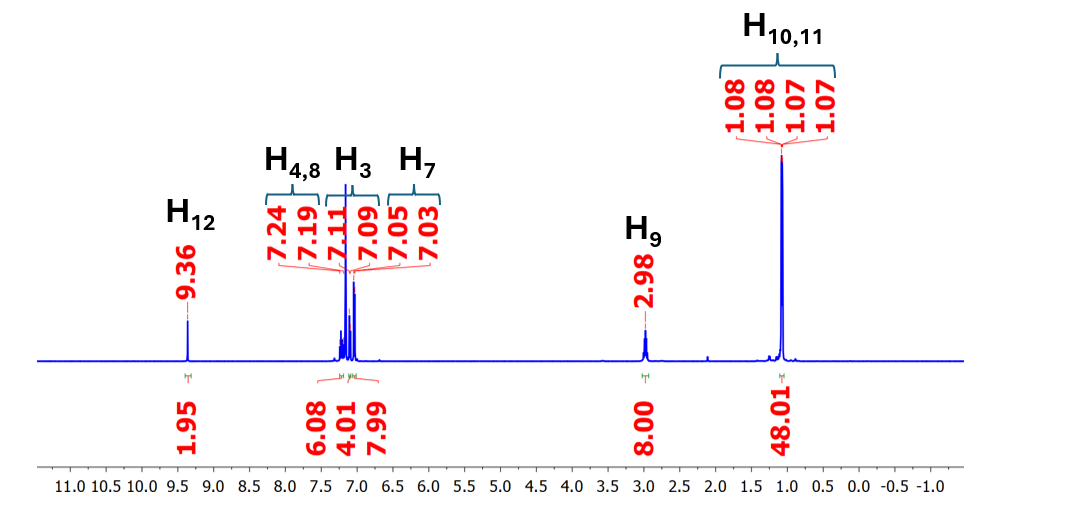


**Figure S15.** ^1^H NMR (298 K, C_6_D_6_, 500 MHz) spectrum of **4**.


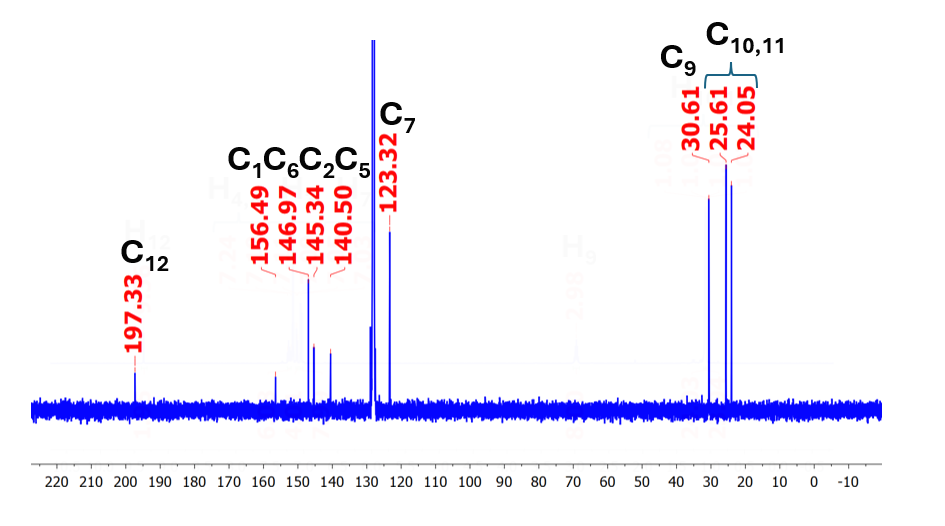


**Figure S16.** ^13^C{^1^H} NMR (298 K, C_6_D_6_, 125 MHz) spectrum of **4**.


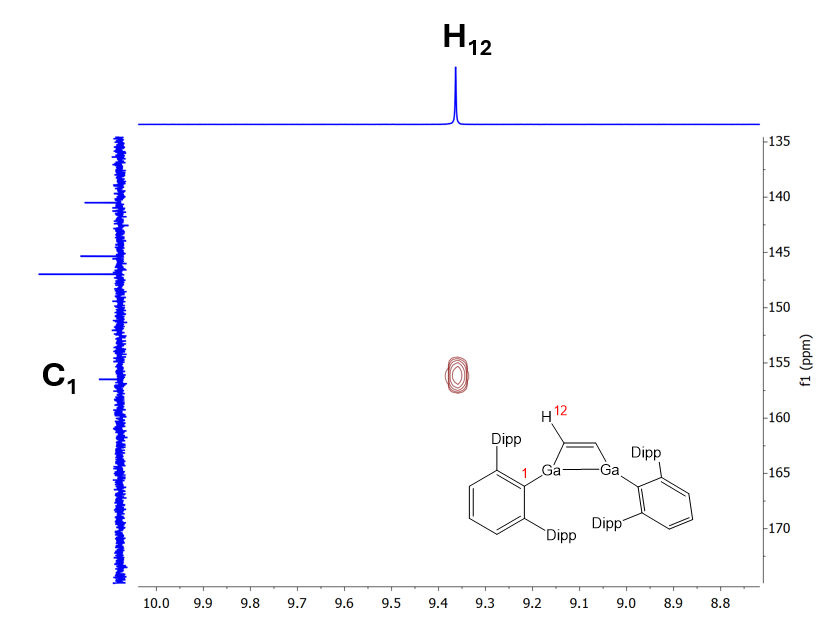


**Figure S17.** Selected region of the ^1^H-^13^C HMBC (298K, C_6_D_6_ 125 MHz) spectrum of **4** showing the correlation between H_12_ and C_1_.


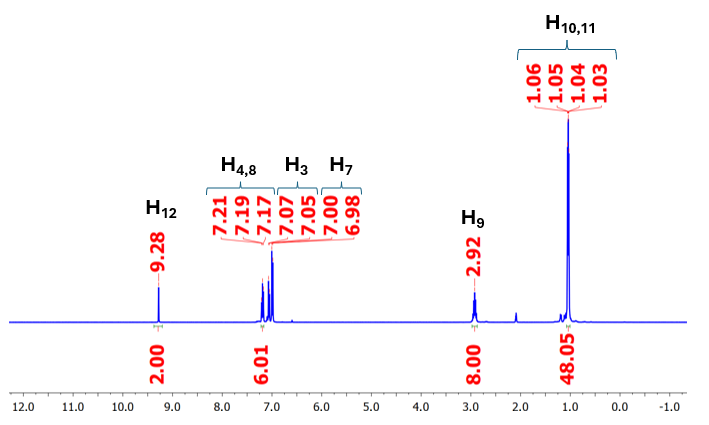


**Figure S18.** ^1^H NMR (298 K, Tol-d_8_, 400 MHz) spectrum of **4**.


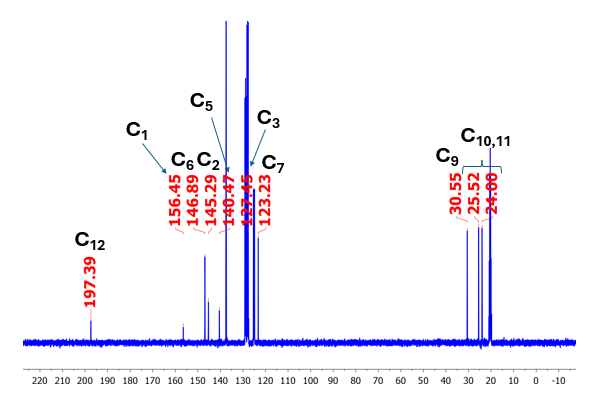


**Figure S19.** ^13^C{^1^H} NMR (298 K, Tol-d_8_, 100 MHz) spectrum of **4**.

## NMR spectra of compound **5**

**Figure S20.** Compound **5**, (InTer)_2_(C_2_H_2_)_2_(NH_3_)_2_, with the atom labelling used for the assignment of NMR spectra.

**^1^H NMR (500 MHz, C_6_D_6_):** δ (ppm) 7.39 (s, 4H, H_12_), 7.22 (t, ^3^*J*_H–H_ = 7.4 Hz, 2H, H_4_), 7.18-7.12 (m, 8H, H_3_+H_8_, overlapped with benzene resonance), 7.07 (d, ^3^*J*_H–H_ = 7.7 Hz, 8H, H_7_), 3.12 (sept, ^3^*J*_H–H_ = 6.9 Hz, 8H, H_9_, CH(CH_3_)_2_), 1.27 (d, ^3^*J*_H–H_ = 6.9 Hz, 24H, H_10_ or H_11_, CH(CH_3_)_2_), 1.04 (d, ^3^*J*_H–H_ = 6.9 Hz, 24H, H_10_ or H_11_, CH(CH_3_)_2_), 0.18 (s, 6H, H_13_, In-NH_3_).

**^13^C{^1^H} NMR (125 MHz, C_6_D_6_):** δ (ppm) 173.6 (s, C_12_), 159.6 (s, C_1_), 148.2 (s, C_2_), 147.9 (s, C_6_), 144.5 (s, C_5_), C_3_ and C_8_ overlapped with benzene resonance, 125.7 (s, C_4_), 122.8 (s, C_7_), 30.46 (s, C_9_), 25.6 (s, C_10_ or C_11_, CH(CH_3_)_2_), 23.3 (s, C_10_ or C_11_, CH(CH_3_)_2_).


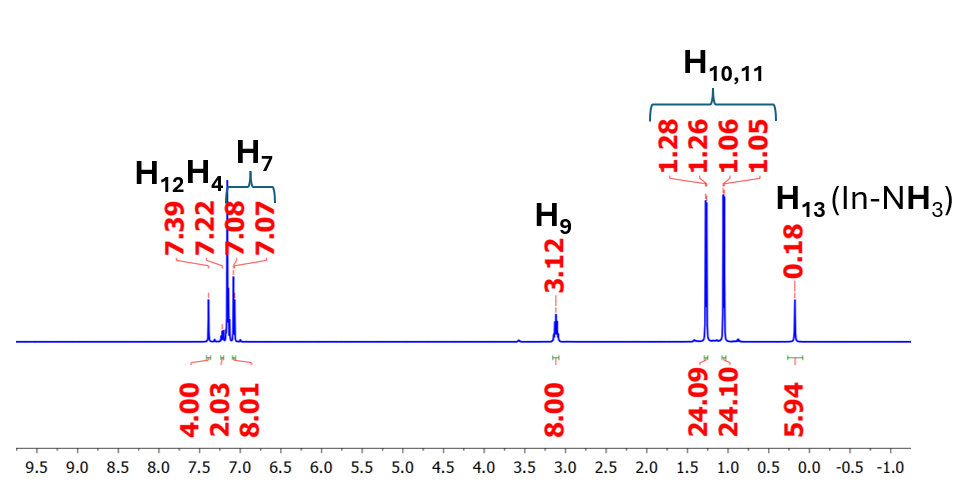


**Figure S21.** ^1^H NMR (298 K, C_6_D_6_, 500 MHz) spectrum of **5**.


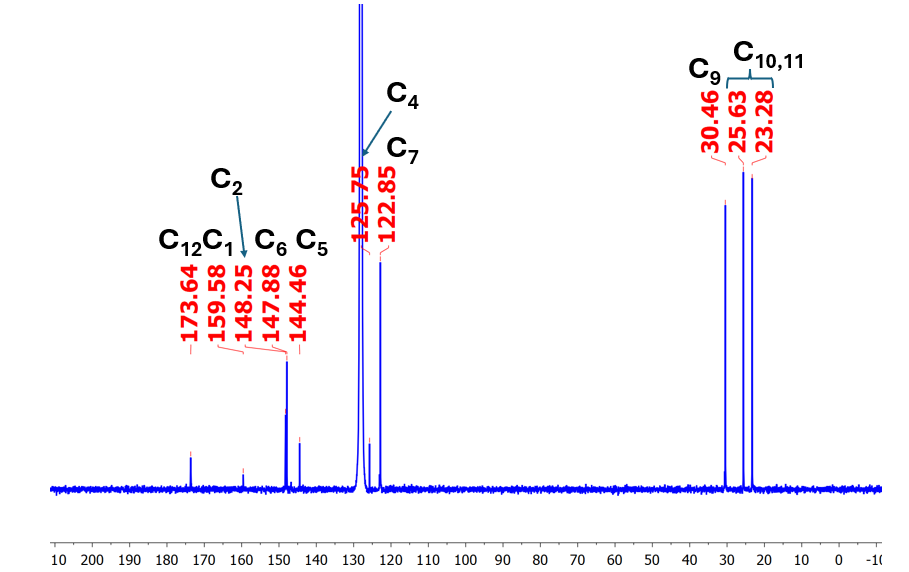


**Figure S22.** ^13^C{^1^H} NMR (298 K, C_6_D_6_, 500 MHz) spectrum of **5**.


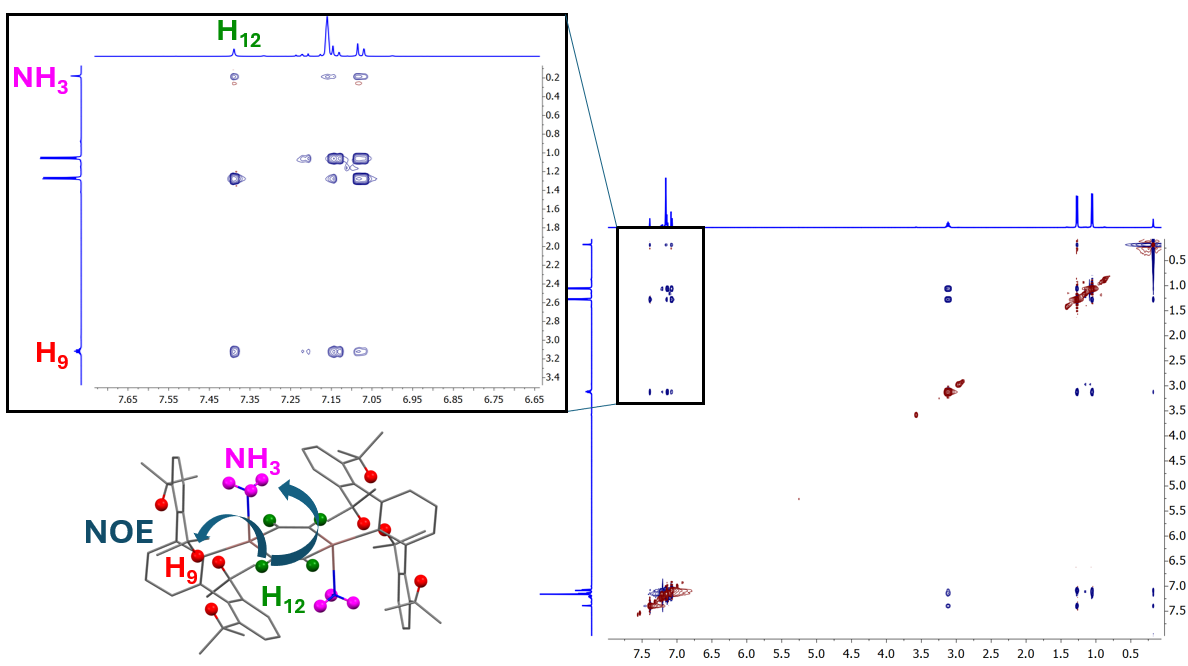


**Figure S23.** Selected region of the ^1^H–^1^H NOESY (298 K, C_6_D_6_, mixing time of 800 ms) spectrum of **5**. Cross-peaks observed between the H_12_ and H_13_ (NH_3_) and H_9_ arise from intramolecular cross‐relaxation of protons that are close to each other in space, confirming the presence of the NH_3_-TerIn-C_2_H_2_ linkage in solution.

## NMR spectra of compound **5_D_**

**Figure S24.** Compound **5_D_**, (InTer)_2_(C_2_H_2_)_2_(ND_3_)_2_, with the atom labelling used for the assignment of NMR spectra.

**^1^H NMR (500 MHz, C_6_D_6_):** δ (ppm) 7.39 (s, 4H, H_12_), 7.22 (t, ^3^*J*_H–H_ = 7.4 Hz, 2H, H_4_), 7.18-7.12 (m, 8H, H_3_+H_8_, overlapped with benzene resonance), 7.07 (d, ^3^*J*_H–H_ = 7.7 Hz, 8H, H_7_), 3.12 (sept, ^3^*J*_H–H_ = 6.9 Hz, 8H, H_9_, CH(CH_3_)_2_), 1.27 (d, ^3^*J*_H–H_ = 6.9 Hz, 24H, H_10_ or H_11_, CH(CH_3_)_2_), 1.04 (d, ^3^*J*_H–H_ = 6.9 Hz, 24H, H_10_ or H_11_, CH(CH_3_)_2_), 0.15 (residual H_13_, In-NHD_2_).

**^2^H{^1^H} NMR (61 MHz, C_6_H_6_):** δ (ppm) 0.10 (s; In-ND_3_).

**^1^H NMR (500 MHz, Tol-d_8_):** δ (ppm) 7.28 (s, 4H, H_12_), 7.20 (t, ^3^*J*_H–H_ = 7.4 Hz, 2H, H_4_), 7.14 (t, ^3^*J*_H–H_ = 7.6 Hz, 4H, H_8_), 7.08 (d, ^3^*J*_H–H_ = 7.4 Hz, 4H, H_3_, overlapped with benzene resonance), 7.04 (d, ^3^*J*_H–H_ = 7.6 Hz, 8H, H_7_), 3.07 (sept, ^3^*J*_H–H_ = 6.9 Hz, 8H, H_9_, CH(CH_3_)_2_), 1.23 (d, ^3^*J*_H–H_ = 6.9 Hz, 24H, H_10_ or H_11_, CH(CH_3_)_2_), 1.04 (d, ^3^*J*_H–H_ = 6.9 Hz, 24H, H_10_ or H_11_, CH(CH_3_)_2_), 0.09 (residual H_13_, In-NHD_2_).

**^2^H{^1^H} NMR (92 MHz, Tol):** δ (ppm) 0.05 (s; In-ND_3_).


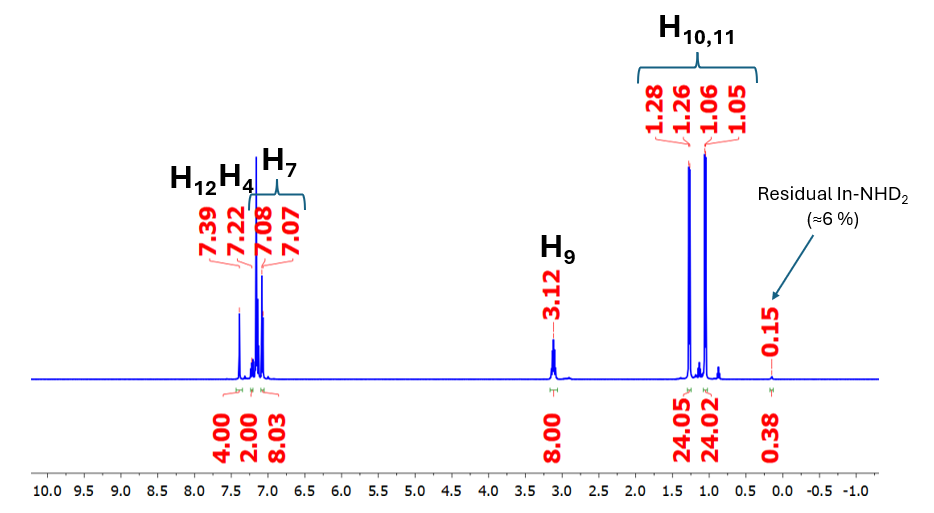


**Figure S25.** ^1^H NMR (298 K, C_6_D_6_, 500 MHz) spectrum of **5_D_**.


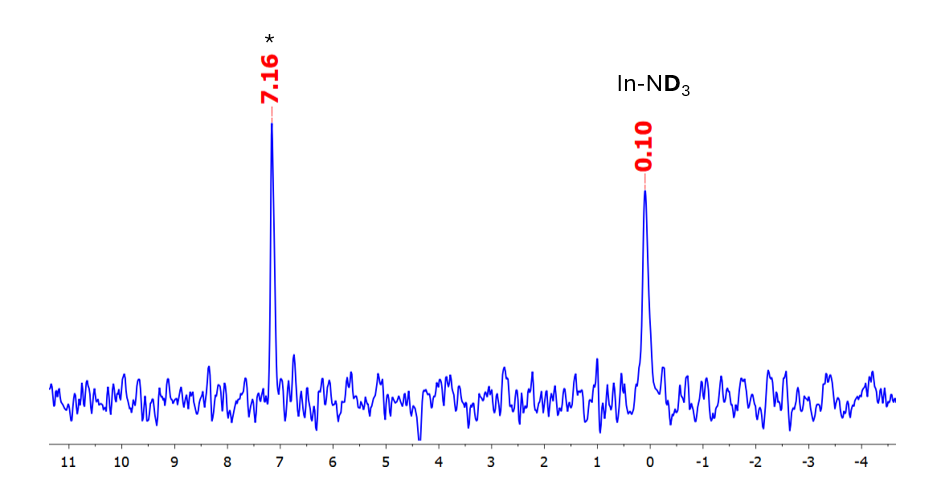


**Figure S26.** ^2^H{^1^H} NMR (298 K, C_6_H_6_, 61 MHz) spectrum of **5_D_**. *Signal corresponding to naturally abundant deuterated benzene at 7.16 ppm.


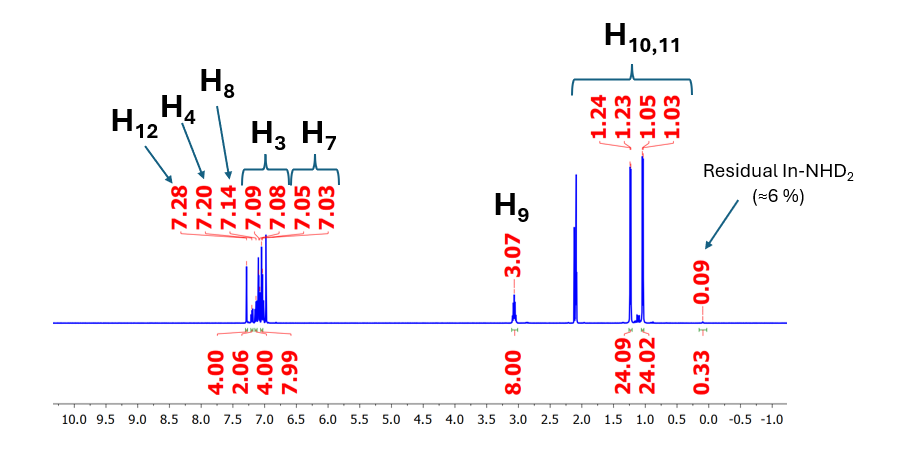


**Figure S27.** ^1^H NMR (298 K, Tol-d_8_, 500 MHz) spectrum of **5_D_**.


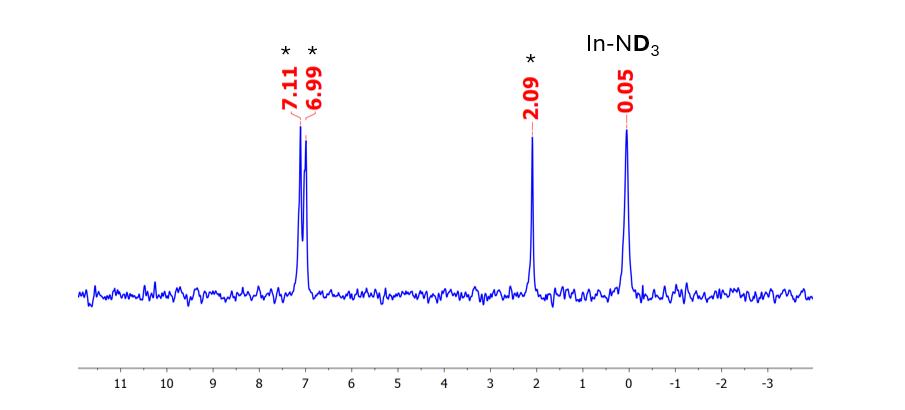


**Figure S28.** ^2^H{^1^H} NMR (298 K, toluene, 92 MHz) spectrum of **5_D_**. *Signals corresponding to naturally abundant deuterated toluene at 7.23–6.92 and 2.09 ppm.

## NMR spectra of compound **6**

**Figure S29.** Compound **6**, (GaTer)_2_(C_2_H_2_)_2_(NH_3_)_2_, with the atom labelling used for the assignment of NMR spectra.

**^1^H NMR (500 MHz, C_6_D_6_):** δ (ppm) 7.30 (s, 4H, H_12_), 7.19 (t, ^3^*J*_H–H_ = 7.7 Hz, 2H, H_4_), 7.13 (t, ^3^*J*_H–H_ = 7.4 Hz, 8H, H_8_), 7.05 (d, ^3^*J*_H–H_ = 7.7 Hz, 4H, H_3_), 7.03 (d, ^3^*J*_H–H_ = 7.4 Hz, 8H, H_7_), 3.13 (sept, ^3^*J*_H–H_ = 6.9 Hz, 8H, H_9_, CH(CH_3_)_2_), 1.28 (d, ^3^*J*_H–H_ = 6.9 Hz, 24H, H_10_ or H_11_, CH(CH_3_)_2_), 1.03 (d, ^3^*J*_H–H_ = 6.9 Hz, 24H, H_10_ or H_11_, CH(CH_3_)_2_), 0.31 (s, 6H, H_13_, Ga-NH_3_).

**^13^C{^1^H} NMR (125 MHz, C_6_D_6_):** δ (ppm) 164.4 (s, C_12_), 152.5 (s, C_1_), 148.0 (s, C_6_), 147.0 (s, C_2_), 144.4 (s, C_5_), 128.6 (s, C_8_), 127.6 (s, C_3_), 125.3 (s, C_4_), 122.6 (s, C_7_), 30.4 (s, C_9_), 25.6 (s, C_10_ or C_11_, CH(CH_3_)_2_), 23.1 (s, C_10_ or C_11_, CH(CH_3_)_2_).


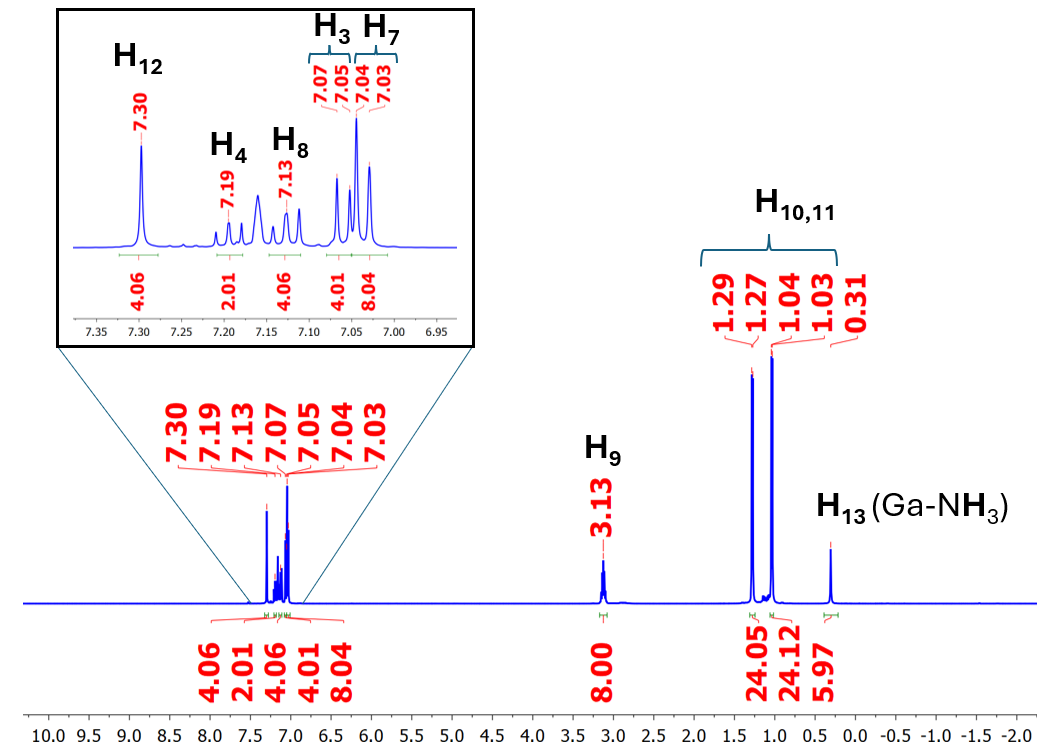


**Figure S30.** ^1^H NMR (298 K, C_6_D_6_, 500 MHz) spectrum of **6**.


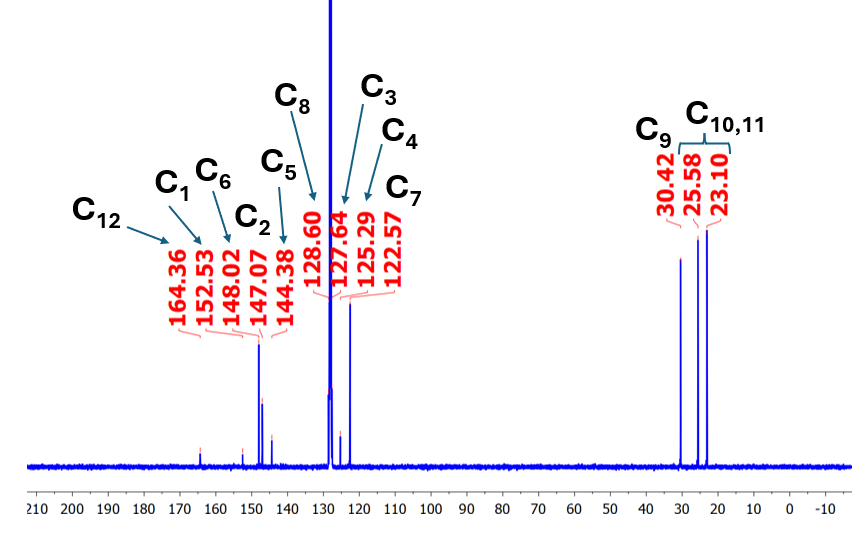


**Figure S31.** ^13^C{^1^H} NMR (298 K, C_6_D_6_, 500 MHz) spectrum of **6**.


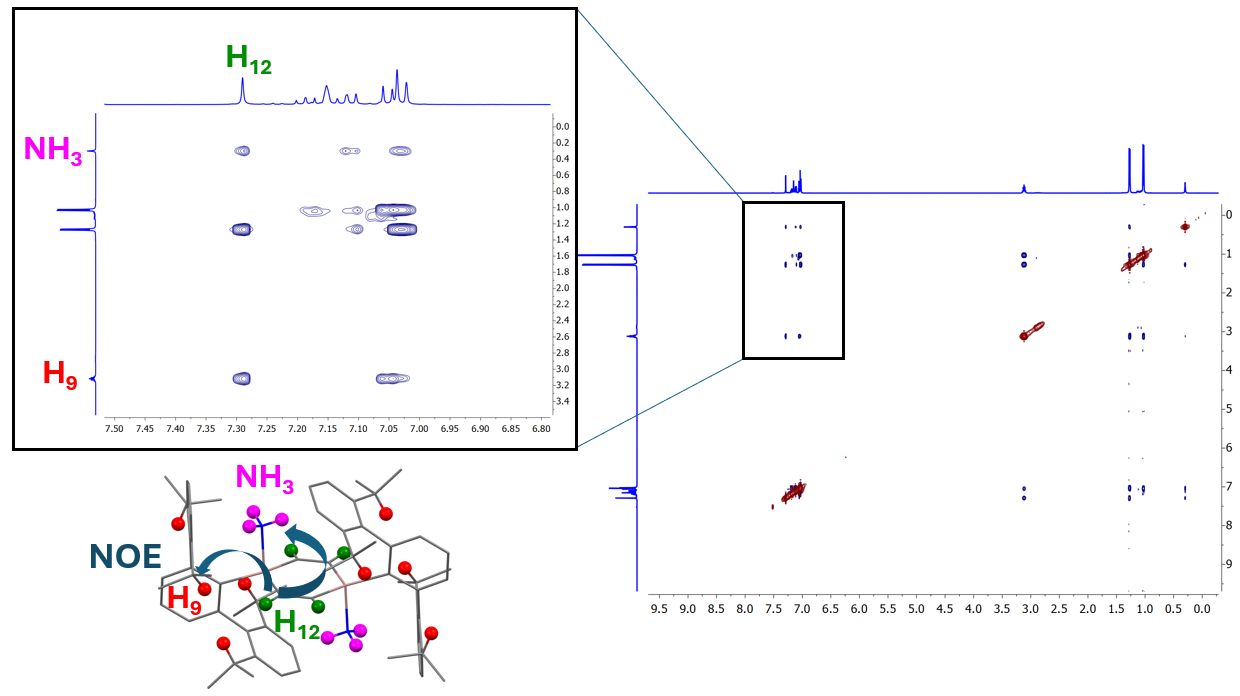


**Figure S32.** Selected region of the ^1^H–^1^H NOESY (298 K, C_6_D_6_, mixing time of 800 ms) spectrum of **6**. Cross-peaks observed between the H_12_ and H_13_ (NH_3_) and H_9_ arise from intramolecular cross‐relaxation of protons that are close to each other in space, confirming the presence of the NH_3_-TerGa-C_2_H_2_ linkage in solution.

## NMR spectra of compound **7**

**Figure S33.** Compound **7**, (InTer)_2_(C_2_H_2_)(µ-NH_2_)_2_, with the atom labelling used for the assignment of NMR spectra.

**^1^H NMR (500 MHz, C_6_D_6_):** δ (ppm) 7.53 (s, 2H, H_12_), 7.26-7.19 (m, 10H, H_3_+H_4_+H_8_), 7.10 (d, ^3^*J*_H–H_ = 7.7 Hz, 8H, H_7_), 2.96 (sept, ^3^*J*_H–H_ = 6.9 Hz, 8H, H_9_, CH(CH_3_)_2_), 1.15 (d, ^3^*J*_H–H_ = 6.9 Hz, 24H, H_10_ or H_11_, CH(CH_3_)_2_), 1.08 (d, ^3^*J*_H–H_ = 6.9 Hz, 24H, H_10_ or H_11_, CH(CH_3_)_2_), -0.96 (d, 2H, ^2^*J*_H–H_ = 9.6 Hz, H_a_, In-NH_2_-In), -1.86 (d, 2H, ^2^*J*_H–H_ = 9.6 Hz, H_b_, In-NH_2_-In).

**^13^C{^1^H} NMR (125 MHz, C_6_D_6_):** δ (ppm) 167.7 (s, C_12_), 155.6 (s, C_1_), 148.8 (s, C_2_), 146.8 (s, C_6_), 143.6 (s, C_5_), C_3_ or C_8_ overlapped with benzene resonance, 127.4 (s, C_3_ or C_8_), 127.2 (s, C_4_), 123.1 (s, C_7_), 30.6 (s, C_9_), 25.6 (s, C_10_ or C_11_, CH(CH_3_)_2_), 23.3 (s, C_10_ or C_11_, CH(CH_3_)_2_).


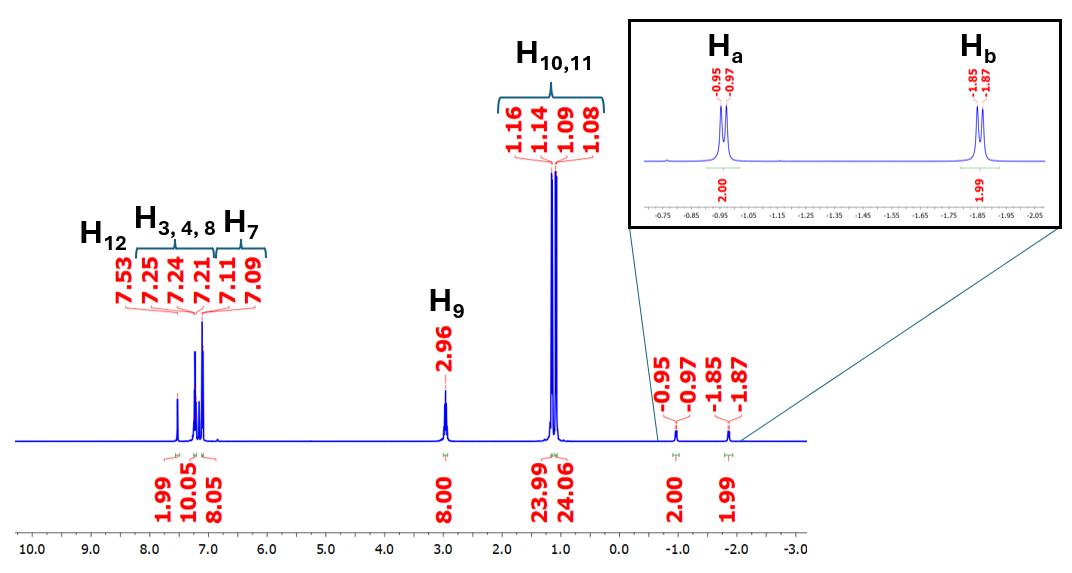


**Figure S34.** ^1^H NMR (298 K, C_6_D_6_, 500 MHz) spectrum of **7**.


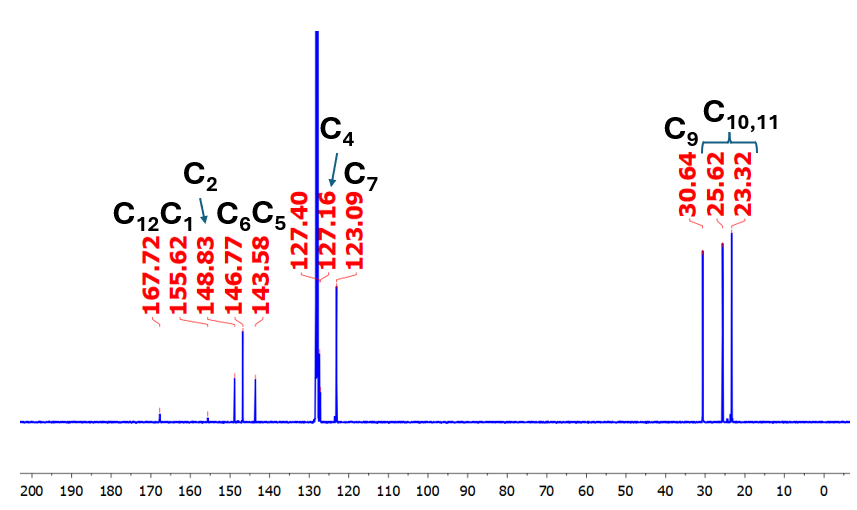


**Figure S35.** ^13^C{^1^H} NMR (298 K, C_6_D_6_, 500 MHz) spectrum of **7**.


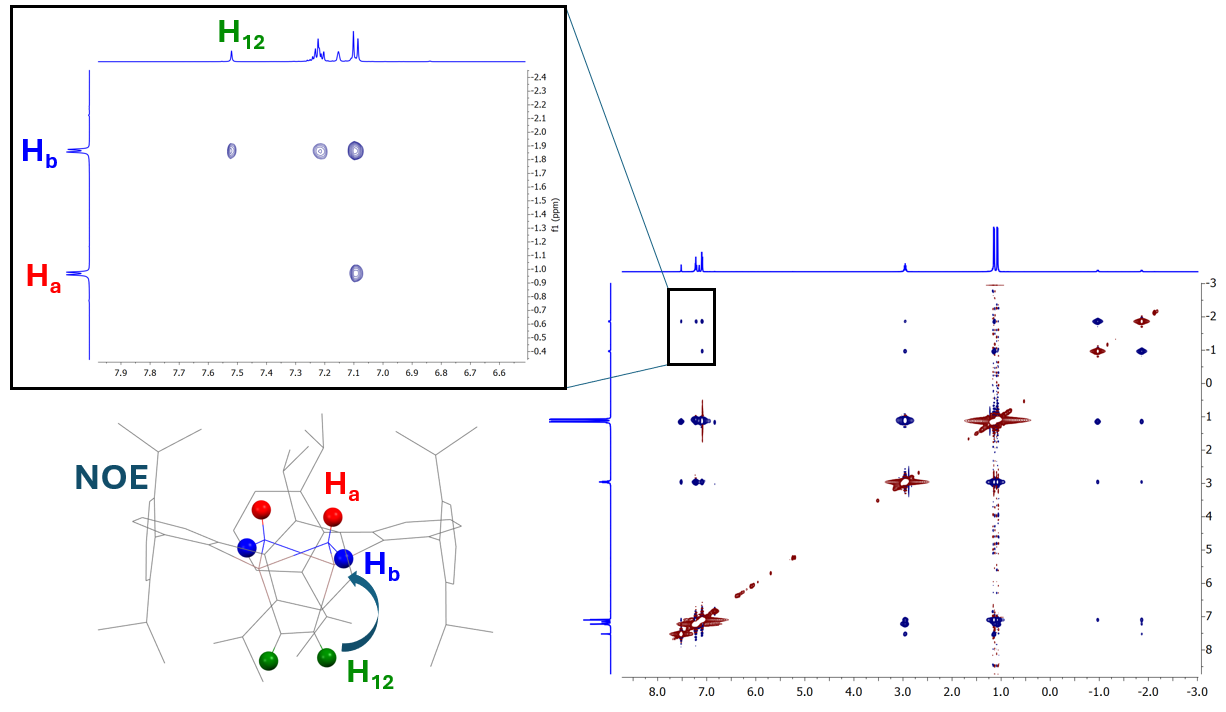


**Figure S36.** Selected region of the ^1^H–^1^H NOESY (298 K, C_6_D_6_, mixing time of 800 ms) spectrum of **7**. Cross-peak observed between the H_12_ and H_b_ (NH_2_) arise from intramolecular cross‐relaxation of protons that are close to each other in space.

## NMR spectra of compound **7_D_**

**Figure S37.** Compound **7_D_**, (InTer)_2_(C_2_H_2_)(µ-ND_2_)_2_, with the atom labelling used for the assignment of NMR spectra.

**^1^H NMR (500 MHz, C_6_D_6_):** δ (ppm) 7.53 (s, 2H, H_12_), 7.26-7.19 (m, 10H, H_3_+H_4_+H_8_), 7.10 (d, ^3^*J*_H–H_ = 7.7 Hz, 8H, H_7_), 2.97 (sept, ^3^*J*_H–H_ = 6.9 Hz, 8H, H_9_, CH(CH_3_)_2_), 1.15 (d, ^3^*J*_H–H_ = 6.9 Hz, 24H, H_10_ or H_11_, CH(CH_3_)_2_), 1.08 (d, ^3^*J*_H–H_ = 6.9 Hz, 24H, H_10_ or H_11_, CH(CH_3_)_2_), -0.98 (br, residual H_a_, In-NHD-In), -1.88 (br, residual H_b_, In-NHD-In).

.

**^1^H NMR (500 MHz, Tol-d_8_):** δ (ppm) 7.41 (s, 2H, H_12_), 7.26-7.17 (m, 10H, H_3_+H_4_+H_8_), 7.07 (d, ^3^*J*_H–H_ = 7.7 Hz, 8H, H_7_), 2.92 (sept, ^3^*J*_H–H_ = 6.9 Hz, 8H, H_9_, CH(CH_3_)_2_), 1.12 (d, ^3^*J*_H–H_ = 6.9 Hz, 24H, H_10_ or H_11_, CH(CH_3_)_2_), 1.06 (d, ^3^*J*_H–H_ = 6.9 Hz, 24H, H_10_ or H_11_, CH(CH_3_)_2_), -1.04 (br, residual H_a_, In-NHD-In), -1.96 (br, residual H_b_, In-NHD-In).

**^2^H{^1^H} NMR (92 MHz, Tol):** δ (ppm) -1.05 (br, 2D, D_a_, In-ND_2_-In), -2.01 (br, 2D, D_b_, In-ND_2_-In).


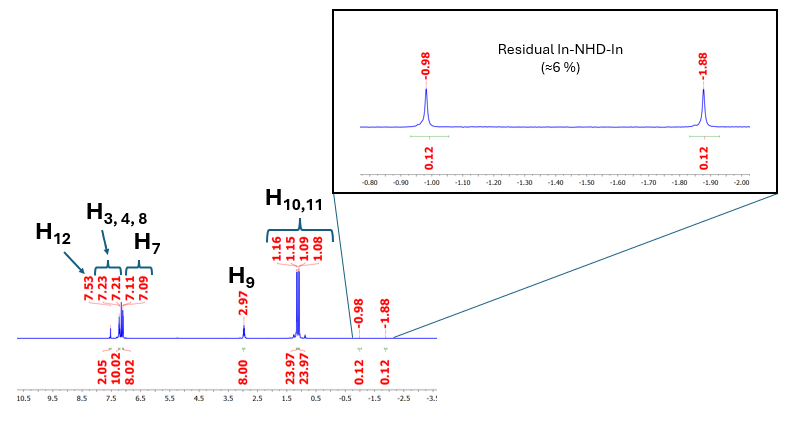


**Figure S38.** ^1^H NMR (298 K, C_6_D_6_, 500 MHz) spectrum of **7_D_**.


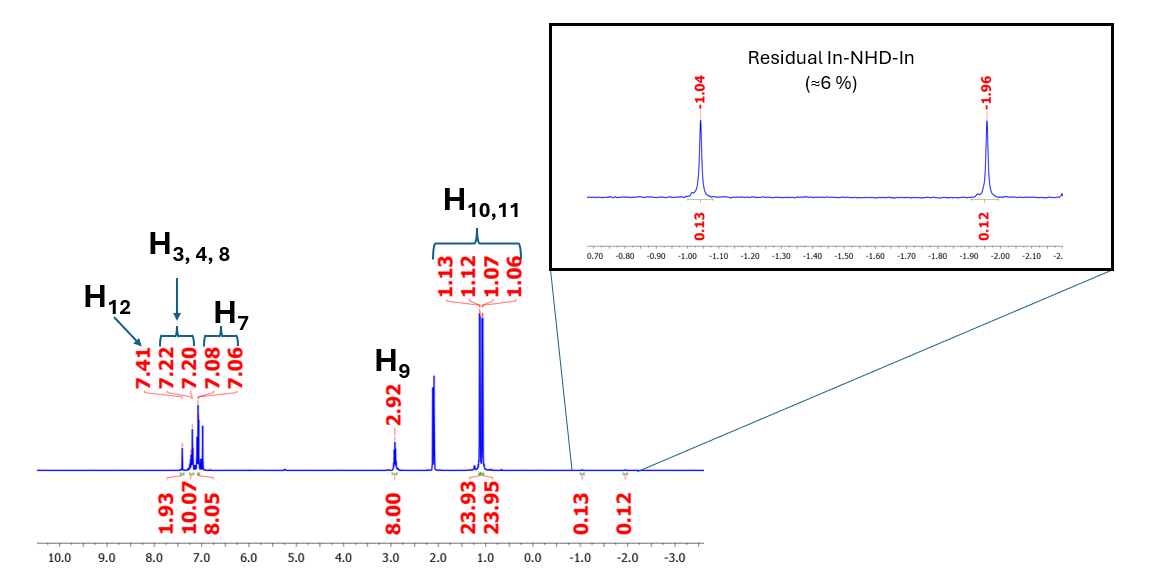


**Figure S39.** ^1^H NMR (298 K, Tol-d_8_, 500 MHz) spectrum of **7_D_**.


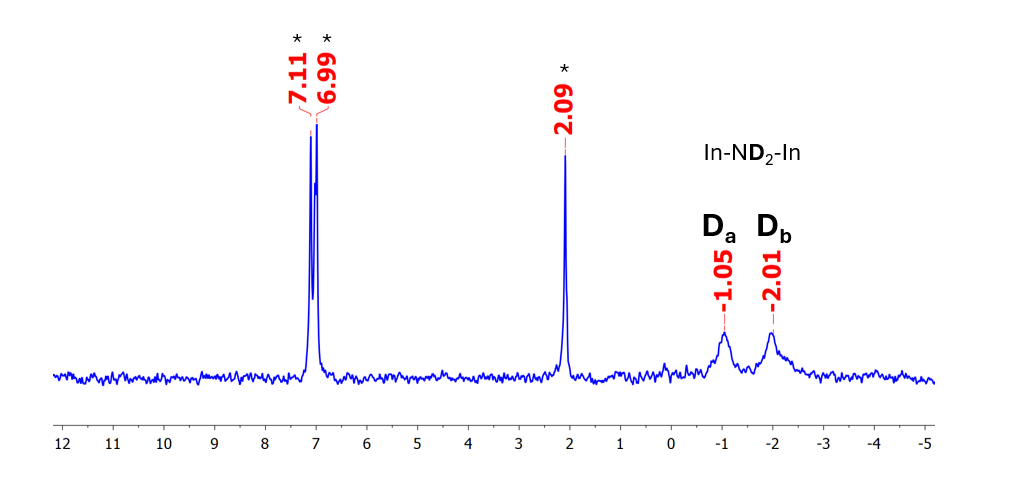


**Figure S40.** ^2^H{^1^H} NMR (298 K, toluene, 92 MHz) spectrum of **7_D_**. *Signals corresponding to naturally abundant deuterated toluene at 7.23-6.92 and 2.09 ppm.

## NMR spectra of compound **8**

**Figure S41.** Compound **8**, (GaTer)_2_(C_2_H_2_)(µ-NH_2_)_2_, with the atom labelling used for the assignment of NMR spectra.

**^1^H NMR (500 MHz, C_6_D_6_):** δ (ppm) 7.42 (s, 2H, H_12_), 7.27-7.16 (m, 10H, H_3_+H_4_+H_8_, overlapped with benzene resonance), 7.07 (d, ^3^*J*_H–H_ = 7.7 Hz, 8H, H_7_), 2.90 (sept, ^3^*J*_H–H_ = 6.9 Hz, 8H, H_9_, CH(CH_3_)_2_), 1.11 (d, ^3^*J*_H–H_ = 6.9 Hz, 24H, H_10_ or H_11_, CH(CH_3_)_2_), 1.06 (d, ^3^*J*_H–H_ = 6.9 Hz, 24H, H_10_ or H_11_, CH(CH_3_)_2_), -0.57 (d, 2H, ^2^*J*_H–H_ = 9.9 Hz, H_a_, Ga-NH_2_-Ga), -1.54 (d, 2H, ^2^*J*_H–H_ = 9.9 Hz, H_b_, Ga-NH_2_-Ga).

**^13^C{^1^H} NMR (125 MHz, C_6_D_6_):** δ (ppm) 163.1 (s, C_12_), 147.8 (s, C_2_), 146.8 (s, C_1_), 146.7 (s, C_6_), 142.4 (s, C_5_), C_3_ and C_8_ overlapped with benzene resonance, 127.1 (s, C_4_), 122.7 (s, C_7_), 30.5 (s, C_9_), 25.7 (s, C_10_ or C_11_, CH(CH_3_)_2_), 23.2 (s, C_10_ or C_11_, CH(CH_3_)_2_).


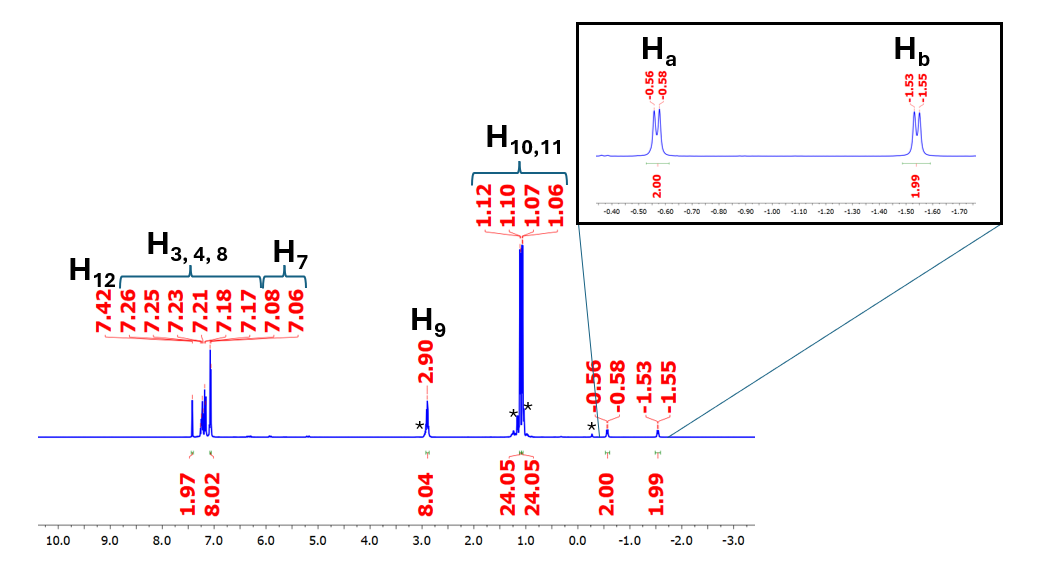


**Figure S42.** ^1^H NMR (298 K, C_6_D_6_, 500 MHz) spectrum of **8**. *Unknown impurity (<15%) with a characteristic resonance at –0.27 ppm. Despite extensive efforts, a pure sample of **8** could not be isolated.


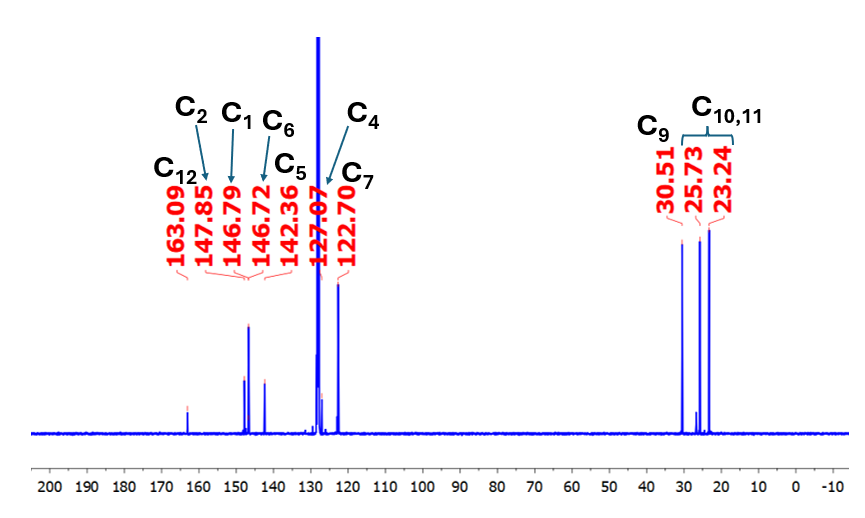


**Figure S43.** ^13^C{^1^H} NMR (298 K, C_6_D_6_, 500 MHz) spectrum of **8**.

# *In situ* NMR Studies


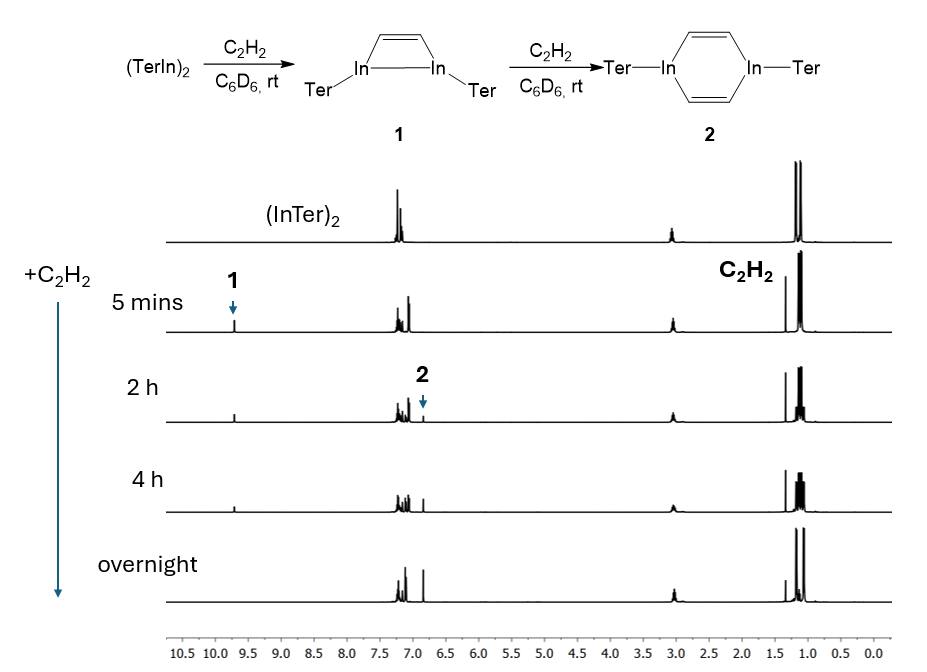


**Figure S44**. ^1^H NMR stacked spectra of the evolution over time of a sample of **1** in C_6_D_6_ solution at room temperature in the presence of acetylene showing the gradual formation of **2**.


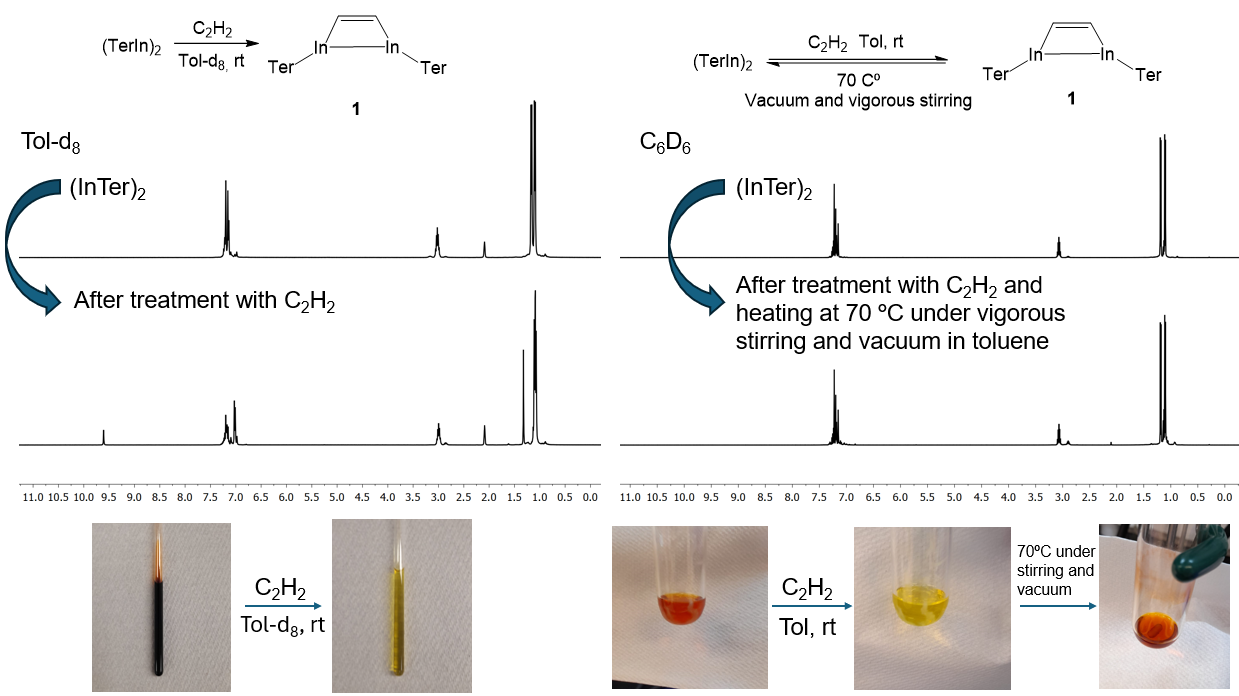


**Figure S45.** Left) ^1^H NMR stacked spectra of the reaction of (InTer)_2_ with acetylene to form **1**. Images of the reaction solution before (red) and after treatment with acetylene (yellow). Right) ^1^H NMR stacked spectra of the reaction of (InTer)_2_ with acetylene and the subsequent retro-cyclization under reduced pressure and vigorous stirring at 70 °C. Images of the reaction solution before (red), after treatment with acetylene (yellow) and after applying heating and vacuum under vigorous stirring (red).


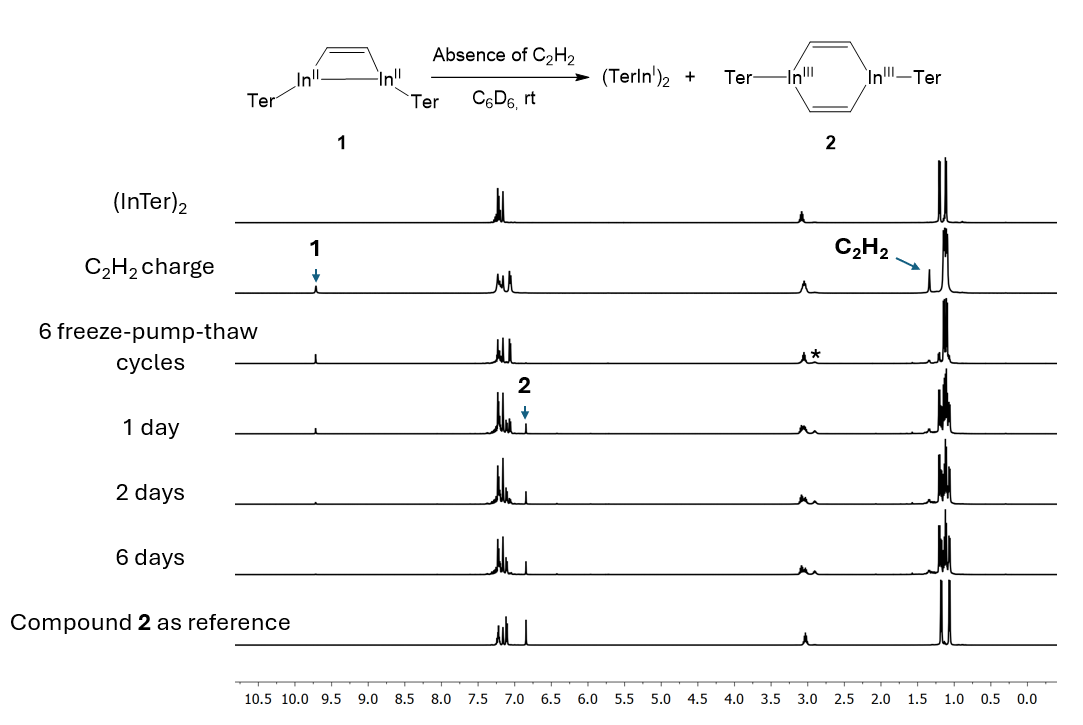


**Figure S46.** ^1^H NMR stacked spectra of the evolution over time of a sample of **1** in C_6_D_6_ solution at room temperature in the absence of acetylene showing the disproportionation to form (InTer)_2_ and **2**. Note: *Residual H-Ter.


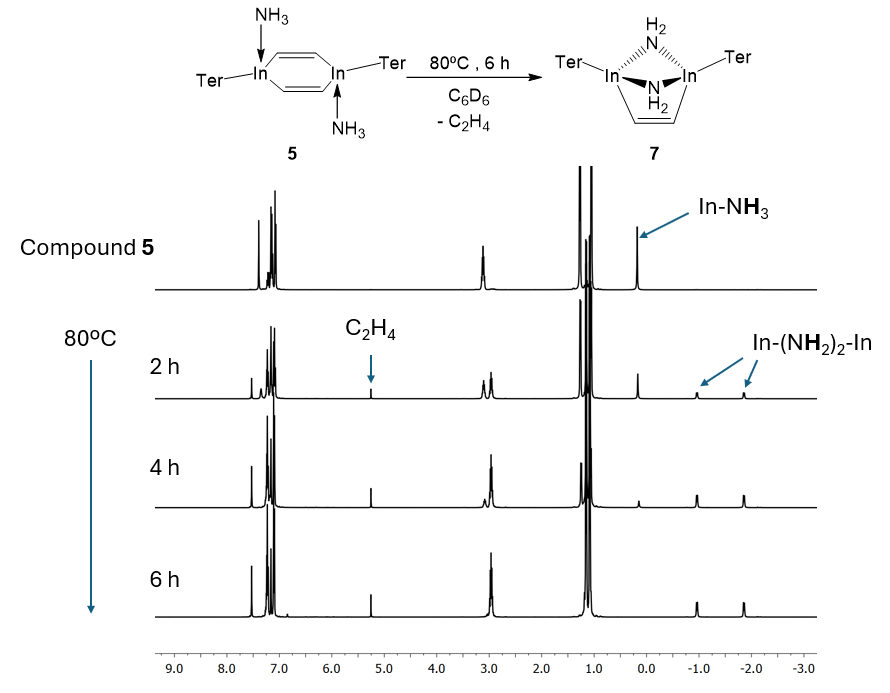


**Figure S47.** ^1^H NMR stacked spectra of the evolution over time of a sample of **5** in C_6_D_6_ solution at 80 ºC, leading to the formation of ethylene and compound **7**.


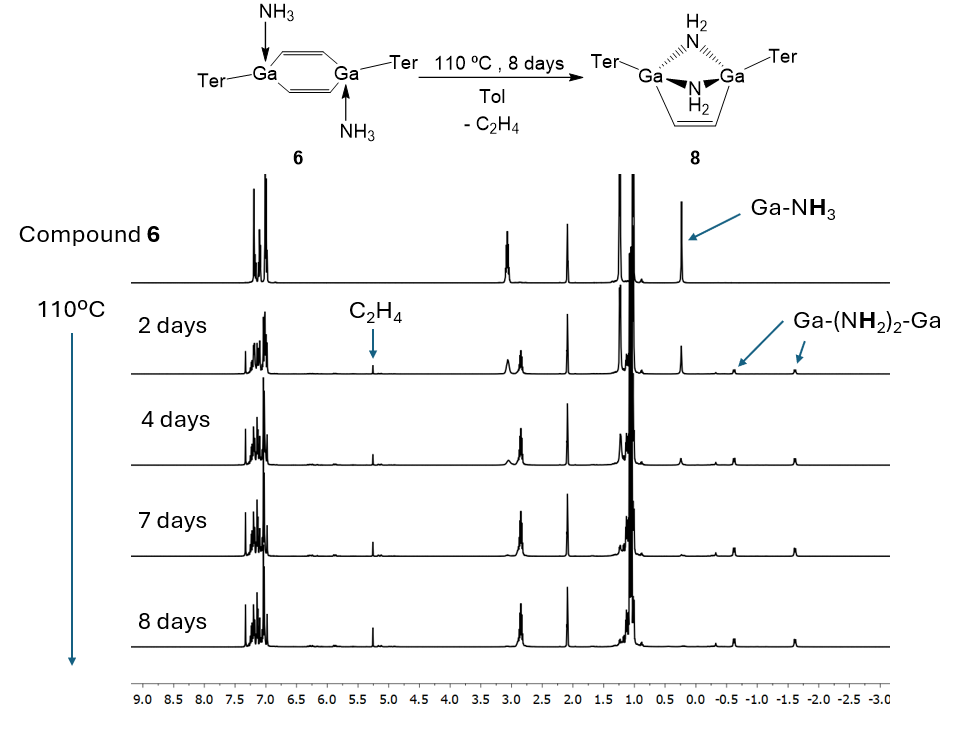


**Figure S48.** ^1^H NMR stacked spectra of the evolution over time of a sample of **6** in Tol-d_8_ solution at 110 ºC, leading to the formation of ethylene and compound **8**.


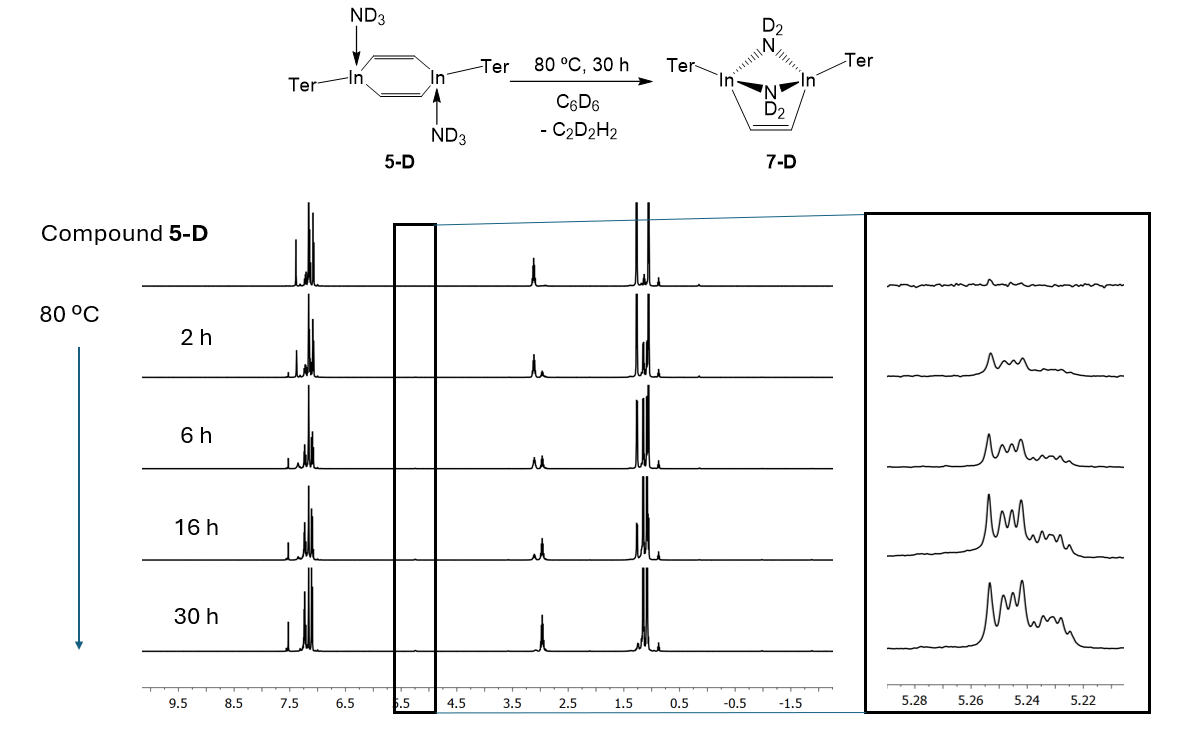


**Figure S49.** ^1^H NMR stacked spectra of the evolution over time of a sample of **5_D_** in C_6_D_6_ solution at 80 ºC, leading to the formation of ethylene and compound **7_D_**.


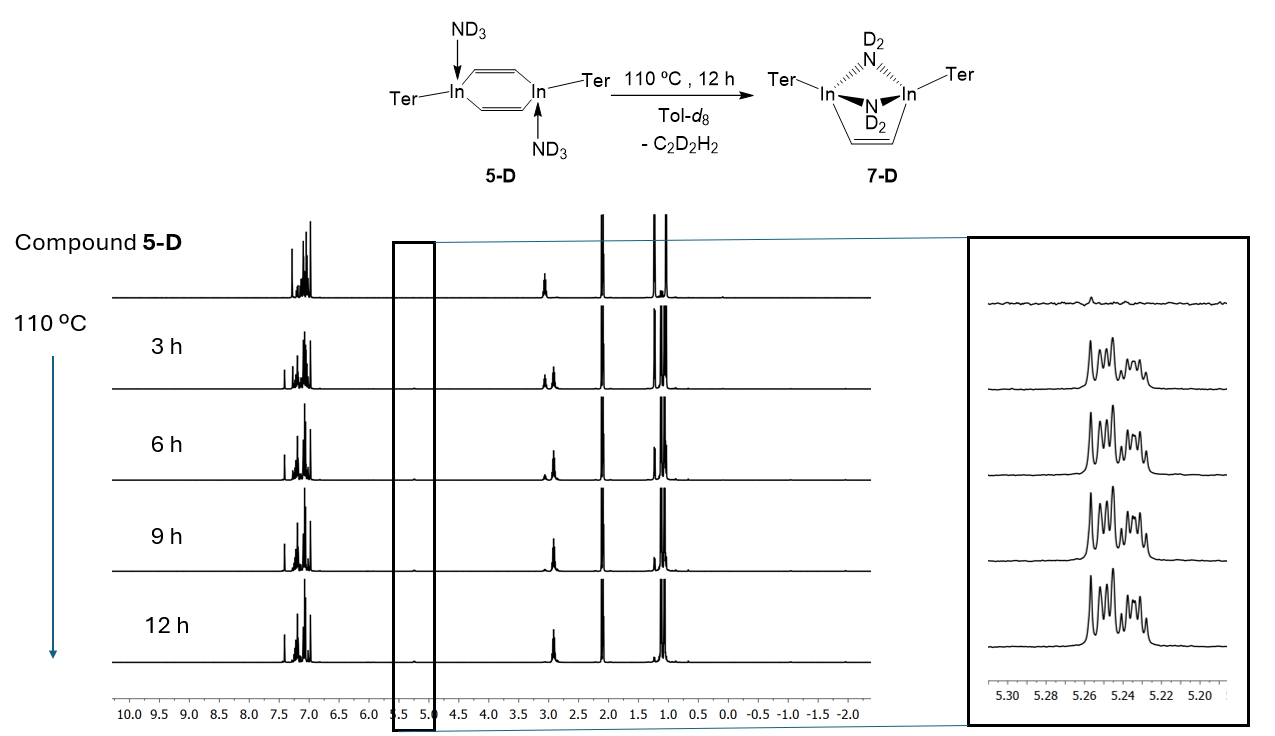


**Figure S50.** ^1^H NMR stacked spectra of the evolution over time of a sample of **5_D_** in Tol-d_8_ solution at 110 ºC, leading to the formation of ethylene and compound **7_D_**.


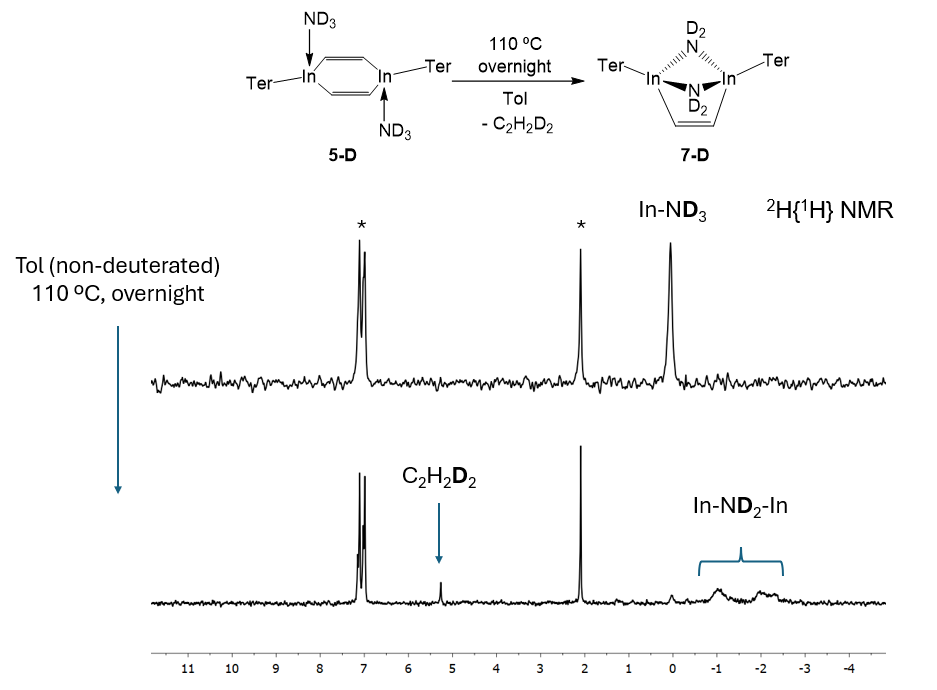


**Figure S51.** ^2^H{^1^H} NMR stacked spectra of the overnight evolution of a sample of **5_D_** in non-deuterated toluene solution at 110 ºC, leading to the formation of ethylene and compound **7_D_**. *Signals corresponding to naturally abundant deuterated toluene at 7.23-6.92 and 2.09 ppm.

# Infrared spectra


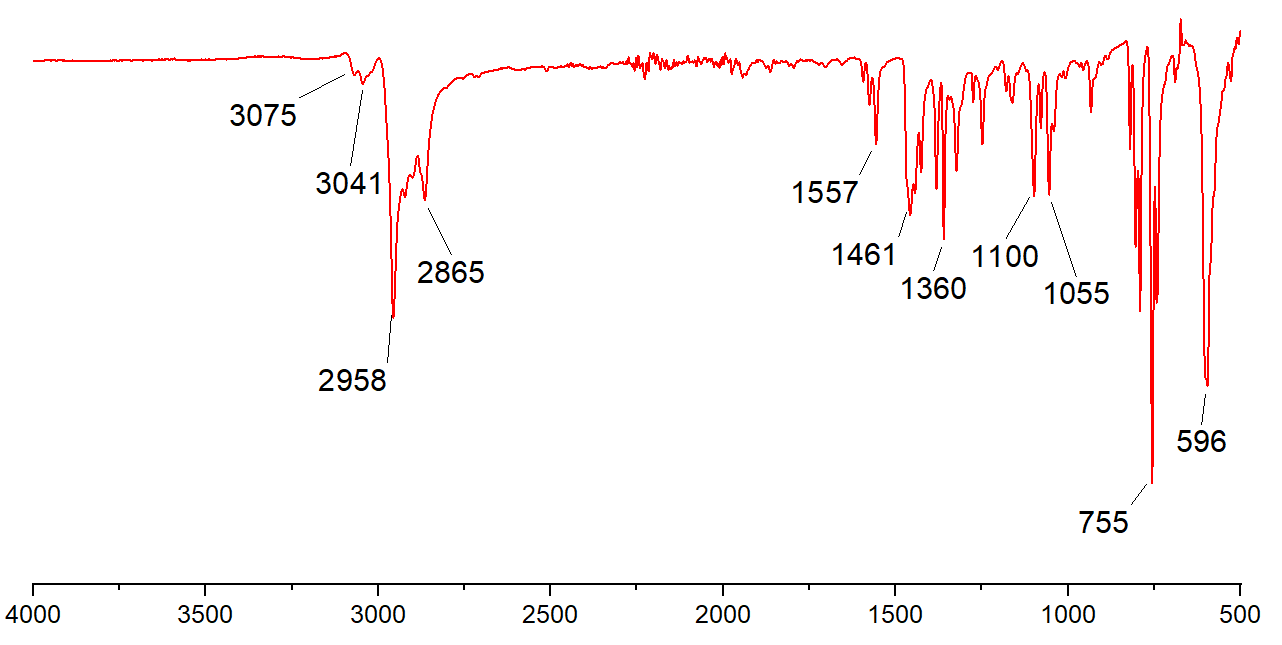


**Figure S52.** IR spectra of (InTer)_2_(C_2_H_2_)_2_ (**2**).


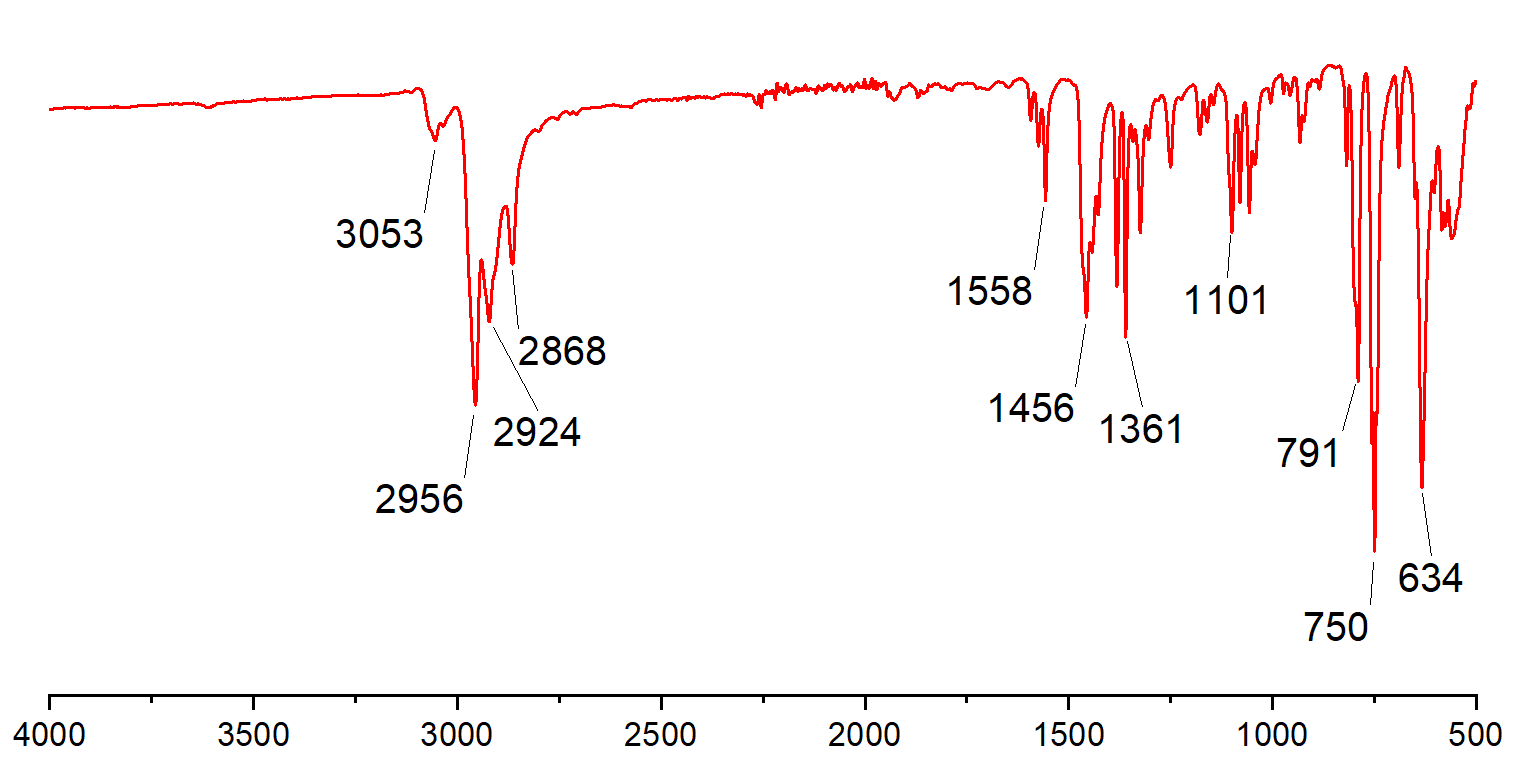


**Figure S53.** IR spectra of (GaTer)_2_(C_2_H_2_)_2_ (**3**).


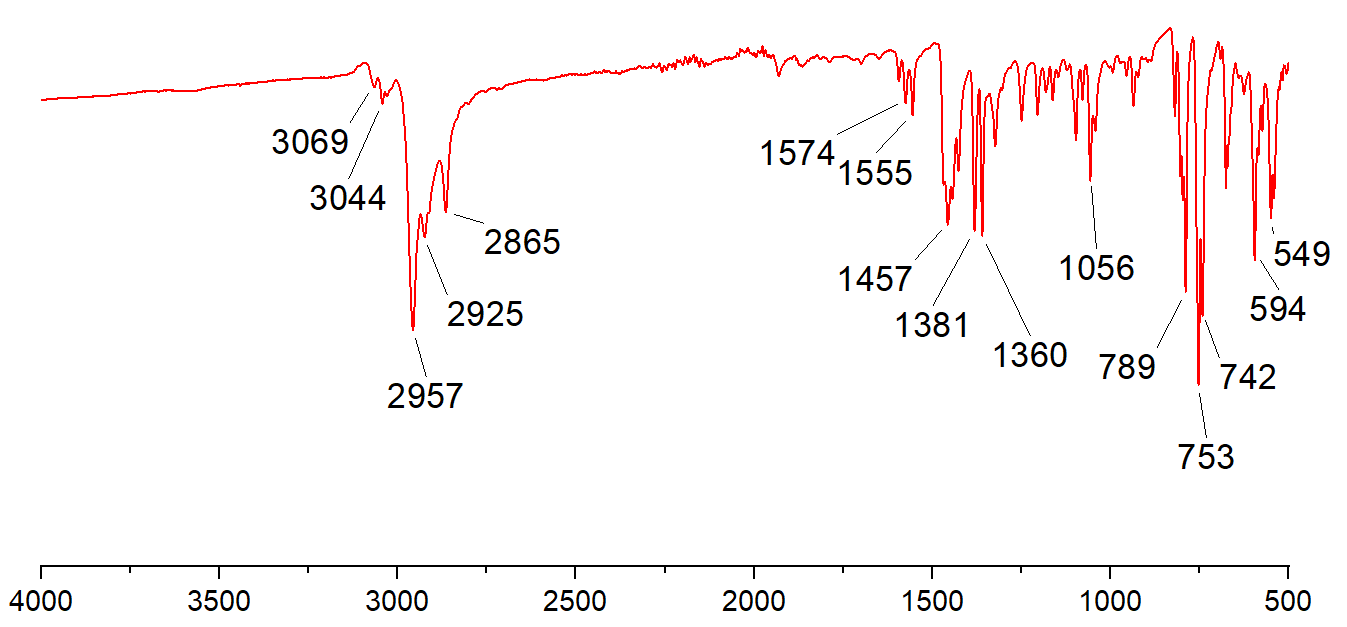


**Figure S54.** IR spectra of (GaTer)_2_(C_2_H_2_) (**4**).


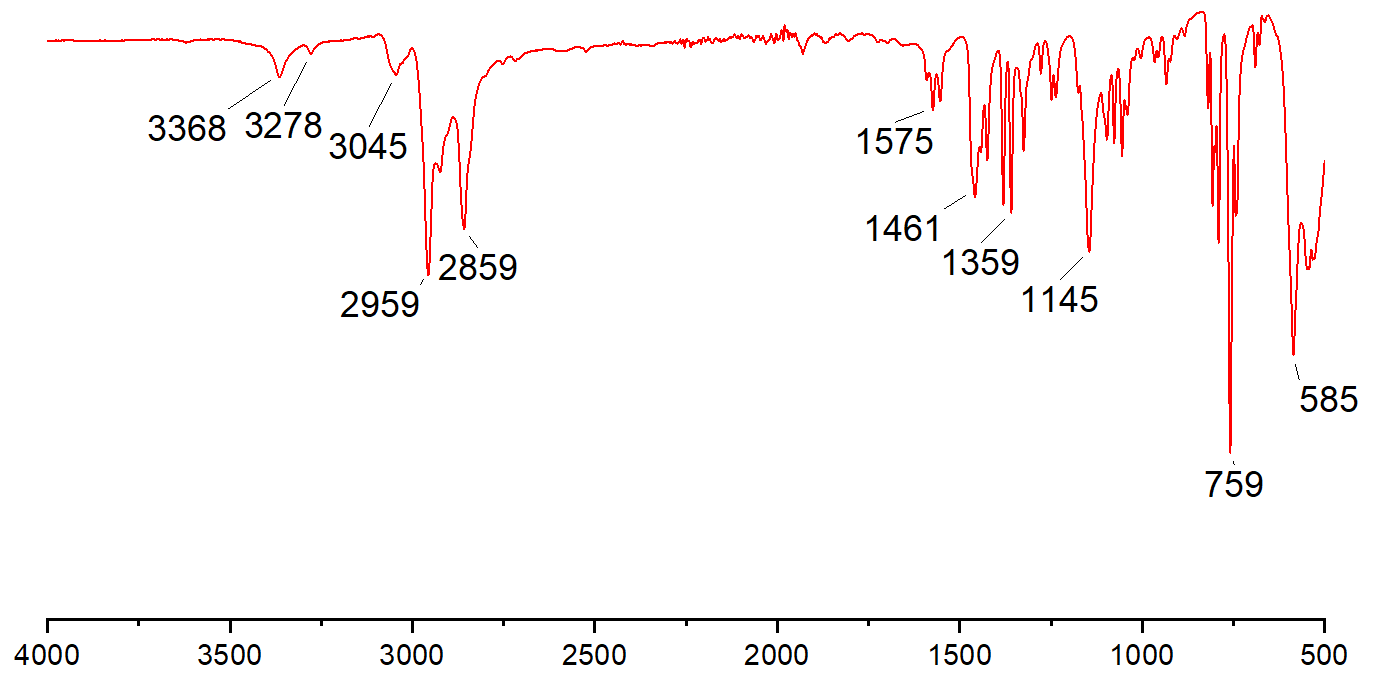


**Figure S55.** IR spectra of (InTer)_2_(C_2_H_2_)_2_(NH_3_)_2_ (**5**).


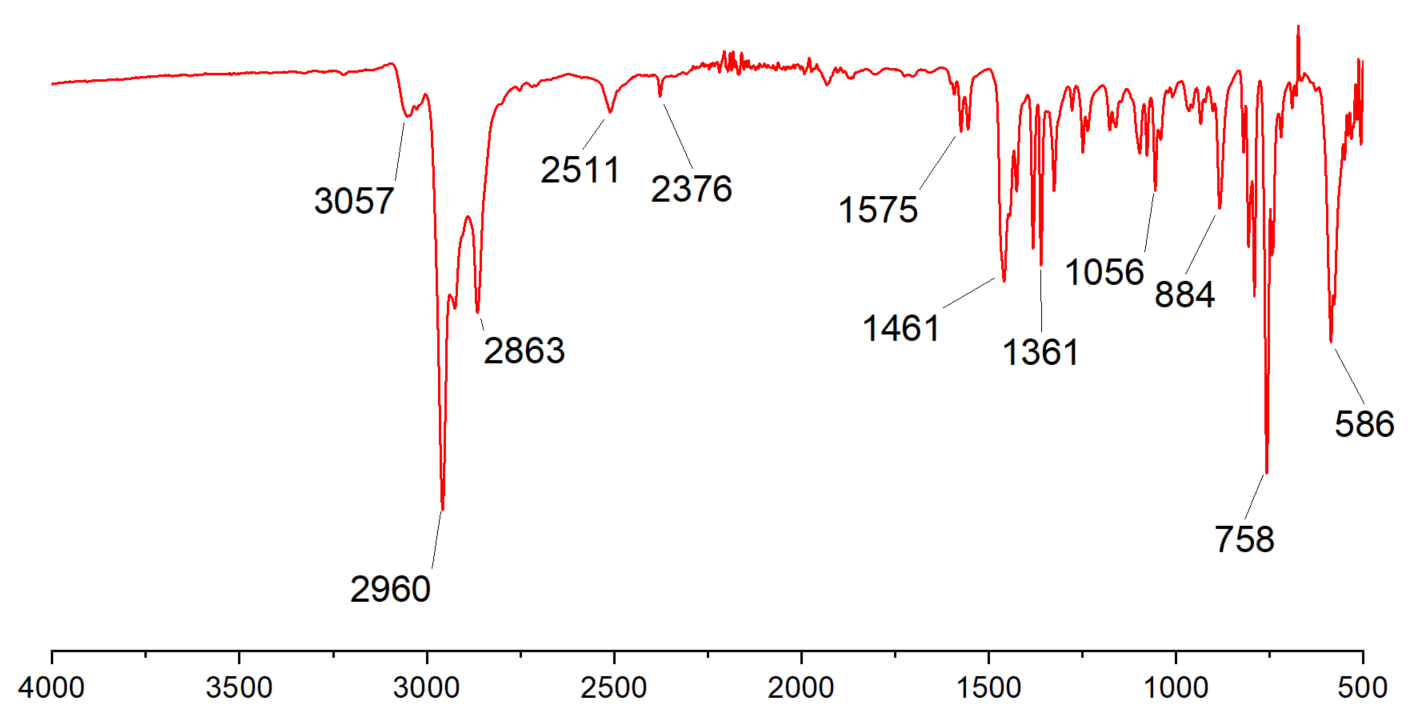


**Figure S56.** IR spectra of (InTer)_2_(C_2_H_2_)_2_(ND_3_)_2_ (**5_D_**).


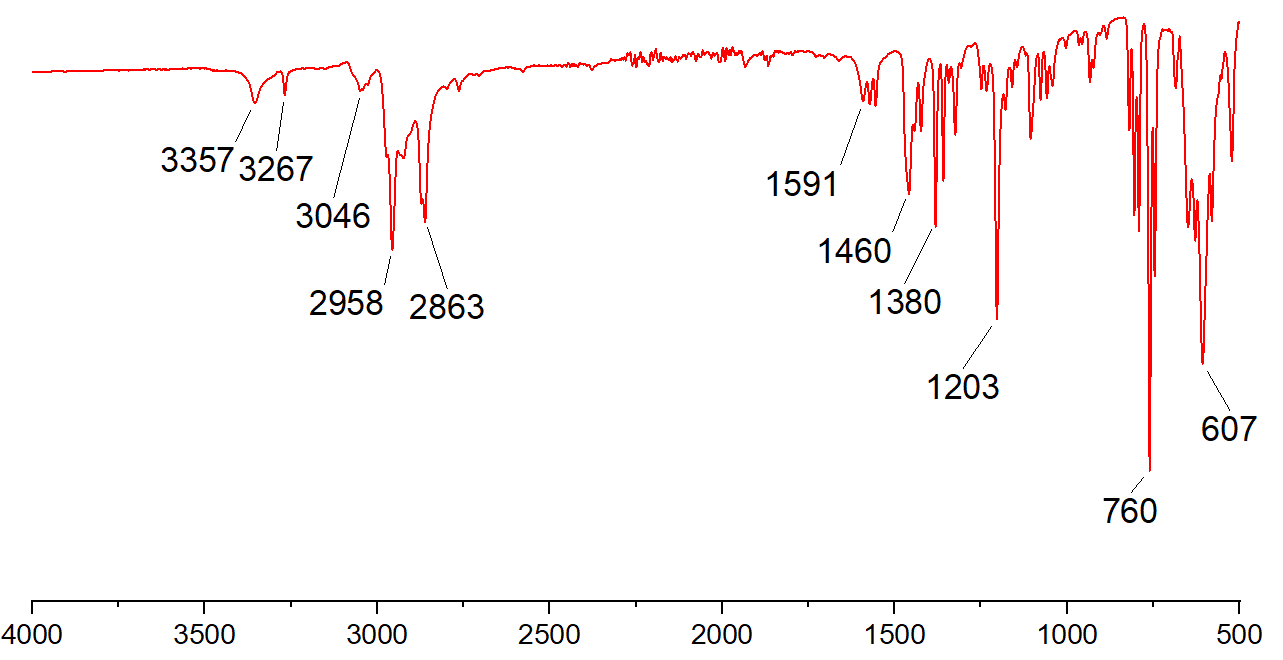


**Figure S57.** IR spectra of (GaTer)_2_(C_2_H_2_)_2_(NH_3_)_2_ (**6**).


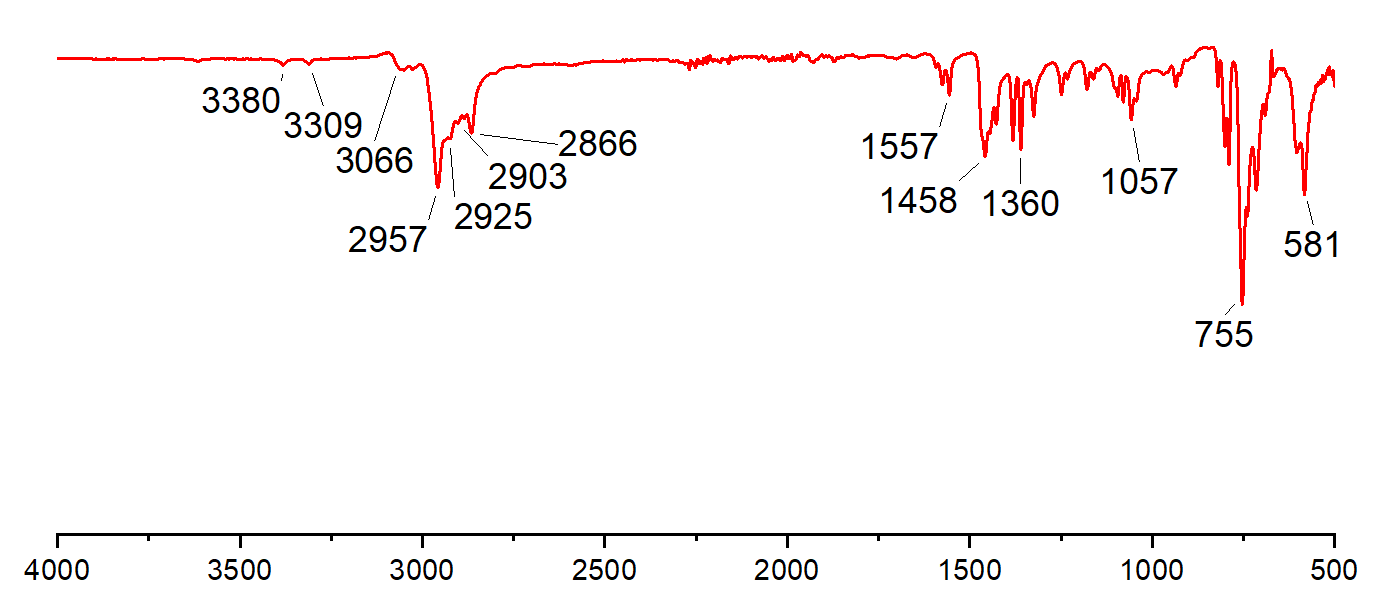


**Figure S58.** IR spectra of (InTer)_2_(C_2_H_2_)(µ-NH_2_)_2_ (**7**).


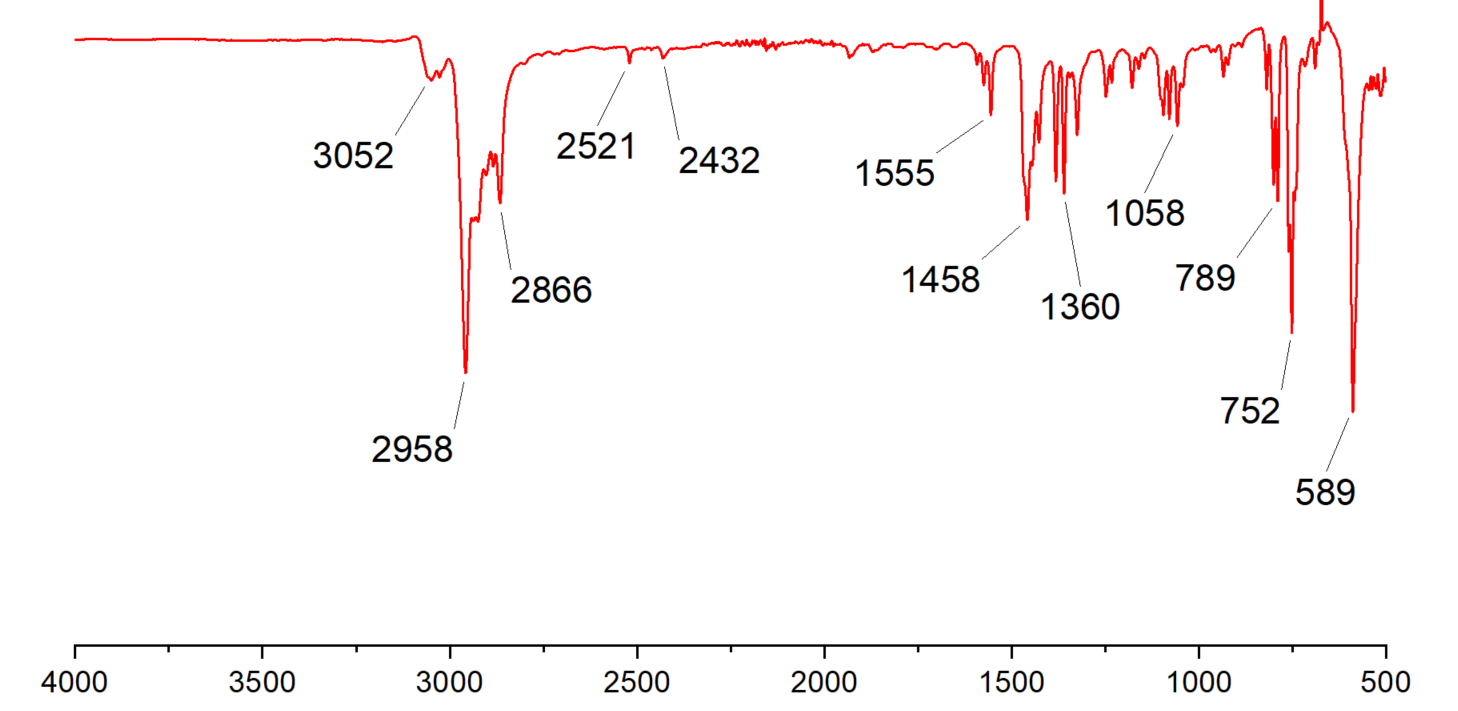


**Figure S59**. IR spectra of (InTer)_2_(C_2_H_2_)(µ-ND_2_)_2_ (**7_D_**).


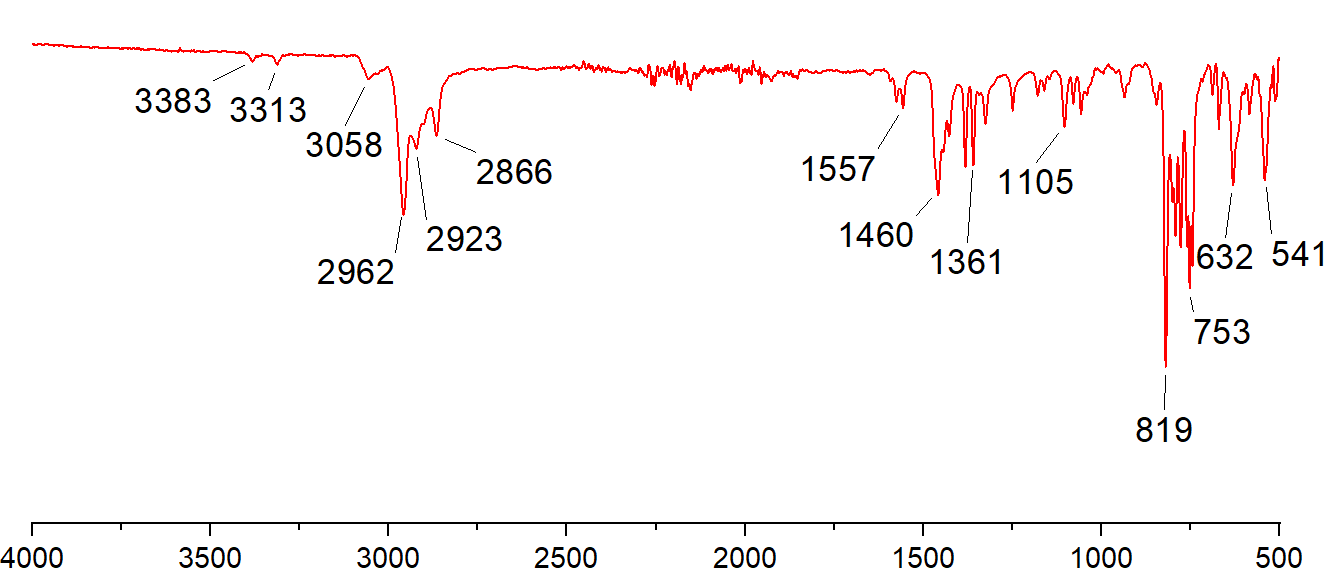


**Figure S60.** IR spectra of (GaTer)_2_(C_2_H_2_)(µ-NH_2_)_2_ (**8**).

# High resolution mass data


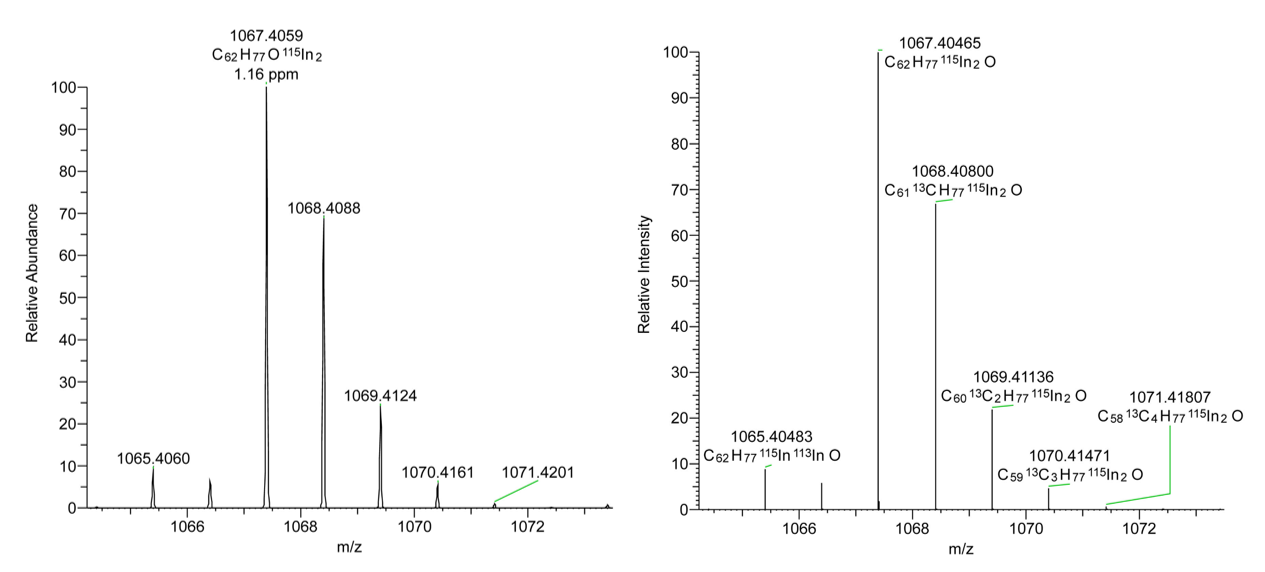
**Figure S61.** HR-MS (APCI-Orbitrap) (positive mode) of (InTer)_2_(C_2_H_2_) (**1**) showing the [M+O+H]^+^ peak at 1067.4059 m/z 1067.4046 (calcd 1067.4046; 1.16 ppm error) (left) and simulation (right).


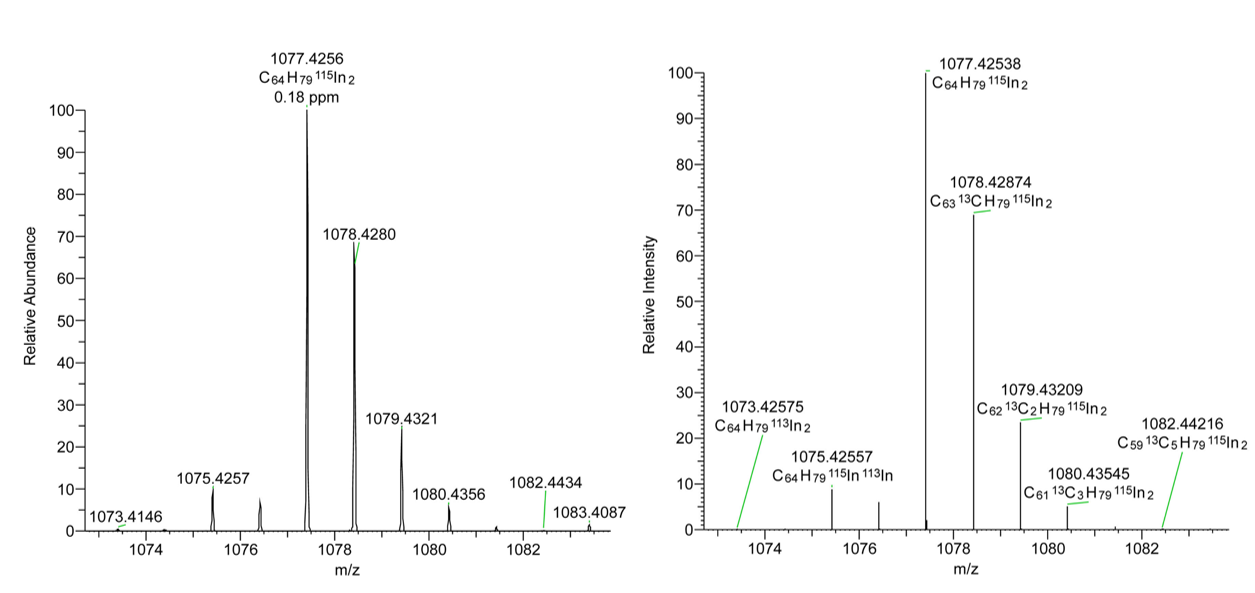


**Figure S62.** HR-MS (APCI- Orbitrap) (positive mode) of (InTer)_2_(C_2_H_2_)_2_ (**2**) showing the [M+H]^+^ peak at m/z 1077.4256 (calcd. 1077.4253; 0.18 ppm error) (left) and simulation (right).


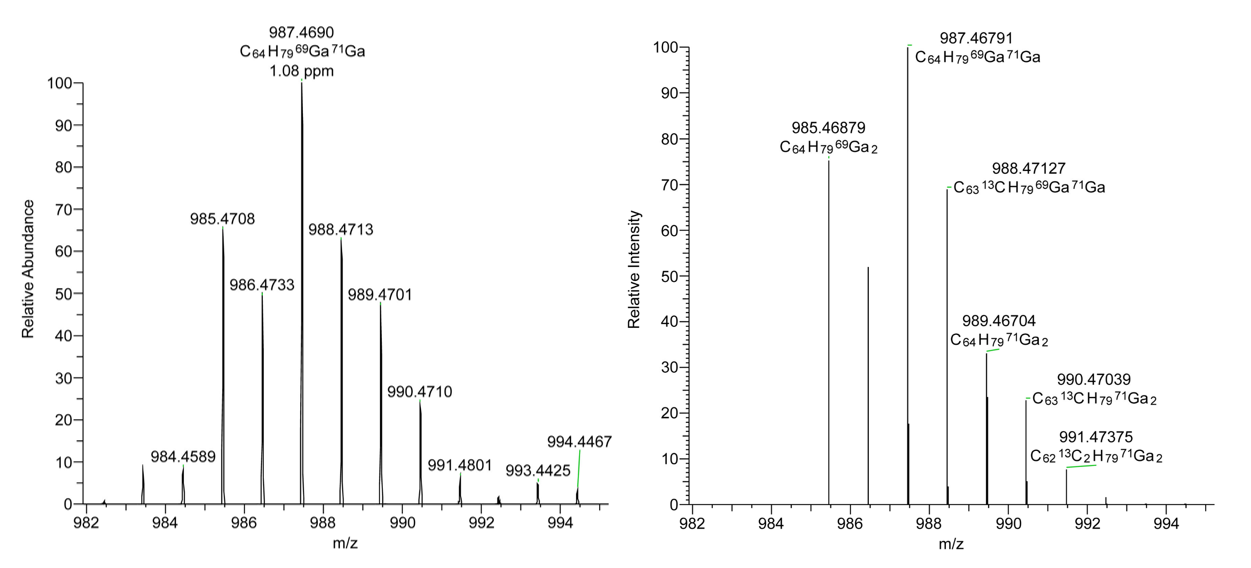


**Figure S63.** HR-MS (APCI- Orbitrap) (positive mode) of (GaTer)_2_(C_2_H_2_)_2_ (**3**) showing the [M+H]^+^ peak at m/z 987.4690 (calcd. 987.4679; 1.08 ppm error) (left) and simulation (right).


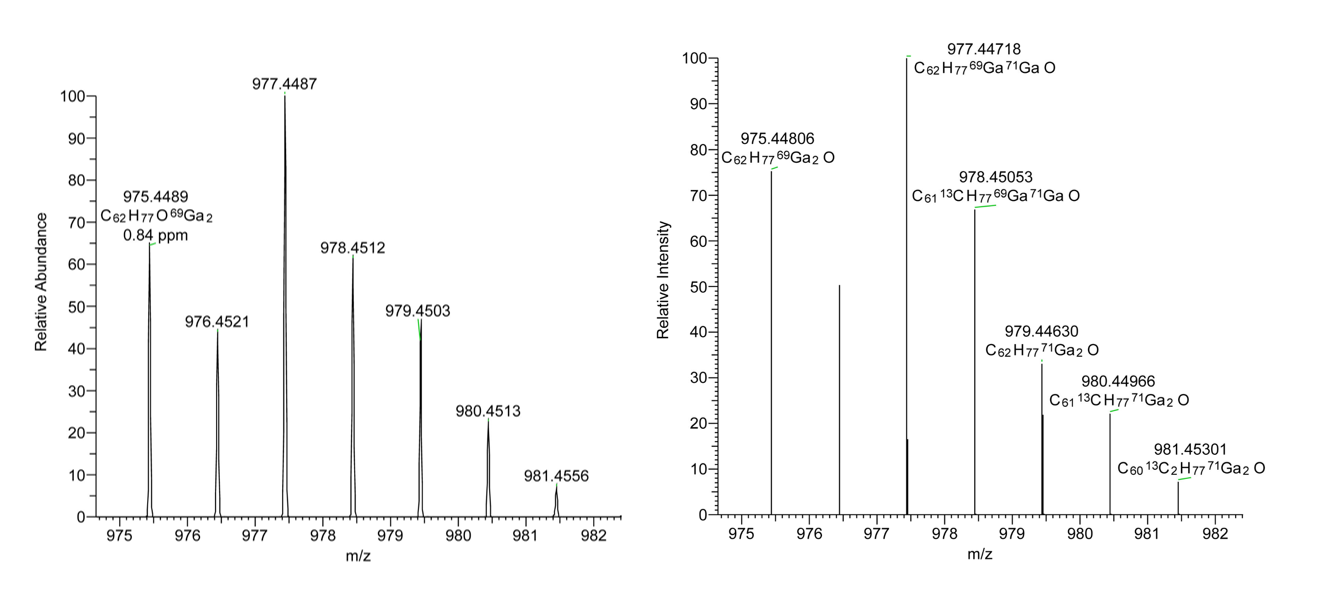


**Figure S64.** HR-MS (APCI- Orbitrap) (positive mode) of (GaTer)_2_(C_2_H_2_) (**4**) showing the [M+O+H]^+^ peak at m/z 975.4489 (calcd. 975.4480; 0.84 ppm error) (left) and simulation (right).


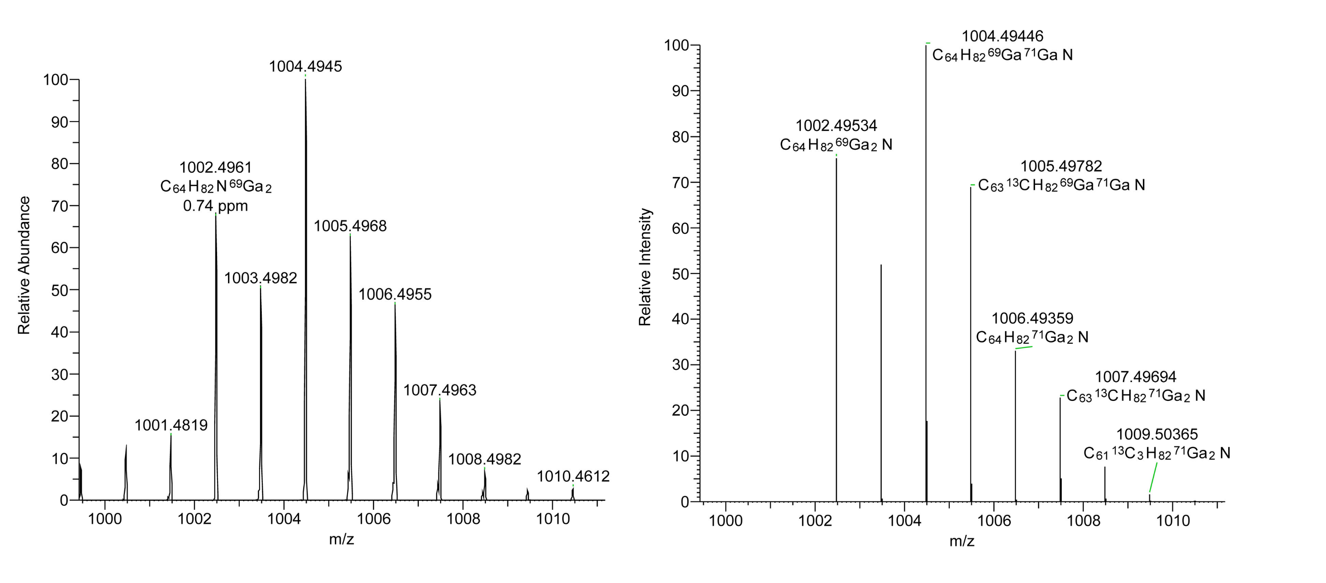


**Figure S65.** HR-MS (APCI-Orbitrap) (positive mode) of (GaTer)_2_(C_2_H_2_)_2_(NH_3_)_2_ (**6**) showing the [M-NH_3_+H]^+^ peak at m/z 1002.4961 (calcd. 1002.4953; 0.74 ppm error) (left) and simulation (right).


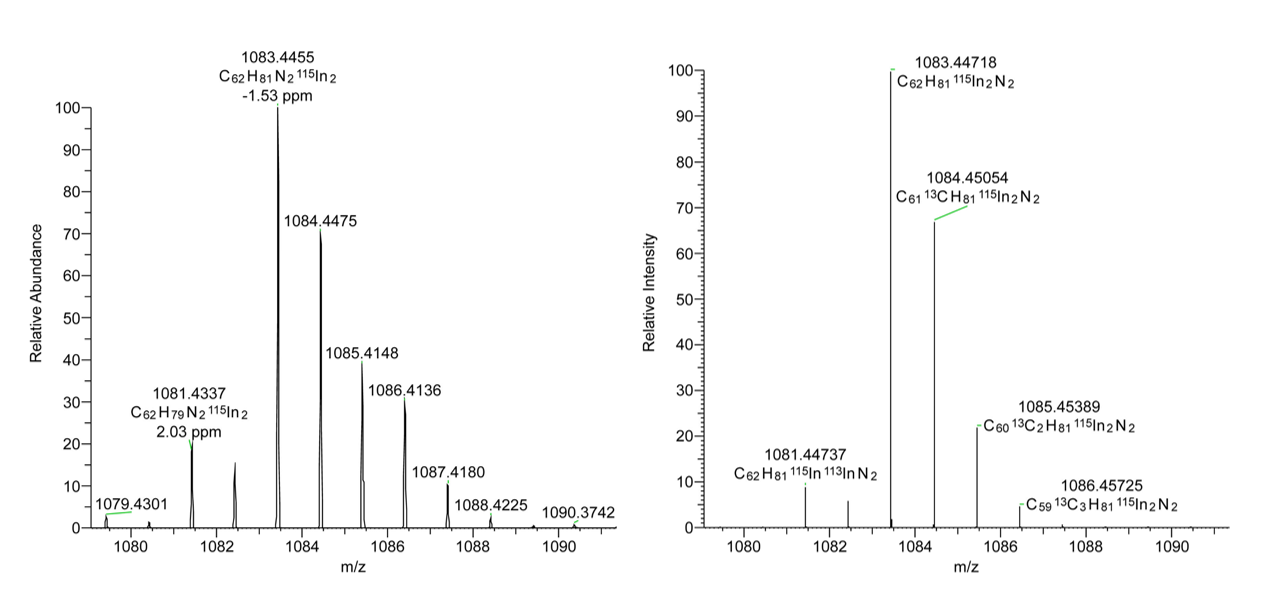


**Figure S66.** HR-MS (APCI- Orbitrap) (positive mode) of (InTer)_2_(C_2_H_2_)(µ-NH_2_)_2_ (**7**) showing the [M+H]^+^ peak at m/z 1083.4455 (calcd. 1083.4471; -1.53 ppm error) (left) and simulation (right).


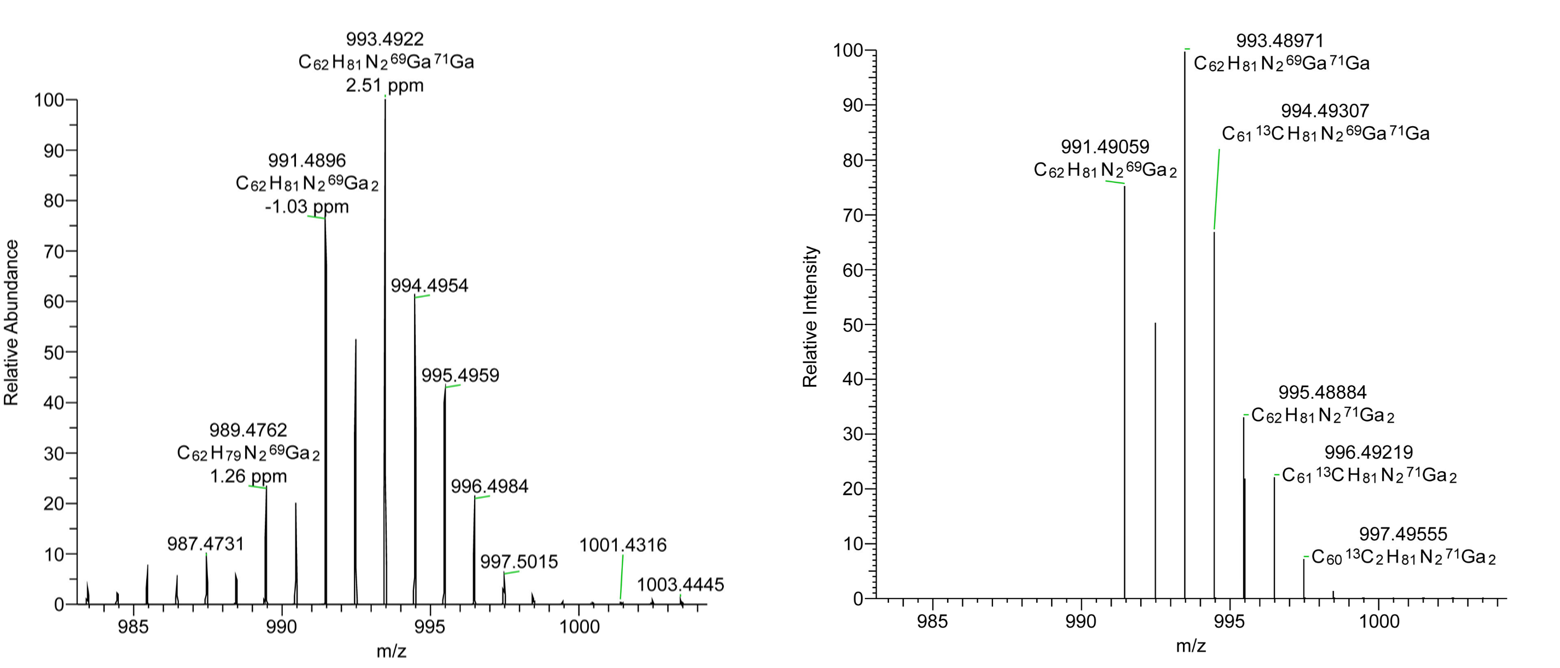


**Figure S67.** HR-MS (APCI- Orbitrap) (positive mode) of (GaTer)_2_(C_2_H_2_)(µ-NH_2_)_2_ (**8**) showing the [M+H]^+^ peak at m/z 993.4922 (calcd. 993.4897; 2.51 ppm error) (left) and simulation (right).

# X-ray crystallographic studies

**Table S1.** Crystal data and structure refinement for **1** (CCDC 2432895).

Empirical formula C_62_H_76_In_2_

Formula weight 1050.86

Crystal color, shape, size yellow block, 0.119 × 0.136 × 0.362 mm3

Temperature 173(2) K

Wavelength 0.71073 Å

Crystal system, space group Monoclinic, *P*2_1_*/n*

Unit cell dimensions a = 12.8199(7) Å α = 90°

b = 25.8688(12) Å β = 101.477(2)°

c = 17.0976(10) Å γ = 90°

Volume 5556.8(5) Å3

*Z* 4

Density (calculated) 1.256 mg/m3

Absorption coefficient 0.865 mm–1

F(000) 2184

***Data collection***

Diffractometer Venture D8, Bruker

Source Iμ3.0, Incoatec

Detector Photon III

Theta range for data collection 1.985 to 28.304°

Index ranges –17≤h≤17, –34≤k≤34, –22≤l≤22

Reflections collected 180821

Independent reflections 13807 [Rint = 0.0642]

Observed Reflections 11160

Completeness to theta = 25.242° 100%

***Solution and Refinement***

Absorption correction Multi-scan

Max. and min. transmission 0.746 and 0.693

Solution Intrinsic methods

Refinement method Full-matrix least-squares on F2

Weighting scheme w = [σ2Fo2 + AP2 + BP]–1, with

P = (Fo2 + 2 Fc2)/3, A = 0.0355, B = 3.8504

Data / restraints / parameters 5595 / 8 / 345

Goodness-of-fit on F2 1.049

Final R indices [I>2σ(I)] R1 = 0.0300, wR2 = 0.0748

R indices (all data) R1 = 0.0414, wR2 = 0.0837

Largest diff. peak and hole 1.542 and –0.452 e·Å–3

Goodness-of-fit = [Σ[*w*(F_o_^2^ − F_c_^2^)^2^]/N_observns_ − N_params_)]^1/2^, all data. R1 = Σ(|F_o_| − |F_c_|) / Σ |F_o_|. *w*R2 = [Σ[*w*(F_o_^2^ − F_c_^2^)^2^] / Σ [*w*(F_o_^2^)^2^]]^1/2^.

**Table S2.** Crystal data and structure refinement for **2** (CCDC 2432896).

Empirical formula C_64_H_78_In_2_

Formula weight 1076.90

Crystal color, shape, size yellow block, 0.102 × 0.202 × 0.226 mm3

Temperature 173(2) K

Wavelength 0.71073 Å

Crystal system, space group Orthorhombic, *P*2_1_2_1_2_1_

Unit cell dimensions a = 11.0758(8) Å α = 90°

b = 18.7045(15) Å β = 90°

c = 27.215(2) Å γ = 90°

Volume 5638.0(8) Å3

*Z* 4

Density (calculated) 1.269 mg/m3

Absorption coefficient 0.855 mm–1

F(000) 2240

***Data collection***

Diffractometer Venture D8, Bruker

Source Iμ3.0, Incoatec

Detector Photon III

Theta range for data collection 1.851 to 33.181°

Index ranges –17≤h≤17, –27≤k≤28, –41≤l≤41

Reflections collected 206273

Independent reflections 21513 [Rint = 0.0696]

Observed Reflections 18836

Completeness to theta = 25.242° 100%

***Solution and Refinement***

Absorption correction Multi-scan

Max. and min. transmission 0.747 and 0.684

Solution Intrinsic methods

Refinement method Full-matrix least-squares on F2

Weighting scheme w = [σ2Fo2 + AP2 + BP]–1, with

P = (Fo2 + 2 Fc2)/3, A = 0.0150, B = 2.2288

Data / restraints / parameters 21513 / 0 / 595

Goodness-of-fit on F2 1.068

Final R indices [I>2σ(I)] R1 = 0.0310, wR2 = 0.0582

R indices (all data) R1 = 0.0418, wR2 = 0.0631

Largest diff. peak and hole 0.537 and –0.539 e·Å-3

Goodness-of-fit = [Σ[*w*(F_o_^2^ − F_c_^2^)^2^]/N_observns_ − N_params_)]^1/2^, all data. R1 = Σ(|F_o_| − |F_c_|) / Σ |F_o_|. *w*R2 = [Σ[*w*(F_o_^2^ − F_c_^2^)^2^] / Σ [*w*(F_o_^2^)^2^]]^1/2^.

**Table S3.** Crystal data and structure refinement for **3** (CCDC 2432897).

Empirical formula C_64_H_78_Ga_2_

Formula weight 986.70

Crystal color, shape, size colorless block, 0.202 × 0.281 × 0.309 mm3

Temperature 153(2) K

Wavelength 0.71073 Å

Crystal system, space group Triclinic, *P*–1

Unit cell dimensions a = 11.2830(4) Å α = 76.4260(10)°

b = 14.1110(5) Å β = 85.565(2)°

c = 18.5671(7) Å γ = 79.4520(10)°

Volume 2823.19(18) Å3

*Z* 2

Density (calculated) 1.161 mg/m3

Absorption coefficient 0.991 mm–1

F(000) 1048

***Data collection***

Diffractometer Venture D8, Bruker

Source Iμ3.0, Incoatec

Detector Photon III

Theta range for data collection 1.837 to 27.493°

Index ranges –14≤h≤14, –18≤k≤18, –24≤l≤24

Reflections collected 124916

Independent reflections 12929 [Rint = 0.0423]

Observed Reflections 11530

Completeness to theta = 25.242° 99.7%

***Solution and Refinement***

Absorption correction Multi-scan

Max. and min. transmission 0.746 and 0.700

Solution Intrinsic methods

Refinement method Full-matrix least-squares on F2

Weighting scheme w = [σ2Fo2 + AP2 + BP]–1, with

P = (Fo2 + 2 Fc2)/3, A = 0.0739, B = 1.5443

Data / restraints / parameters 12929 / 0 / 595

Goodness-of-fit on F2 1.113

Final R indices [I>2σ(I)] R1 = 0.0390, wR2 = 0.1191

R indices (all data) R1 = 0.0444, wR2 = 0.1273

Largest diff. peak and hole 1.643 and –0.988 e·Å–3

Goodness-of-fit = [Σ[*w*(F_o_^2^ − F_c_^2^)^2^]/N_observns_ − N_params_)]^1/2^, all data. R1 = Σ(|F_o_| − |F_c_|) / Σ |F_o_|. *w*R2 = [Σ[*w*(F_o_^2^ − F_c_^2^)^2^] / Σ [*w*(F_o_^2^)^2^]]^1/2^.

**Table S4.** Crystal data and structure refinement for **4** (CCDC 2432898).

Empirical formula C_62_H_76_Ga_2_

Formula weight 960.66

Crystal color, shape, size colorless block, 0.174 × 0.225 × 0.233 mm3

Temperature 173(2) K

Wavelength 0.71073 Å

Crystal system, space group Orthorhombic, *Pna*2_1_

Unit cell dimensions a = 16.5084(4) Å α = 90°

b = 16.0641(5) Å β = 90°

c = 20.2383(6) Å γ = 90°

Volume 5367.0(3) Å3

*Z* 4

Density (calculated) 1.189 mg/m3

Absorption coefficient 1.040 mm–1

F(000) 2040

***Data collection***

Diffractometer Venture D8, Bruker

Source Iμ3.0, Incoatec

Detector Photon III

Theta range for data collection 2.013 to 30.054°

Index ranges –22≤h≤23, –22≤k≤22, –29≤l≤29

Reflections collected 170869

Independent reflections 15738 [Rint = 0.0580]

Observed Reflections 14246

Completeness to theta = 25.242° 100%

***Solution and Refinement***

Absorption correction Multi-scan

Max. and min. transmission 0.746 and 0.665

Solution Intrinsic methods

Refinement method Full-matrix least-squares on F2

Weighting scheme w = [σ2Fo2 + AP2 + BP]–1, with

P = (Fo2 + 2 Fc2)/3, A = 0.0574, B = 1.2690

Data / restraints / parameters 15738 / 1 / 578

Goodness-of-fit on F2 1.085

Final R indices [I>2σ(I)] R1 = 0.0372, wR2 = 0.0926

R indices (all data) R1 = 0.0441, wR2 = 0.0983

Largest diff. peak and hole 1.751 and –0.401 e·Å–3

Type, twin law, domain ratio Inversion, –1 0 0 0 –1 0 0 0 –1. 92:8

Goodness-of-fit = [Σ[*w*(F_o_^2^ − F_c_^2^)^2^]/N_observns_ − N_params_)]^1/2^, all data. R1 = Σ(|F_o_| − |F_c_|) / Σ |F_o_|. *w*R2 = [Σ[*w*(F_o_^2^ − F_c_^2^)^2^] / Σ [*w*(F_o_^2^)^2^]]^1/2^.

**Table S5.** Crystal data and structure refinement for **5**·2C_6_H_6_ (CCDC 2432899).

Empirical formula C_76_H_96_In_2_N_2_

Formula weight 1267.18

Crystal color, shape, size colorless block, 0.166 × 0.222 × 0.430 mm3

Temperature 173(2) K

Wavelength 0.71073 Å

Crystal system, space group Triclinic, *P*–1

Unit cell dimensions a = 10.5264(12) Å α = 66.387(4)°

b = 13.3823(15) Å β = 75.672(4)°

c = 13.7883(13) Å γ = 81.862(4)°

Volume 1722.4(3) Å3

*Z* 1

Density (calculated) 1.222 mg/m3

Absorption coefficient 0.710 mm–1

F(000) 664

***Data collection***

Diffractometer Venture D8, Bruker

Source Iμ3.0, Incoatec

Detector Photon III

Theta range for data collection 1.999 to 30.579°

Index ranges –15≤h≤15, –19≤k≤19, –19≤l≤19

Reflections collected 81010

Independent reflections 10576 [Rint = 0.0651]

Observed Reflections 9405

Completeness to theta = 25.242° 100%

***Solution and Refinement***

Absorption correction Multi-scan

Max. and min. transmission 0.746 and 0.640

Solution Intrinsic methods

Refinement method Full-matrix least-squares on F2

Weighting scheme w = [σ2Fo2 + AP2 + BP]–1, with

P = (Fo2 + 2 Fc2)/3, A = 0.0413, B = 1.0288

Data / restraints / parameters 10576 / 0 / 370

Goodness-of-fit on F2 1.064

Final R indices [I>2σ(I)] R1 = 0.0335, wR2 = 0.0858

R indices (all data) R1 = 0.0400, wR2 = 0.0920

Largest diff. peak and hole 1.835 and –0.725 e·Å–3

Goodness-of-fit = [Σ[*w*(F_o_^2^ − F_c_^2^)^2^]/N_observns_ − N_params_)]^1/2^, all data. R1 = Σ(|F_o_| − |F_c_|) / Σ |F_o_|. *w*R2 = [Σ[*w*(F_o_^2^ − F_c_^2^)^2^] / Σ [*w*(F_o_^2^)^2^]]^1/2^.

**Table S6.** Crystal data and structure refinement for **6**·2C_6_H_6_ (CCDC 2432900).

Empirical formula C_76_H_96_Ga_2_N_2_

Formula weight 1176.98

Crystal color, shape, size colorless block, 0.100 × 0.117 × 0.217 mm3

Temperature 173(2) K

Wavelength 0.71073 Å

Crystal system, space group Triclinic, *P*–1

Unit cell dimensions a = 10.5154(5) Å α = 66.7530(10)°

b = 13.4641(6) Å β = 73.298(2)°

c = 13.5633(6) Å γ = 81.028(2)°

Volume 1687.98(13) Å3

*Z* 1

Density (calculated) 1.158 mg/m3

Absorption coefficient 0.840 mm–1

F(000) 628

***Data collection***

Diffractometer Venture D8, Bruker

Source Iμ3.0, Incoatec

Detector Photon III

Theta range for data collection 2.024 to 28.328°

Index ranges –14≤h≤14, –17≤k≤17, –18≤l≤18

Reflections collected 65316

Independent reflections 8391 [Rint = 0.0493]

Observed Reflections 7676

Completeness to theta = 25.242° 99.9%

***Solution and Refinement***

Absorption correction Multi-scan

Max. and min. transmission 0.746 and 0.710

Solution Intrinsic methods

Refinement method Full-matrix least-squares on F2

Weighting scheme w = [σ2Fo 2+ AP2 + BP]–1, with

P = (Fo 2+ 2 Fc2)/3, A = 0.0320, B = 0.8957

Data / restraints / parameters 8391 / 0 / 370

Goodness-of-fit on F2 1.045

Final R indices [I>2σ(I)] R1 = 0.0302, wR2 = 0.0767

R indices (all data) R1 = 0.0347, wR2 = 0.0802

Largest diff. peak and hole 0.579 and –0.341 e·Å–3

Goodness-of-fit = [Σ[*w*(F_o_^2^ − F_c_^2^)^2^]/N_observns_ − N_params_)]^1/2^, all data. R1 = Σ(|F_o_| − |F_c_|) / Σ |F_o_|. *w*R2 = [Σ[*w*(F_o_^2^ − F_c_^2^)^2^] / Σ [*w*(F_o_^2^)^2^]]^1/2^.

**Table S7.** Crystal data and structure refinement for **7** (CCDC 2432901).

Empirical formula C_62_H_80_In_2_N_2_

Formula weight 1082.92

Crystal color, shape, size colorless block, 0.120 × 0.200 × 0.312 mm3

Temperature 173(2) K

Wavelength 0.71073 Å

Crystal system, space group Monoclinic, *Pn*

Unit cell dimensions a = 13.9110(6) Å α = 90°

b = 10.6256(6) Å β = 99.620(2)°

c = 19.5781(10) Å γ = 90°

Volume 2853.2(3) Å3

*Z* 2

Density (calculated) 1.260 mg/m3

Absorption coefficient 0.846 mm–1

F(000) 1128

***Data collection***

Diffractometer Venture D8, Bruker

Source Iμ3.0, Incoatec

Detector Photon III

Theta range for data collection 1.917 to 28.321°

Index ranges –18≤h≤18, –14≤k≤14, –26≤l≤26

Reflections collected 82716

Independent reflections 14145 [Rint = 0.0846]

Observed Reflections 13034

Completeness to theta = 25.242° 100%

***Solution and Refinement***

Absorption correction Multi-scan

Max. and min. transmission 0.735 and 0.692

Solution Intrinsic methods

Refinement method Full-matrix least-squares on F2

Weighting scheme w = [σ2Fo2 + AP2 + BP]–1, with

P = (Fo2 + 2 Fc2)/3, A = 0.0264, B = 0.2022

Data / restraints / parameters 14145 / 2 / 596

Goodness-of-fit on F2 1.046

Final R indices [I>2σ(I)] R1 = 0.0353, wR2 = 0.0718

R indices (all data) R1 = 0.0406, wR2 = 0.0757

Largest diff. peak and hole 0.535 and –0.315 e·Å–3

Type, twin law, domain ratio Inversion, –1 0 0 0 –1 0 0 0 –1. 89:11

Goodness-of-fit = [Σ[*w*(F_o_^2^ − F_c_^2^)^2^]/N_observns_ − N_params_)]^1/2^, all data. R1 = Σ(|F_o_| − |F_c_|) / Σ |F_o_|. *w*R2 = [Σ[*w*(F_o_^2^ − F_c_^2^)^2^] / Σ [*w*(F_o_^2^)^2^]]^1/2^.

**Table S8.** Crystal data and structure refinement for **8** (CCDC 2432902).

Empirical formula C_62_H_80_Ga_2_N_2_

Formula weight 992.72

Crystal color, shape, size colorless plate, 0.064 × 0.252 × 0.255 mm3

Temperature 173(2) K

Wavelength 0.71073 Å

Crystal system, space group Monoclinic, *P*2_1_*/n*

Unit cell dimensions a = 11.1044(5) Å α = 90°

b = 18.9178(9) Å β = 96.529(2)°

c = 13.6125(7) Å γ = 90°

Volume 2841.0(2)Å3

*Z* 2

Density (calculated) 1.160 mg/m3

Absorption coefficient 0.986 mm–1

F(000) 1056

***Data collection***

Diffractometer Venture D8, Bruker

Source Iμ3.0, Incoatec

Detector Photon III

Theta range for data collection 2.137 to 30.570°

Index ranges –15≤h≤14, –27≤k≤27, –19≤l≤19

Reflections collected 96217

Independent reflections 8712 [Rint = 0.0563]

Observed Reflections 7059

Completeness to theta = 25.242° 100%

***Solution and Refinement***

Absorption correction Multi-scan

Max. and min. transmission 0.746 and 0.676

Solution Intrinsic methods

Refinement method Full-matrix least-squares on F2

Weighting scheme w = [σ2Fo2 + AP2 + BP]–1, with

P = (Fo2 + 2 Fc2)/3, A = 0.0976, B = 1.5549

Data / restraints / parameters 8712 / 174 / 445

Goodness-of-fit on F2 1.047

Final R indices [I>2σ(I)] R1 = 0.0595, wR2 = 0.1684

R indices (all data) R1 = 0.0729, wR2 = 0.1827

Largest diff. peak and hole 1.271 and –0.774 e·Å–3

Goodness-of-fit = [Σ[*w*(F_o_^2^ − F_c_^2^)^2^]/N_observns_ − N_params_)]^1/2^, all data. R1 = Σ(|F_o_| − |F_c_|) / Σ |F_o_|. *w*R2 = [Σ[*w*(F_o_^2^ − F_c_^2^)^2^] / Σ [*w*(F_o_^2^)^2^]]^1/2^.

**Crystal structure data for 1.**

**Data collection**

A yellow, block-shaped specimen of C_62_H_76_In_2_, approximate dimensions 0.119 × 0.136 × 0.263 mm^3^, was used for the X-ray crystallographic analysis. The X-ray intensity data of 25089 were measured on a Bruker D8 Venture KAPPA diffractometer equipped with a microfocus sealed tube (λ = 0.71073 Å) and a multilayer mirror monochromator at 173(2) K. The data collection was carried out using Mo Kα radiation (graphite monochromator) with a frame time of 1 and 5 seconds and a detector distance of 4.00 cm. Complete and redundant data were collected to a resolution of a minimum of 0.75 Å, and ten sets of frames were collected with 0.5º ω and φ scans (3128 frames). The total exposure time was 2.61 hours. The frames were integrated with the SAINT V8.41 package using a narrow-frame algorithm.^[57]^ The integration of the data using a monoclinic unit cell yielded a total of 180821 reflections to a maximum θ angle of 28.34° (0.75 Å resolution), of which 13807 were independent (average redundancy 13.10, completeness = 100.0%, R_int_ = 6.42%, R_sig_ = 2.54%) and 11160 (80.8%) were greater than 2σ(*F*^2^). The final cell constants of a = 12.8199(7) Å, b = 25.8688(12) Å, c = 17.0976(10) Å, α = 90°, β = 101.477(2)°, γ = 90°, volume = 5556.8(5) Å^3^, are based upon the refinement of the XYZ-centroids of 9811 reflections above 20 σ(*I*) with 2.21° < 2θ < 28.16°. Data were corrected for absorption effects using the Multi-Scan method in SADABS 2016/2.^[58]^ The calculated minimum and maximum transmission coefficients (based on crystal size) are 0.693 and 0.746.⁠

**Structure solution and refinement**

The space group *P*2_1_/*n* (14) was determined based on intensity statistics and systematic absences. The structure was solved by XT, VERSION 2018/2 and refined with full-matrix least squares / difference Fourier cycles using XL; *Z* = 2 for the formula unit C_62_H_76_In_2_.^[59,60]^ Non-hydrogen atoms were refined with anisotropic displacement parameters. The hydrogen atoms were placed in ideal positions and refined as riding atoms with relative isotropic displacement parameters. The final anisotropic full-matrix least-squares refinement on *F*^2^ with 593 variables against 13807 data points and converged at *R*_1_ = 2.98%, for the observed data and w*R*_2_ = 8.28% for all data. The goodness-of-fit on *F*^2^ was 1.05. The largest peak in the final difference electron density synthesis was 1.54 e^−^/Å^3^ and the deepest hole was −0.45 e^−^/Å^3^ with an RMS deviation of 0.062 e^−^/Å^3^. On the basis of the final model, the calculated density was 1.26 g/cm^3^ and *F*(000), 2184 e^−^.

**Crystal structure data for 2.**

**Data collection**

A yellow, block shaped specimen of C_64_H_78_In_2_, approximate dimensions 0.102 × 0.202 × 0.226 mm^3^, was used for the X-ray crystallographic analysis. The X-ray intensity data of 25034 were measured on a Bruker D8 Venture KAPPA diffractometer equipped with a microfocus sealed tube (λ = 0.71073 Å) and a multilayer mirror monochromator at 173(2) K. The data collection was carried out using Mo Kα radiation (graphite monochromator) with a frame time of 0.75 and 5 seconds and a detector distance of 4.00 cm. Complete and redundant data were collected to a resolution of a minimum of 0.65 Å, and eight sets of frames were collected with 1º ω and φ scans (1296 frames). The total exposure time was 1.25 hours. The frames were integrated with the SAINT V8.41 package using a narrow-frame algorithm.^[57]^ The integration of the data using an orthorhombic unit cell yielded a total of 206273 reflections to a maximum θ angle of 33.17° (0.65 Å resolution), of which 21513 were independent (average redundancy 9.59, completeness = 100.0%, R_int_ = 6.96%, R_sig_ = 3.69%) and 18836 (87.6%) were greater than 2σ(*F*^2^). The final cell constants of a = 11.0758(8) Å, b = 18.7045(15) Å, c = 27.215(2) Å, α = 90°, β = 90°, γ = 90°, volume = 5638.0(8) Å^3^, are based upon the refinement of the XYZ-centroids of 9898 reflections above 20 σ(*I*) with 2.26° < 2θ < 28.56°. Data were corrected for absorption effects using the Multi-Scan method in SADABS 2016/2.^[58]^ The calculated minimum and maximum transmission coefficients (based on crystal size) are 0.684 and 0.747.⁠

**Structure solution and refinement**

The space group *P*2_1_2_1_2_1_ (19) was determined based on intensity statistics and systematic absences. The structure was solved by SHELXT 2018/2 and refined with full-matrix least squares / difference Fourier cycles using SHELXL-2019/2; *Z* = 4 for the formula unit C_64_H_78_In_2_.⁠^[59,60]^ Non-hydrogen atoms were refined with anisotropic displacement parameters. The hydrogen atoms were placed in ideal positions and refined as riding atoms with relative isotropic displacement parameters. The final anisotropic full-matrix least-squares refinement on *F*^2^ with 595 variables against 21513 data points and converged at *R*_1_ = 3.10%, for the observed data and w*R*_2_ = 6.31% for all data. The goodness-of-fit on *F*^2^ was 1.07. The largest peak in the final difference electron density synthesis was 0.54 e^−^/Å^3^ and the deepest hole was −0.54 e^−^/Å^3^ with an RMS deviation of 0.066 e^−^/Å^3^. On the basis of the final model, the calculated density was 1.27 g/cm^3^ and *F*(000), 2240 e^−^.

**Crystal structure data for 3.**

**Data collection**

A colourless, block shaped specimen of C_64_H_78_Ga_2_, approximate dimensions 0.202 × 0.281 × 0.31 mm^3^, was used for the X-ray crystallographic analysis. The X-ray intensity data of 25067 were measured on a Bruker D8 Venture KAPPA diffractometer equipped with a microfocus sealed tube (λ = 0.71073 Å) at 153(2) K. The data collection was carried out using Mo Kα radiation (graphite monochromator) with a frame time of 1 and 3 seconds and a detector distance of 4.00 cm. Complete and redundant data were collected to a resolution of a minimum of 0.77 Å, and ten sets of frames were collected with 0.5º ω and φ scans (3544 frames). The total exposure time was 2.44 hours. The frames were integrated with the SAINT V8.40B package using a narrow-frame algorithm.⁠^[57]^ The integration of the data using a triclinic unit cell yielded a total of 124916 reflections to a maximum θ angle of 27.52° (0.77 Å resolution), of which 12918 were independent (average redundancy 9.67, completeness = 99.7%, R_int_ = 4.19%, R_sig_ = 2.01%) and 11530 (89.3%) were greater than 2σ(*F*^2^). The final cell constants of a = 11.2830(4) Å, b = 14.1110(5) Å, c = 18.5671(7) Å, α = 76.4260(10)°, β = 85.565(2)°, γ = 79.4520(10)°, volume = 2823.19(18) Å^3^, are based upon the refinement of the XYZ-centroids of 9716 reflections above 20 σ(*I*) with 2.26° < 2θ < 27.45°. Data were corrected for absorption effects using the Multi-Scan method in SADABS 2016/2.^[58]^ The calculated minimum and maximum transmission coefficients (based on crystal size) are 0.700 and 0.746.⁠

**Structure solution and refinement**

The space group *P*–1 (2) was determined based on intensity statistics and systematic absences. The structure was solved by SHELXT 2018/2 and refined with full-matrix least squares / difference Fourier cycles using SHELXL-2019/1; *Z* = 2 for the formula unit C_64_H_78_Ga_2_.⁠^[59,60]^ Non-hydrogen atoms were refined with anisotropic displacement parameters. The hydrogen atoms were placed in ideal positions and refined as riding atoms with relative isotropic displacement parameters. The final anisotropic full-matrix least-squares refinement on *F*^2^ with 595 variables against 12929 data points and converged at *R*_1_ = 3.90%, for the observed data and w*R*_2_ = 12.73% for all data. The goodness-of-fit on *F*^2^ was 1.11. The largest peak in the final difference electron density synthesis was 1.64 e^−^/Å^3^ and the deepest hole was −0.99 e^−^/Å^3^ with an RMS deviation of 0.127 e^−^/Å^3^. On the basis of the final model, the calculated density was 1.16 g/cm^3^ and *F*(000), 1048 e^−^.

**Crystal structure data for 4.**

**Data collection**

A colourless, block shaped specimen of C_62_H_76_Ga_2_, approximate dimensions 0.174 × 0.225 × 0.233 mm^3^, was used for the X-ray crystallographic analysis. The X-ray intensity data of 25050 were measured on a Bruker D8 Venture KAPPA diffractometer equipped with a microfocus sealed tube (λ = 0.71073 Å) and a multilayer mirror monochromator at 173(2) K. The data collection was carried out using Mo Kα radiation (graphite monochromator) with a frame time of 1 and 23 seconds and a detector distance of 4.00 cm. Complete and redundant data were collected to a resolution of a minimum of 0.71 Å, and eight sets of frames were collected with 0.5º ω and φ scans (2706 frames). The total exposure time was 5.53 hours. The frames were integrated with the SAINT V8.41 package using a narrow-frame algorithm.⁠^[57]^ The integration of the data using an orthorhombic unit cell yielded a total of 170869 reflections to a maximum θ angle of 30.07° (0.71 Å resolution), of which 15738 were independent (average redundancy 10.86, completeness = 100.0%, R_int_ = 5.80%, R_sig_ = 3.00%) and 14246 (90.5%) were greater than 2σ(*F*^2^). The final cell constants of a = 16.5084(4) Å, b = 16.0641(5) Å, c = 20.2383(6) Å, α = 90°, β = 90°, γ = 90°, volume = 5367.0(3) Å^3^, are based upon the refinement of the XYZ-centroids of 9864 reflections above 20 σ(*I*) with 2.54° < 2θ < 29.57°. Data were corrected for absorption effects using the Multi-Scan method in SADABS 2016/2.^[58]^ The calculated minimum and maximum transmission coefficients (based on crystal size) are 0.665 and 0.746.⁠

**Structure solution and refinement**

The space group *Pna*2_1_ (33) was determined based on intensity statistics and systematic absences. The structure was solved by SHELXT 2018/2 and refined with full-matrix least squares / difference Fourier cycles using SHELXL-2019/2; *Z* = 4 for the formula unit C_62_H_76_Ga_2_.⁠^[59,60]^ Non-hydrogen atoms were refined with anisotropic displacement parameters. The hydrogen atoms were placed in ideal positions and refined as riding atoms with relative isotropic displacement parameters. The final anisotropic full-matrix least-squares refinement on *F*^2^ with 578 variables against 15738 data points and 1 restraints converged at *R*_1_ = 3.72%, for the observed data and w*R*_2_ = 9.83% for all data. The goodness-of-fit on *F*^2^ was 1.08. The largest peak in the final difference electron density synthesis was 1.75 e^−^/Å^3^ and the deepest hole was −0.40 e^−^/Å^3^ with an RMS deviation of 0.076 e^−^/Å^3^. On the basis of the final model, the calculated density was 1.19 g/cm^3^ and *F*(000), 2040 e^−^.

**Crystal structure data for 5.**

**Data collection**

A colourless, block shaped specimen of C_76_H_96_In_2_N_2_, approximate dimensions 0.166 × 0.22 × 0.43 mm^3^, was used for the X-ray crystallographic analysis. The X-ray intensity data of 25043 were measured on a Bruker D8 Venture KAPPA diffractometer equipped with a microfocus sealed tube (λ = 0.71073 Å) and a multilayer mirror monochromator at at 173(2) K. The data collection was carried out using Mo Kα radiation (graphite monochromator) with a frame time of 0.75 and 5 seconds and a detector distance of 4.00 cm. Complete and redundant data were collected to a resolution of a minimum of 0.70 Å, and ten sets of frames were collected with 1º ω and φ scans (1667 frames). The total exposure time was 1.77 hours. The frames were integrated with the SAINT V8.41 package using a narrow-frame algorithm.⁠^[57]^ The integration of the data using a triclinic unit cell yielded a total of 81010 reflections to a maximum θ angle of 30.56° (0.70 Å resolution), of which 10576 were independent (average redundancy 7.66, completeness = 100.0%, R_int_ = 6.51%, R_sig_ = 3.63%) and 9405 (88.9%) were greater than 2σ(*F*^2^). The final cell constants of a = 10.5264(12) Å, b = 13.3823(15) Å, c = 13.7883(13) Å, α = 66.387(4)°, β = 75.672(4)°, γ = 81.862(4)°, volume = 1722.4(3) Å^3^, are based upon the refinement of the XYZ-centroids of 9615 reflections above 20 σ(*I*) with 2.31° < 2θ < 30.49°. Data were corrected for absorption effects using the Multi-Scan method in SADABS 2016/2.^[58]^ The calculated minimum and maximum transmission coefficients (based on crystal size) are 0.640 and 0.746.

**Structure solution and refinement**

The space group *P–*1 (2) was determined based on intensity statistics and systematic absences. The structure was solved by XT, VERSION 2018/2 and refined with full-matrix least squares / difference Fourier cycles using SHELXL-2019/2; Z = 2 for the formula unit C_76_H_96_In_2_N_2_.⁠^[59,60]^ Non-hydrogen atoms were refined with anisotropic displacement parameters. The hydrogen atoms were placed in ideal positions and refined as riding atoms with relative isotropic displacement parameters. The final anisotropic full-matrix least-squares refinement on *F*^2^ with 370 variables against 10576 data points and converged at *R*_1_ = 3.35%, for the observed data and w*R*_2_ = 9.20% for all data. The goodness-of-fit on *F*^2^ was 1.06. The largest peak in the final difference electron density synthesis was 1.83 e^−^/Å^3^ and the deepest hole was −0.72 e^−^/Å^3^ with an RMS deviation of 0.080 e^−^/Å^3^. On the basis of the final model, the calculated density was 1.22 g/cm^3^ and *F*(000), 664 e^−^.

**Crystal structure data for 6.**

**Data collection**

A colourless, block shaped specimen of C_76_H_96_Ga_2_N_2_, approximate dimensions 0.1 × 0.117 × 0.217 mm^3^, was used for the X-ray crystallographic analysis. The X-ray intensity data of 25007 were measured on a Bruker D8 Venture KAPPA diffractometer equipped with a microfocus sealed tube (λ = 0.71073 Å) and a multilayer mirror monochromator at 173(2) K. The data collection was carried out using Mo Kα radiation (graphite monochromator) with a frame time of 0.75 and 10 seconds and a detector distance of 4.00 cm. Complete and redundant data were collected to a resolution of a minimum of 0.75 Å, and eight sets of frames were collected with 1º ω and φ scans (1605 frames). The total exposure time was 2.99 hours. The frames were integrated with the SAINT V8.41 package using a narrow-frame algorithm.^[57]^ The integration of the data using a triclinic unit cell yielded a total of 65316 reflections to a maximum θ angle of 28.32° (0.75 Å resolution), of which 8391 were independent (average redundancy 7.78, completeness = 99.9%, R_int_ = 4.93%, R_sig_ = 2.69%) and 7676 (91.5%) were greater than 2σ(*F*^2^). The final cell constants of a = 10.5154(5) Å, b = 13.4641(6) Å, c = 13.5633(6) Å, α = 66.7530(10)°, β = 73.298(2)°, γ = 81.028(2)°, volume = 1687.98(13) Å^3^, are based upon the refinement of the XYZ-centroids of 9779 reflections above 20 σ(*I*) with 2.29° < 2θ < 28.24°. Data were corrected for absorption effects using the Multi-Scan method in SADABS 2016/2.⁠^[58]^ The calculated minimum and maximum transmission coefficients (based on crystal size) are 0.710 and 0.746.

**Structure solution and refinement**

The space group *P–*1 (2) was determined based on intensity statistics and systematic absences. The structure was solved by SHELXT 2018/2 and refined with full-matrix least squares / difference Fourier cycles using SHELXL-2019/2; *Z* = 1 for the formula unit C_76_H_96_Ga_2_N_2_.⁠^[59,60]^ Non-hydrogen atoms were refined with anisotropic displacement parameters. The hydrogen atoms were placed in ideal positions and refined as riding atoms with relative isotropic displacement parameters. The final anisotropic full-matrix least-squares refinement on *F*^2^ with 370 variables against 8391 data points and converged at *R*_1_ = 3.02%, for the observed data and w*R*_2_ = 8.02% for all data. The goodness-of-fit on *F*^2^ was 1.04. The largest peak in the final difference electron density synthesis was 0.58 e^−^/Å^3^ and the deepest hole was −0.34 e^−^/Å^3^ with an RMS deviation of 0.048 e^−^/Å^3^. On the basis of the final model, the calculated density was 1.16 g/cm^3^ and *F*(000), 628 e^−^.

**Crystal structure data for 7.**

**Data collection**

A colourless, block shaped specimen of C_62_H_80_In_2_N_2_, approximate dimensions 0.12 × 0.2 × 0.312 mm^3^, was used for the X-ray crystallographic analysis. The X-ray intensity data of 25019 were measured on a Bruker D8 Venture KAPPA diffractometer equipped with a microfocus sealed tube (λ = 0.71073 Å) and a multilayer mirror monochromator at 173(2) K. The data collection was carried out using Mo Kα radiation (graphite monochromator) with a frame time of 0.70 and 1 seconds and a detector distance of 4.00 cm. Complete and redundant data were collected to a resolution of a minimum of 0.75 Å, and eight sets of frames were collected with 1º ω and φ scans (1350 frames). The total exposure time was 0.33 hours. The frames were integrated with the SAINT V8.41 package using a narrow-frame algorithm.⁠^[57]^ The integration of the data using a monoclinic unit cell yielded a total of 82716 reflections to a maximum θ angle of 28.32° (0.75 Å resolution), of which 14145 were independent (average redundancy 5.85, completeness = 100.0%, R_int_ = 8.46%, R_sig_ = 5.67%) and 13034 (92.1%) were greater than 2σ(*F*^2^). The final cell constants of a = 13.9110(6) Å, b = 10.6256(6) Å, c = 19.5781(10) Å, α = 90°, β = 99.620(2)°, γ = 90°, volume = 2853.2(3) Å^3^, are based upon the refinement of the XYZ-centroids of 9882 reflections above 20 σ(*I*) with 2.19° < 2θ < 26.76°. Data were corrected for absorption effects using the Multi-Scan method in SADABS 2016/2.⁠^[58]^ The calculated minimum and maximum transmission coefficients (based on crystal size) are 0.692 and 0.735.

**Structure solution and refinement**

The space group *Pn* (7) was determined based on intensity statistics and systematic absences. The structure was solved by SHELXT 2018/2 and refined with full-matrix least squares / difference Fourier cycles using SHELXL-2019/2; *Z* = 2 for the formula unit C_62_H_80_In_2_N_2_.⁠^[59,60]^ Non-hydrogen atoms were refined with anisotropic displacement parameters. The hydrogen atoms were placed in ideal positions and refined as riding atoms with relative isotropic displacement parameters. The final anisotropic full-matrix least-squares refinement on *F*^2^ with 596 variables against 14145 data points and 2 restraints converged at *R*_1_ = 3.53%, for the observed data and w*R*_2_ = 7.57% for all data. The goodness-of-fit on *F*^2^ was 1.05. The largest peak in the final difference electron density synthesis was 0.54 e^−^/Å^3^ and the deepest hole was −0.32 e^−^/Å^3^ with an RMS deviation of 0.063 e^−^/Å^3^. On the basis of the final model, the calculated density was 1.26 g/cm^3^ and *F*(000), 1128 e^−^.

**Crystal structure data for 8.**

**Data collection**

A colourless, plate shaped specimen of C_62_H_80_Ga_2_N_2_, approximate dimensions 0.064 × 0.252 × 0.255 mm^3^, was used for the X-ray crystallographic analysis. The X-ray intensity data of 25054 were measured on a Bruker D8 Venture KAPPA diffractometer equipped with a microfocus sealed tube (λ = 0.71073 Å) and a multilayer mirror monochromator at 173(2) K. The data collection was carried out using Mo Kα radiation (graphite monochromator) with a frame time of 0.75 and 30 seconds and a detector distance of 4.00 cm. Complete and redundant data were collected to a resolution of a minimum of 0.70 Å, and twelve sets of frames were collected with 0.5º ω and φ scans (3322 frames). The total exposure time was 14.19 hours. The frames were integrated with the SAINT V8.41 package using a narrow-frame algorithm.⁠^[57]^ The integration of the data using a monoclinic unit cell yielded a total of 96217 reflections to a maximum θ angle of 30.55° (0.70 Å resolution), of which 5809 were independent (average redundancy 16.56, completeness = 100.0%, R_int_ = 13.78%, R_sig_ = 2.73%) and 7059 (121.5%) were greater than 2σ(*F*^2^). The final cell constants of a = 11.1044(5) Å, b = 18.9178(9) Å, c = 13.6125(7) Å, α = 90°, β = 96.529(2)°, γ = 90°, volume = 2841.0(2) Å^3^, are based upon the refinement of the XYZ-centroids of 9815 reflections above 20 σ(*I*) with 2.25° < 2θ < 30.05°. Data were corrected for absorption effects using the Multi-Scan method in SADABS 2016/2.⁠^[58]^ The calculated minimum and maximum transmission coefficients (based on crystal size) are 0.676 and 0.746.

**Structure solution and refinement**

The space group *P*2_1_/*n* (14) was determined based on intensity statistics and systematic absences. The structure was solved by XT, VERSION 2018/2 and refined with full-matrix least squares / difference Fourier cycles using SHELXL-2019/2; *Z* = 4 for the formula unit C_26_H_23_Ga_2_N_5_.⁠^[59,60]^ Non-hydrogen atoms were refined with anisotropic displacement parameters. The hydrogen atoms were placed in ideal positions and refined as riding atoms with relative isotropic displacement parameters. The final anisotropic full-matrix least-squares refinement on *F*^2^ with 445 variables against 8712 data points and 174 restraints converged at *R*_1_ = 5.95%, for the observed data and w*R*_2_ = 18.27% for all data. The goodness-of-fit on *F*^2^ was 1.05. The largest peak in the final difference electron density synthesis was 1.27 e^−^/Å^3^ and the deepest hole was −0.77 e^−^/Å^3^ with an RMS deviation of 0.067 e^−^/Å^3^. On the basis of the final model, the calculated density was 1.27 g/cm^3^ and *F*(000), 1056 e^−^.

# Computational details

All the geometry optimizations and frequency calculations reported in this paper were obtained with the ORCA 6.0.1 program.^[61]^ Electron correlation was partially taken into account using the PBE0 functional^[62,63]^ in conjunction with the D3(BJ) dispersion correction suggested by Grimme et al.,^[64,65]^ the resolution-of-identity approach,^[66]^ RI(JCOSX), and the double-ζ quality plus polarization functions def2-SVP^[67]^ basis set for all atoms. Solvent effects (solvent = benzene) were taken into account by using the conductor-like polarizable continuum model (CPCM).^[68]^ All species were characterized by frequency calculations: reactants and adducts exhibited positive definite Hessian matrices, while transition states showed a single negative eigenvalue in their diagonalized force constant matrices. Transition states were located by using the Nudged Elastic Band (NEB)^[69]^ method implemented in ORCA. This level is denoted CPCM-(RI)-PBE0-D3(BJ)/def2-SVP.

Cartesian coordinates (in Å) and free energies (in a.u., at 298 K) of all the stationary points discussed in the text. All calculations have been performed at the CPCM-(RI)-PBE0-D3(BJ)/def2-SVP level.

**1:** G= -2783.18763058

In 5.962270000 18.038924000 4.864598000

In 5.654263000 20.794821000 4.776103000

C 4.753116000 18.574707000 3.091045000

H 4.250296000 17.923597000 2.358078000

C 4.774962000 19.915234000 2.948176000

H 4.336709000 20.411129000 2.067248000

C 6.991059000 16.139752000 5.130991000

C 6.461438000 14.969953000 5.687770000

C 7.229302000 13.796642000 5.679106000

H 6.818688000 12.881385000 6.114820000

C 8.514456000 13.797630000 5.140938000

H 9.106362000 12.879030000 5.145850000

C 9.054132000 14.969034000 4.608544000

H 10.070493000 14.971704000 4.206682000

C 8.292083000 16.139812000 4.598081000

C 5.115173000 14.999874000 6.331753000

C 3.943574000 14.943785000 5.543681000

C 2.701062000 14.982564000 6.184542000

H 1.785742000 14.931516000 5.592154000

C 2.611826000 15.081572000 7.569448000

H 1.631991000 15.108990000 8.053079000

C 3.769252000 15.156118000 8.336208000

H 3.689052000 15.250845000 9.421576000

C 5.032200000 15.116038000 7.736390000

C 4.027853000 14.782084000 4.036416000

H 4.944516000 15.297173000 3.706513000

C 2.856590000 15.412804000 3.290940000

H 3.054510000 15.411822000 2.208175000

H 1.920285000 14.854745000 3.449475000

H 2.690932000 16.454386000 3.604816000

C 4.180542000 13.305414000 3.665216000

H 4.276782000 13.186841000 2.574287000

H 5.071188000 12.862969000 4.134875000

H 3.300671000 12.729687000 3.995111000

C 6.279824000 15.263629000 8.588208000

H 7.131019000 14.910956000 7.987957000

C 6.531869000 16.737609000 8.904956000

H 7.471326000 16.865219000 9.465911000

H 6.602062000 17.338643000 7.985485000

H 5.714157000 17.153162000 9.514910000

C 6.243902000 14.420655000 9.859917000

H 7.209858000 14.484151000 10.384449000

H 5.469620000 14.765962000 10.563152000

H 6.046858000 13.361505000 9.634192000

C 8.786224000 17.456437000 4.086973000

C 8.490226000 17.860771000 2.762609000

C 8.791534000 19.173341000 2.383103000

H 8.546548000 19.515639000 1.376465000

C 9.391264000 20.058189000 3.273719000

H 9.602733000 21.084458000 2.965129000

C 9.710569000 19.642156000 4.561635000

H 10.179509000 20.347932000 5.249448000

C 9.404492000 18.348469000 4.993684000

C 7.903481000 16.874623000 1.767590000

H 7.203749000 16.228944000 2.325839000

C 7.127811000 17.527551000 0.629381000

H 6.614782000 16.755682000 0.035669000

H 6.370976000 18.230652000 1.004371000

H 7.795701000 18.072237000 -0.056922000

C 9.008798000 15.976126000 1.205758000

H 8.586712000 15.228088000 0.516184000

H 9.744818000 16.577272000 0.647939000

H 9.543118000 15.440447000 2.001909000

C 9.743666000 17.902908000 6.403456000

H 9.045328000 17.090569000 6.659382000

C 9.563364000 18.999576000 7.446726000

H 9.665265000 18.579910000 8.459288000

H 10.317007000 19.796103000 7.346492000

H 8.569706000 19.464092000 7.369295000

C 11.159398000 17.325010000 6.450980000

H 11.399575000 16.963354000 7.463416000

H 11.273506000 16.483717000 5.751945000

H 11.901507000 18.092907000 6.178465000

C 5.187961000 22.771026000 5.553067000

C 4.078941000 22.785090000 6.416676000

C 3.600970000 23.997444000 6.920053000

H 2.747437000 24.011053000 7.602708000

C 4.225334000 25.191387000 6.556262000

H 3.850515000 26.141032000 6.946142000

C 5.334287000 25.178920000 5.712270000

H 5.835622000 26.115387000 5.452370000

C 5.829993000 23.966673000 5.212321000

C 3.519719000 21.444243000 6.774876000

C 2.559196000 20.826982000 5.937949000

C 2.216671000 19.493012000 6.184993000

H 1.500528000 18.989096000 5.534086000

C 2.784283000 18.790505000 7.243272000

H 2.520322000 17.743692000 7.409168000

C 3.698883000 19.416285000 8.083103000

H 4.136992000 18.853467000 8.908966000

C 4.091143000 20.739162000 7.859326000

C 1.883952000 21.612712000 4.827980000

H 2.638056000 22.303429000 4.413292000

C 1.365799000 20.749909000 3.682960000

H 2.147003000 20.082003000 3.294246000

H 0.507618000 20.133089000 3.993856000

H 1.019523000 21.391219000 2.858161000

C 0.748525000 22.462591000 5.404902000

H 0.274500000 23.064629000 4.613657000

H -0.024732000 21.816281000 5.850593000

H 1.105964000 23.148194000 6.185465000

C 5.099233000 21.408804000 8.773990000

H 5.579591000 22.211424000 8.192557000

C 6.202980000 20.470413000 9.248225000

H 6.987276000 21.039946000 9.769812000

H 5.828595000 19.710762000 9.952035000

H 6.669016000 19.944344000 8.402345000

C 4.385519000 22.066064000 9.956925000

H 3.636056000 22.796195000 9.617471000

H 3.865931000 21.309756000 10.567389000

H 5.105445000 22.591320000 10.604523000

C 7.067661000 23.918596000 4.379180000

C 6.982365000 23.787495000 2.974668000

C 8.166811000 23.726084000 2.233672000

H 8.119905000 23.632505000 1.147203000

C 9.408381000 23.784642000 2.859728000

H 10.323339000 23.737370000 2.263509000

C 9.483330000 23.891541000 4.243698000

H 10.461862000 23.917885000 4.729562000

C 8.323213000 23.959594000 5.022464000

C 5.634065000 23.773357000 2.277353000

H 4.917532000 23.297382000 2.966690000

C 5.626058000 22.965238000 0.984262000

H 4.595509000 22.861162000 0.611773000

H 6.209603000 23.455275000 0.188902000

H 6.035610000 21.955316000 1.136545000

C 5.146951000 25.202986000 2.030927000

H 4.153348000 25.199284000 1.555334000

H 5.072291000 25.769724000 2.970435000

H 5.842458000 25.739182000 1.365215000

C 8.432893000 24.006063000 6.535261000

H 7.441400000 24.271341000 6.929478000

C 8.777374000 22.620907000 7.081022000

H 8.048496000 21.868308000 6.743576000

H 9.773286000 22.296400000 6.738854000

H 8.780639000 22.618996000 8.182561000

C 9.415719000 25.064013000 7.029233000

H 9.399928000 25.116176000 8.129030000

H 10.450699000 24.836900000 6.728310000

H 9.161401000 26.060505000 6.636808000

**2:** G = -2860.38540998

In 5.405167000 9.654626000 16.254949000

In 4.140152000 10.586141000 12.941161000

C 6.717892000 10.036553000 14.590091000

H 7.807768000 9.918130000 14.707886000

C 6.246950000 10.383136000 13.372455000

H 6.966940000 10.529757000 12.553318000

C 3.375094000 10.306946000 15.934502000

H 2.671651000 10.336212000 16.783006000

C 2.909727000 10.648926000 14.713412000

H 1.853405000 10.940789000 14.614530000

C 5.966079000 8.576441000 18.037313000

C 5.764660000 7.190749000 18.095114000

C 6.115007000 6.486359000 19.252694000

H 5.967898000 5.404034000 19.297139000

C 6.650873000 7.164241000 20.347115000

H 6.918537000 6.611462000 21.251006000

C 6.850023000 8.543527000 20.292904000

H 7.272018000 9.069953000 21.152948000

C 6.514478000 9.253513000 19.135129000

C 5.190846000 6.509693000 16.895848000

C 6.054479000 6.068525000 15.867544000

C 5.500446000 5.432999000 14.751648000

H 6.152858000 5.077897000 13.951704000

C 4.126615000 5.252862000 14.640971000

H 3.710146000 4.755198000 13.761567000

C 3.279771000 5.725620000 15.637431000

H 2.201200000 5.601834000 15.525734000

C 3.789914000 6.365339000 16.771031000

C 7.558245000 6.240250000 15.980417000

H 7.736095000 7.084609000 16.665496000

C 8.225130000 6.582404000 14.650827000

H 9.283195000 6.839561000 14.814341000

H 8.202729000 5.734107000 13.948368000

H 7.737415000 7.440111000 14.163568000

C 8.192681000 4.999953000 16.612554000

H 9.279844000 5.133805000 16.729698000

H 7.765771000 4.793731000 17.604916000

H 8.024461000 4.112511000 15.981013000

C 2.852672000 6.869055000 17.853276000

H 3.361828000 7.709624000 18.353366000

C 1.528012000 7.394376000 17.305807000

H 1.683322000 8.115810000 16.490136000

H 0.891261000 6.580291000 16.924305000

H 0.963137000 7.897976000 18.105408000

C 2.610033000 5.787537000 18.908232000

H 1.958553000 6.167163000 19.711350000

H 2.118421000 4.910459000 18.456857000

H 3.551391000 5.450600000 19.364814000

C 6.729724000 10.724580000 18.998651000

C 7.963983000 11.201071000 18.501812000

C 8.130122000 12.578304000 18.325045000

H 9.077889000 12.963890000 17.943804000

C 7.100468000 13.465737000 18.618334000

H 7.245737000 14.538891000 18.470598000

C 5.881210000 12.988093000 19.086551000

H 5.076602000 13.693845000 19.301269000

C 5.671580000 11.618651000 19.280688000

C 9.102168000 10.244134000 18.196744000

H 8.649068000 9.262241000 17.984879000

C 9.918657000 10.643356000 16.971250000

H 10.632833000 9.844798000 16.717539000

H 9.275360000 10.816805000 16.095851000

H 10.505527000 11.558667000 17.146732000

C 10.004617000 10.079511000 19.421290000

H 10.811008000 9.356932000 19.218351000

H 10.469241000 11.041481000 19.692164000

H 9.438118000 9.721688000 20.292878000

C 4.349295000 11.110851000 19.827365000

H 4.209458000 10.088715000 19.438200000

C 3.148231000 11.939879000 19.382911000

H 2.213774000 11.437516000 19.676556000

H 3.140864000 12.936072000 19.852597000

H 3.132432000 12.078252000 18.291713000

C 4.404874000 11.014171000 21.353769000

H 3.460405000 10.611752000 21.753096000

H 5.221693000 10.358144000 21.686933000

H 4.566949000 12.009586000 21.797986000

C 3.338083000 10.648636000 10.923017000

C 4.185814000 10.517930000 9.805010000

C 3.659191000 10.543798000 8.507908000

H 4.333982000 10.439635000 7.654065000

C 2.290215000 10.695447000 8.306221000

H 1.884397000 10.714770000 7.291568000

C 1.438935000 10.811383000 9.401359000

H 0.361185000 10.919566000 9.253641000

C 1.952920000 10.785331000 10.703744000

C 5.655837000 10.324207000 9.975121000

C 6.165908000 9.024428000 10.171419000

C 7.547269000 8.856629000 10.310649000

H 7.955778000 7.854335000 10.463747000

C 8.408706000 9.947664000 10.261528000

H 9.485857000 9.800328000 10.373178000

C 7.894900000 11.228170000 10.082838000

H 8.575591000 12.083275000 10.060825000

C 6.519110000 11.437249000 9.939094000

C 5.252247000 7.813748000 10.219554000

H 4.216149000 8.183493000 10.195104000

C 5.418264000 7.020622000 11.513592000

H 5.275255000 7.655648000 12.401793000

H 4.685041000 6.200764000 11.564622000

H 6.422375000 6.574891000 11.595408000

C 5.446453000 6.930788000 8.987440000

H 4.748118000 6.078951000 9.003004000

H 5.270888000 7.499965000 8.061787000

H 6.470465000 6.525848000 8.944163000

C 5.982406000 12.848796000 9.790985000

H 4.902197000 12.769897000 9.597611000

C 6.155044000 13.636701000 11.089536000

H 5.711960000 14.641224000 11.000869000

H 5.668771000 13.126763000 11.936035000

H 7.219447000 13.756696000 11.347496000

C 6.604895000 13.579695000 8.603429000

H 6.450639000 13.019996000 7.668290000

H 6.153732000 14.577053000 8.482698000

H 7.689420000 13.720890000 8.735668000

C 0.998721000 10.872479000 11.848310000

C 0.496275000 9.685206000 12.418902000

C -0.416650000 9.778764000 13.474302000

H -0.816630000 8.866272000 13.924026000

C -0.817317000 11.017073000 13.964992000

H -1.528207000 11.074135000 14.793109000

C -0.301393000 12.183067000 13.407999000

H -0.608304000 13.152370000 13.808990000

C 0.606875000 12.131566000 12.345309000

C 0.910483000 8.320737000 11.899814000

H 1.729123000 8.477702000 11.181292000

C 1.452057000 7.420702000 13.007946000

H 2.281316000 7.899762000 13.550942000

H 0.677072000 7.170880000 13.749971000

H 1.825421000 6.473539000 12.588339000

C -0.237286000 7.654983000 11.141598000

H 0.076909000 6.681587000 10.732565000

H -1.101815000 7.481391000 11.802586000

H -0.575924000 8.284349000 10.304337000

C 1.187989000 13.415472000 11.782422000

H 1.712756000 13.156256000 10.850433000

C 2.225488000 14.002519000 12.739438000

H 2.678707000 14.914508000 12.319958000

H 1.773279000 14.258108000 13.710951000

H 3.038384000 13.285854000 12.937065000

C 0.114310000 14.441400000 11.427868000

H 0.567846000 15.320529000 10.944131000

H -0.629049000 14.016714000 10.736118000

H -0.422557000 14.798857000 12.320817000

**(InTer’)2:** G = -2078.58760713

In 6.118817000 18.573936000 5.158892000

In 5.665166000 21.060256000 3.444782000

C 6.994765000 16.524109000 5.514369000

C 6.532745000 15.438707000 6.273566000

C 7.279530000 14.252311000 6.320503000

H 6.912752000 13.409820000 6.914306000

C 8.481959000 14.140701000 5.624377000

H 9.053857000 13.210437000 5.671289000

C 8.957483000 15.215633000 4.874595000

H 9.903854000 15.138758000 4.331578000

C 8.214822000 16.398052000 4.824034000

C 5.265226000 15.519395000 7.056025000

C 4.053256000 15.097347000 6.476815000

C 2.888995000 15.116368000 7.252572000

H 1.947070000 14.782469000 6.808781000

C 2.918667000 15.554897000 8.573458000

H 2.001963000 15.564105000 9.168125000

C 4.116210000 15.995816000 9.130753000

H 4.139940000 16.353598000 10.163621000

C 5.299952000 15.985960000 8.384745000

C 8.668447000 17.593830000 4.050831000

C 8.329474000 17.719592000 2.686422000

C 8.644130000 18.907068000 2.018419000

H 8.366433000 19.016554000 0.967111000

C 9.276683000 19.954874000 2.681872000

H 9.496463000 20.887613000 2.156828000

C 9.618220000 19.820063000 4.023592000

H 10.116528000 20.640896000 4.542478000

C 9.325929000 18.645118000 4.725959000

C 5.311143000 22.411604000 5.273215000

C 4.206531000 22.217705000 6.124846000

C 3.862175000 23.170077000 7.090751000

H 2.997151000 23.002099000 7.739501000

C 4.622046000 24.331462000 7.225223000

H 4.351370000 25.079203000 7.975191000

C 5.732145000 24.533613000 6.406073000

H 6.339270000 25.437338000 6.516843000

C 6.072877000 23.580955000 5.437560000

C 3.461942000 20.934398000 5.989773000

C 2.588707000 20.730408000 4.902971000

C 2.049591000 19.453444000 4.694114000

H 1.380766000 19.291956000 3.844376000

C 2.347668000 18.402349000 5.554525000

H 1.928186000 17.408320000 5.381609000

C 3.173145000 18.622087000 6.656397000

H 3.389599000 17.803723000 7.345638000

C 3.735250000 19.883111000 6.889533000

C 7.268430000 23.777297000 4.565948000

C 7.130439000 24.412536000 3.315479000

C 8.254743000 24.541696000 2.492527000

H 8.149518000 25.029154000 1.519529000

C 9.496832000 24.065886000 2.902551000

H 10.368820000 24.180629000 2.253671000

C 9.627021000 23.446819000 4.142798000

H 10.604181000 23.079340000 4.468904000

C 8.521048000 23.285765000 4.983957000

C 5.795929000 24.939054000 2.872900000

H 5.862289000 25.404369000 1.879816000

H 5.045293000 24.133169000 2.826814000

H 5.404493000 25.684264000 3.582579000

C 8.661235000 22.585137000 6.304881000

H 8.283942000 23.204142000 7.132625000

H 8.070320000 21.654478000 6.319656000

H 9.710454000 22.330433000 6.510571000

C 2.214867000 21.865594000 3.991170000

H 3.063673000 22.207606000 3.374452000

H 1.406794000 21.570654000 3.307844000

H 1.887043000 22.742752000 4.569007000

C 4.650215000 20.090922000 8.062436000

H 4.232658000 20.816855000 8.776748000

H 4.823478000 19.145322000 8.595606000

H 5.621952000 20.501346000 7.740305000

C 9.693127000 18.518426000 6.177141000

H 8.801064000 18.410829000 6.816537000

H 10.304214000 17.620949000 6.357245000

H 10.251452000 19.400183000 6.519329000

C 7.613941000 16.608708000 1.973914000

H 7.405775000 16.879152000 0.929910000

H 8.204961000 15.679989000 1.981979000

H 6.657529000 16.368756000 2.465909000

C 6.586809000 16.468416000 8.990083000

H 6.443261000 16.777720000 10.034513000

H 7.362004000 15.687342000 8.960538000

H 6.991851000 17.325328000 8.428363000

C 4.010170000 14.642466000 5.046890000

H 4.701813000 13.804678000 4.869815000

H 2.997868000 14.325053000 4.761172000

H 4.322741000 15.450783000 4.366167000

**TS1:** G = -2155.74340269 (i = -180 cm-1)

In 1.088596000 -0.042023000 -0.127786000

In -1.645464000 0.672028000 -0.666130000

C -0.681767000 -1.953622000 -1.574860000

H -0.047774000 -2.735454000 -1.181652000

C -1.538618000 -1.332906000 -2.221435000

H -2.308676000 -1.171152000 -2.964317000

C 3.253128000 0.168617000 -0.619908000

C 4.075687000 -0.952695000 -0.810585000

C 5.407910000 -0.789153000 -1.206843000

H 6.043762000 -1.666646000 -1.356022000

C 5.921296000 0.491053000 -1.414311000

H 6.960841000 0.615963000 -1.728045000

C 5.113175000 1.611699000 -1.225967000

H 5.516829000 2.614577000 -1.392243000

C 3.779998000 1.452924000 -0.830635000

C 3.492981000 -2.305546000 -0.576088000

C 2.732809000 -2.923937000 -1.589645000

C 2.128499000 -4.158610000 -1.322650000

H 1.542172000 -4.644806000 -2.107422000

C 2.269324000 -4.768708000 -0.078930000

H 1.784840000 -5.728264000 0.117769000

C 3.036142000 -4.158787000 0.909975000

H 3.154758000 -4.640307000 1.884538000

C 3.662099000 -2.929910000 0.675517000

C 2.878135000 2.621575000 -0.610520000

C 1.920870000 2.962863000 -1.587166000

C 1.015901000 3.996188000 -1.314789000

H 0.266625000 4.258080000 -2.066347000

C 1.058095000 4.681819000 -0.106241000

H 0.340311000 5.480894000 0.094863000

C 2.019729000 4.351741000 0.845862000

H 2.058281000 4.894837000 1.794295000

C 2.941217000 3.328304000 0.608940000

C -2.908038000 -0.132797000 1.058561000

C -2.455706000 -0.284036000 2.383476000

C -3.336954000 -0.618025000 3.419067000

H -2.958180000 -0.728954000 4.439579000

C -4.690489000 -0.811273000 3.150554000

H -5.379889000 -1.066709000 3.959428000

C -5.158842000 -0.684294000 1.844758000

H -6.217979000 -0.845833000 1.622417000

C -4.277390000 -0.347803000 0.809553000

C -1.001271000 -0.129291000 2.663077000

C -0.182354000 -1.275712000 2.667658000

C 1.202822000 -1.121014000 2.808964000

H 1.841319000 -2.007158000 2.805715000

C 1.768295000 0.148296000 2.947548000

H 2.848556000 0.257227000 3.063772000

C 0.950116000 1.275833000 2.942182000

H 1.390385000 2.270041000 3.043998000

C -0.439014000 1.153286000 2.803490000

C -4.786138000 -0.234340000 -0.589253000

C -4.852804000 -1.387286000 -1.396923000

C -5.275974000 -1.259992000 -2.724294000

H -5.324610000 -2.151688000 -3.355741000

C -5.634552000 -0.018559000 -3.243402000

H -5.961616000 0.066547000 -4.282799000

C -5.584786000 1.112319000 -2.433388000

H -5.874851000 2.086658000 -2.835970000

C -5.166011000 1.022016000 -1.101535000

C 4.498839000 -2.283274000 1.740959000

H 4.417006000 -2.823324000 2.694509000

H 5.561552000 -2.259694000 1.452347000

H 4.199632000 -1.236785000 1.900129000

C 2.580747000 -2.277814000 -2.936988000

H 3.562248000 -2.091372000 -3.399609000

H 1.988167000 -2.908382000 -3.613465000

H 2.084481000 -1.296719000 -2.866666000

C 3.978160000 2.985455000 1.639309000

H 3.999458000 1.904615000 1.843778000

H 4.988767000 3.253609000 1.292805000

H 3.791242000 3.518596000 2.581969000

C 1.836941000 2.223914000 -2.892539000

H 2.823166000 1.885131000 -3.238126000

H 1.197661000 1.326370000 -2.804188000

H 1.382640000 2.854586000 -3.669392000

C -0.771587000 -2.636816000 2.448715000

H -1.322315000 -2.666069000 1.494744000

H 0.010654000 -3.408063000 2.424518000

H -1.497541000 -2.894689000 3.234408000

C -1.302327000 2.378902000 2.762856000

H -2.159297000 2.289385000 3.446685000

H -0.726826000 3.277361000 3.023885000

H -1.724581000 2.531677000 1.755244000

C -5.108640000 2.246918000 -0.235927000

H -5.671992000 2.104984000 0.698832000

H -4.071300000 2.476847000 0.061364000

H -5.513222000 3.123115000 -0.761029000

C -4.470582000 -2.729042000 -0.842702000

H -4.538348000 -3.509209000 -1.613670000

H -3.441757000 -2.716392000 -0.451472000

H -5.122498000 -3.014562000 -0.002516000

**1M:** G = -2155.77319930

In 5.775572000 18.027688000 4.795865000

In 5.675779000 20.762356000 4.599907000

C 4.611358000 18.562980000 2.985204000

H 4.060115000 17.924074000 2.276194000

C 4.729884000 19.892908000 2.792058000

H 4.334884000 20.381904000 1.886798000

C 6.950325000 16.259441000 5.264843000

C 6.513404000 15.110726000 5.934796000

C 7.415354000 14.065295000 6.170758000

H 7.075983000 13.164932000 6.690748000

C 8.740065000 14.169160000 5.746732000

H 9.435612000 13.347068000 5.933656000

C 9.182905000 15.317825000 5.090118000

H 10.223378000 15.401373000 4.763845000

C 8.288921000 16.365098000 4.851189000

C 5.098214000 15.031693000 6.395082000

C 4.091050000 14.628890000 5.495863000

C 2.766603000 14.577909000 5.944410000

H 1.981998000 14.261461000 5.251890000

C 2.443591000 14.916518000 7.255310000

H 1.405539000 14.866425000 7.593572000

C 3.443537000 15.326502000 8.133282000

H 3.189762000 15.605555000 9.159605000

C 4.777131000 15.395394000 7.717825000

C 8.674072000 17.639234000 4.172315000

C 8.402054000 17.803226000 2.796766000

C 8.610369000 19.056913000 2.211439000

H 8.383933000 19.192731000 1.150938000

C 9.087296000 20.125770000 2.963742000

H 9.231209000 21.105345000 2.502026000

C 9.376289000 19.949686000 4.314847000

H 9.750098000 20.791067000 4.903687000

C 9.172012000 18.714651000 4.938036000

C 5.343287000 22.658558000 5.608260000

C 4.331235000 22.596255000 6.580628000

C 4.004848000 23.731305000 7.327712000

H 3.213848000 23.681853000 8.081319000

C 4.694361000 24.924186000 7.106693000

H 4.440337000 25.814630000 7.687270000

C 5.710276000 24.986287000 6.152802000

H 6.251933000 25.922277000 5.989420000

C 6.042737000 23.852649000 5.400320000

C 3.665500000 21.270202000 6.755807000

C 2.628011000 20.891626000 5.876149000

C 2.134711000 19.583712000 5.936642000

H 1.344564000 19.281253000 5.244855000

C 2.646164000 18.668714000 6.851928000

H 2.266963000 17.644510000 6.879976000

C 3.652468000 19.057244000 7.733214000

H 4.053842000 18.336730000 8.450169000

C 4.177641000 20.352725000 7.697456000

C 7.137060000 23.881861000 4.389329000

C 6.829320000 24.091888000 3.030692000

C 7.870023000 24.094100000 2.095329000

H 7.637234000 24.260825000 1.040171000

C 9.188346000 23.893740000 2.494580000

H 9.991918000 23.904522000 1.753820000

C 9.481674000 23.672513000 3.837583000

H 10.515507000 23.502558000 4.150569000

C 8.466124000 23.656162000 4.798939000

C 5.846602000 15.871219000 8.658635000

H 5.420551000 16.155828000 9.630734000

H 6.610776000 15.097429000 8.830021000

H 6.376711000 16.743010000 8.242752000

C 4.431560000 14.263732000 4.079454000

H 5.230208000 13.507552000 4.041032000

H 3.552507000 13.873153000 3.548880000

H 4.805419000 15.138513000 3.522713000

C 9.405682000 18.558790000 6.410950000

H 8.448284000 18.404097000 6.937399000

H 10.031897000 17.683284000 6.636702000

H 9.884345000 19.453495000 6.832215000

C 7.871899000 16.661337000 1.978049000

H 8.476811000 15.753110000 2.116786000

H 6.842538000 16.400565000 2.274437000

H 7.857851000 16.917530000 0.910098000

C 2.081107000 21.866347000 4.873158000

H 2.828756000 22.106368000 4.099367000

H 1.195803000 21.456225000 4.368774000

H 1.808311000 22.819884000 5.349091000

C 5.320324000 20.733941000 8.590462000

H 5.128953000 21.676924000 9.123127000

H 5.525025000 19.946472000 9.328830000

H 6.236566000 20.889792000 7.995456000

C 8.782789000 23.380800000 6.240915000

H 8.477698000 24.215653000 6.890305000

H 8.236670000 22.493690000 6.600383000

H 9.858292000 23.206936000 6.384312000

C 5.408619000 24.305411000 2.593414000

H 5.354756000 24.537601000 1.521019000

H 4.796346000 23.408167000 2.780385000

H 4.934758000 25.125462000 3.154110000

**TS2:** G = -2232.91198104 (i = -275 cm-1)

In 0.072032000 -1.393741000 -0.845016000

In -1.268554000 1.070493000 -0.377607000

C -1.969923000 -1.595449000 -1.587884000

H -2.517393000 -2.398873000 -2.105766000

C -2.588447000 -0.461895000 -1.214096000

H -3.669511000 -0.288642000 -1.333290000

C 1.539115000 -2.954492000 -0.511840000

C 1.320414000 -3.813240000 0.574145000

C 2.193428000 -4.881079000 0.802335000

H 2.024345000 -5.553339000 1.648067000

C 3.277840000 -5.084537000 -0.052579000

H 3.957371000 -5.922357000 0.122909000

C 3.503898000 -4.223603000 -1.126799000

H 4.358872000 -4.384776000 -1.789297000

C 2.635732000 -3.150580000 -1.359442000

C 0.143935000 -3.511098000 1.439377000

C -1.122550000 -4.032896000 1.105147000

C -2.233574000 -3.638880000 1.858119000

H -3.219731000 -4.031361000 1.597198000

C -2.096176000 -2.751130000 2.920374000

H -2.974882000 -2.447407000 3.494304000

C -0.841162000 -2.246566000 3.249787000

H -0.733153000 -1.549833000 4.085153000

C 0.292462000 -2.614132000 2.518168000

C 2.838177000 -2.171871000 -2.464809000

C 2.053866000 -2.255895000 -3.633432000

C 2.193802000 -1.264123000 -4.612230000

H 1.587896000 -1.323931000 -5.520063000

C 3.089750000 -0.213971000 -4.441984000

H 3.183307000 0.555745000 -5.211853000

C 3.867062000 -0.143273000 -3.287874000

H 4.570851000 0.681769000 -3.153214000

C 3.755899000 -1.115475000 -2.288565000

C -1.271142000 2.830593000 0.876901000

C -2.482585000 3.138869000 1.521090000

C -2.576921000 4.266401000 2.344138000

H -3.525450000 4.494724000 2.837883000

C -1.467019000 5.085326000 2.538372000

H -1.541141000 5.964979000 3.182702000

C -0.259158000 4.775382000 1.917525000

H 0.619110000 5.407125000 2.076905000

C -0.151708000 3.651797000 1.088591000

C -3.649513000 2.228160000 1.341455000

C -4.537675000 2.420816000 0.265734000

C -5.591514000 1.517675000 0.091862000

H -6.284576000 1.661839000 -0.741424000

C -5.759986000 0.442798000 0.959880000

H -6.584717000 -0.257959000 0.807946000

C -4.875374000 0.258017000 2.018291000

H -5.004466000 -0.588267000 2.698255000

C -3.814313000 1.144616000 2.227101000

C 1.154453000 3.327295000 0.450325000

C 1.531177000 3.974937000 -0.740673000

C 2.750869000 3.640305000 -1.335972000

H 3.044648000 4.135106000 -2.265640000

C 3.582054000 2.681862000 -0.764188000

H 4.533840000 2.429492000 -1.237990000

C 3.203385000 2.049396000 0.416532000

H 3.860347000 1.304953000 0.873007000

C 1.991804000 2.362252000 1.041090000

C 1.642775000 -2.058795000 2.866476000

H 1.583598000 -1.395215000 3.739726000

H 2.362503000 -2.862375000 3.085453000

H 2.064123000 -1.483959000 2.026562000

C -1.276317000 -4.978959000 -0.049722000

H -0.633459000 -5.864401000 0.069463000

H -2.317666000 -5.313545000 -0.149948000

H -0.978322000 -4.500288000 -0.996197000

C 4.587746000 -1.030585000 -1.042220000

H 3.952532000 -1.026721000 -0.142971000

H 5.257053000 -1.899233000 -0.947758000

H 5.201975000 -0.120123000 -1.037935000

C 1.083449000 -3.385989000 -3.835175000

H 1.530963000 -4.353287000 -3.564064000

H 0.185572000 -3.268168000 -3.205862000

H 0.748455000 -3.431776000 -4.880315000

C -4.342771000 3.563582000 -0.688611000

H -3.384979000 3.470843000 -1.226534000

H -5.149967000 3.599878000 -1.432794000

H -4.309703000 4.528768000 -0.160603000

C -2.854402000 0.935216000 3.362337000

H -2.755085000 1.841650000 3.977954000

H -3.179102000 0.107860000 4.007587000

H -1.844767000 0.698320000 2.989287000

C 1.597006000 1.681422000 2.319437000

H 1.308005000 2.411250000 3.090703000

H 0.725421000 1.023823000 2.166446000

H 2.419379000 1.066396000 2.708888000

C 0.626226000 4.991949000 -1.371992000

H 1.018821000 5.324624000 -2.342935000

H -0.383757000 4.580447000 -1.525600000

H 0.503588000 5.877414000 -0.728727000

C -0.413997000 1.789171000 -2.488893000

H -0.764681000 2.569843000 -3.157181000

C 0.518427000 1.003067000 -2.172107000

H 1.551931000 0.669848000 -2.249318000

**2M:** G = -2232.96900297

In 5.744795000 17.514939000 5.319876000

In 5.333728000 21.063292000 5.298979000

C 4.278764000 18.426755000 4.036062000

H 3.666459000 17.835316000 3.335580000

C 4.225774000 19.773311000 3.964189000

H 3.564801000 20.232942000 3.210328000

C 7.057332000 15.926801000 4.653544000

C 7.098484000 14.625247000 5.166140000

C 8.052757000 13.721000000 4.684822000

H 8.084550000 12.702620000 5.082019000

C 8.963156000 14.119356000 3.705415000

H 9.708898000 13.410019000 3.337622000

C 8.929727000 15.417412000 3.194382000

H 9.646625000 15.726871000 2.428676000

C 7.975272000 16.322317000 3.667592000

C 6.111710000 14.262276000 6.221491000

C 4.836526000 13.791452000 5.850499000

C 3.897165000 13.526316000 6.852757000

H 2.906273000 13.159595000 6.571108000

C 4.210501000 13.725949000 8.194357000

H 3.466171000 13.516162000 8.966646000

C 5.471315000 14.195541000 8.551556000

H 5.717435000 14.355764000 9.604762000

C 6.436190000 14.470591000 7.576695000

C 7.843715000 17.724497000 3.172496000

C 7.104283000 17.975723000 1.995807000

C 6.877683000 19.296950000 1.607686000

H 6.299958000 19.493704000 0.701209000

C 7.345165000 20.357396000 2.377545000

H 7.149175000 21.385999000 2.069857000

C 8.070117000 20.108671000 3.539987000

H 8.468579000 20.941411000 4.124533000

C 8.347554000 18.793799000 3.944150000

C 5.264901000 23.243193000 5.293444000

C 3.997817000 23.717488000 5.682755000

C 3.750405000 25.086276000 5.813132000

H 2.759969000 25.434283000 6.119416000

C 4.772530000 25.998620000 5.558047000

H 4.588184000 27.071148000 5.659294000

C 6.033559000 25.541878000 5.182257000

H 6.839224000 26.256558000 4.991763000

C 6.290343000 24.169613000 5.051327000

C 2.951610000 22.692332000 5.967080000

C 2.069891000 22.288461000 4.942851000

C 1.165372000 21.255484000 5.199822000

H 0.486162000 20.932516000 4.406574000

C 1.137326000 20.623990000 6.440253000

H 0.435192000 19.806475000 6.621115000

C 2.005187000 21.029814000 7.448467000

H 1.980215000 20.537239000 8.423874000

C 2.915748000 22.071781000 7.234868000

C 7.661308000 23.727670000 4.669150000

C 8.047796000 23.742609000 3.315126000

C 9.351789000 23.364733000 2.978000000

H 9.653696000 23.368983000 1.927259000

C 10.257746000 22.976444000 3.960492000

H 11.271841000 22.678563000 3.682956000

C 9.866939000 22.959485000 5.296656000

H 10.576990000 22.654987000 6.070268000

C 8.572429000 23.337261000 5.670788000

C 7.788567000 14.994778000 7.965174000

H 7.890730000 15.057120000 9.057230000

H 8.595360000 14.355648000 7.575466000

H 7.960072000 15.999438000 7.546179000

C 4.490298000 13.589181000 4.402974000

H 5.194042000 12.897189000 3.915303000

H 3.473650000 13.188141000 4.290769000

H 4.552253000 14.536261000 3.843144000

C 9.226279000 18.549352000 5.137620000

H 8.819109000 17.778054000 5.806774000

H 10.218534000 18.195050000 4.813382000

H 9.361162000 19.472115000 5.717206000

C 6.530352000 16.839439000 1.202361000

H 7.307432000 16.124495000 0.893988000

H 5.806875000 16.269263000 1.807637000

H 6.014316000 17.205530000 0.304460000

C 2.131007000 22.936968000 3.591498000

H 3.123752000 22.798625000 3.133281000

H 1.376565000 22.513660000 2.914424000

H 1.970298000 24.023755000 3.658531000

C 3.811058000 22.539214000 8.347190000

H 3.553123000 23.566469000 8.650386000

H 3.719471000 21.886484000 9.225726000

H 4.868279000 22.563195000 8.044360000

C 8.172623000 23.353452000 7.117636000

H 7.866387000 24.363152000 7.433129000

H 7.310976000 22.695758000 7.303803000

H 9.000818000 23.024729000 7.760196000

C 7.074294000 24.156855000 2.248905000

H 7.487824000 23.981113000 1.246098000

H 6.124538000 23.606768000 2.338645000

H 6.819534000 25.225287000 2.330527000

C 6.192534000 20.024216000 7.000088000

H 6.543560000 20.604104000 7.871430000

C 6.259876000 18.678014000 7.063471000

H 6.664974000 18.207362000 7.975174000

**5M:** G = -2345.86983223

In 5.680133000 7.953673000 1.166711000

C 4.274481000 6.797932000 -0.028084000

H 3.189901000 7.002865000 0.053273000

C 4.665967000 5.862833000 -0.920811000

H 3.880152000 5.361483000 -1.516097000

N 5.820913000 9.751902000 -0.319006000

H 6.009687000 9.349713000 -1.239811000

H 6.580389000 10.396907000 -0.106298000

H 4.968799000 10.310024000 -0.394853000

C 4.952704000 8.733307000 3.092905000

C 4.360113000 9.991826000 3.307128000

C 3.884708000 10.358018000 4.573206000

H 3.428138000 11.342539000 4.710892000

C 3.987430000 9.478567000 5.647554000

H 3.613492000 9.767466000 6.633127000

C 4.569975000 8.228394000 5.456485000

H 4.657673000 7.528024000 6.292221000

C 5.047088000 7.858129000 4.193391000

C 5.670794000 6.512509000 4.023186000

C 4.873385000 5.408014000 3.664780000

C 5.479646000 4.153142000 3.530887000

H 4.860938000 3.289909000 3.269381000

C 6.848523000 3.994586000 3.737548000

H 7.308212000 3.008180000 3.632472000

C 7.630842000 5.095041000 4.075383000

H 8.706568000 4.975486000 4.228656000

C 7.056698000 6.360541000 4.227548000

C 4.227303000 10.967567000 2.188101000

C 3.111337000 10.902015000 1.331779000

C 3.013388000 11.816126000 0.275259000

H 2.149619000 11.767179000 -0.393460000

C 3.995859000 12.783742000 0.074538000

H 3.904129000 13.493572000 -0.751389000

C 5.095828000 12.841222000 0.926606000

H 5.871349000 13.595618000 0.767424000

C 5.228888000 11.936806000 1.986328000

In 6.748339000 5.288553000 -1.169628000

C 8.154060000 6.445015000 0.024582000

H 9.238783000 6.240915000 -0.057101000

C 7.762752000 7.380658000 0.916875000

H 8.548882000 7.882662000 1.511164000

N 6.605545000 3.490891000 0.315840000

H 6.414473000 3.892834000 1.236315000

H 5.847351000 2.844423000 0.102818000

H 7.457873000 2.933357000 0.394093000

C 7.477440000 4.507497000 -3.094610000

C 8.069927000 3.248745000 -3.307733000

C 8.547122000 2.882205000 -4.573053000

H 9.003548000 1.897503000 -4.709929000

C 8.446213000 3.761439000 -5.647754000

H 8.821475000 3.472198000 -6.632722000

C 7.863785000 5.011842000 -5.457781000

H 7.777488000 5.712042000 -6.293801000

C 7.384994000 5.382463000 -4.195444000

C 6.761508000 6.728292000 -4.026098000

C 7.558960000 7.832631000 -3.667334000

C 6.952866000 9.087588000 -3.533613000

H 7.571546000 9.950691000 -3.271629000

C 5.584162000 9.246462000 -3.741244000

H 5.124679000 10.232995000 -3.636479000

C 4.801834000 8.146196000 -4.079685000

H 3.726246000 8.266000000 -4.233759000

C 5.375780000 6.880547000 -4.231402000

C 8.201003000 2.273147000 -2.188354000

C 9.315528000 2.338986000 -1.330201000

C 9.411822000 1.425128000 -0.273313000

H 10.274443000 1.474372000 0.396863000

C 8.429135000 0.457461000 -0.073998000

H 8.519448000 -0.252067000 0.752340000

C 7.330630000 0.399610000 -0.927956000

H 6.554977000 -0.354895000 -0.769937000

C 7.199220000 1.303786000 -1.988092000

C 6.438214000 11.974339000 2.875274000

H 6.158150000 12.120220000 3.929566000

H 6.985270000 11.018580000 2.833312000

H 7.123432000 12.781736000 2.582177000

C 2.053579000 9.857513000 1.538190000

H 1.243003000 9.963020000 0.804274000

H 2.475215000 8.844520000 1.446275000

H 1.622053000 9.922219000 2.548767000

C 7.901830000 7.542294000 4.601783000

H 7.584493000 7.970201000 5.565564000

H 8.962089000 7.264283000 4.675925000

H 7.803862000 8.346407000 3.856483000

C 3.399345000 5.570173000 3.433413000

H 3.208019000 6.239049000 2.579721000

H 2.924029000 4.602864000 3.220096000

H 2.902938000 6.020519000 4.306355000

C 9.032927000 7.670231000 -3.435660000

H 9.224159000 6.999586000 -2.583372000

H 9.529791000 7.221931000 -4.309408000

H 9.507952000 8.637211000 -3.220185000

C 4.530588000 5.698992000 -4.606131000

H 4.849366000 5.270122000 -5.569006000

H 4.626775000 4.895384000 -3.860053000

H 3.470630000 5.977585000 -4.682369000

C 10.373267000 3.383900000 -1.534745000

H 11.184089000 3.276808000 -0.801318000

H 10.804330000 3.321601000 -2.545624000

H 9.951585000 4.396662000 -1.440164000

C 5.991601000 1.265543000 -2.879298000

H 6.273752000 1.117825000 -3.932803000

H 5.305502000 0.458823000 -2.586403000

H 5.444853000 2.221578000 -2.840054000

**TS1‘:** G = -2345.81878818 (i = -1550 cm-1)

In 1.806221000 0.031247000 0.001873000

C 0.818295000 -0.346832000 -1.895856000

H 1.411898000 -0.530071000 -2.809571000

C -0.519091000 -0.470860000 -1.996584000

H -0.952238000 -0.745063000 -2.975239000

N 2.154958000 -2.231544000 0.462204000

H 1.260374000 -2.707237000 0.325019000

H 2.457367000 -2.420259000 1.416863000

H 2.838174000 -2.676903000 -0.152795000

C 3.767386000 1.012769000 0.051299000

C 5.012055000 0.354719000 0.079332000

C 6.209633000 1.081731000 0.087092000

H 7.162187000 0.544350000 0.108968000

C 6.192041000 2.473115000 0.063822000

H 7.128803000 3.036173000 0.070141000

C 4.971488000 3.140453000 0.026677000

H 4.941880000 4.233504000 0.000430000

C 3.769670000 2.421050000 0.018030000

C 2.491933000 3.189436000 -0.046861000

C 1.949084000 3.527757000 -1.302336000

C 0.793834000 4.315885000 -1.348529000

H 0.374589000 4.588937000 -2.320892000

C 0.183854000 4.755428000 -0.177047000

H -0.718605000 5.369710000 -0.226925000

C 0.711195000 4.394489000 1.058791000

H 0.220384000 4.721317000 1.979190000

C 1.865781000 3.611455000 1.142922000

C 5.097839000 -1.133085000 0.092066000

C 5.066728000 -1.844404000 -1.123086000

C 5.145014000 -3.242555000 -1.091160000

H 5.120520000 -3.799081000 -2.032052000

C 5.261337000 -3.924173000 0.118523000

H 5.326566000 -5.015067000 0.127634000

C 5.292053000 -3.212195000 1.314968000

H 5.378290000 -3.745114000 2.265958000

C 5.207994000 -1.815181000 1.319091000

In -1.933515000 -0.006550000 -0.435121000

C -0.872980000 0.621761000 1.650323000

H -1.455664000 0.516281000 2.586994000

C 0.406290000 0.164710000 1.676158000

H 0.769259000 -0.281615000 2.623400000

N -1.919763000 2.192097000 -0.277550000

H -1.325466000 1.626242000 0.795101000

H -1.313727000 2.703534000 -0.918181000

H -2.777547000 2.726547000 -0.141543000

C -3.776638000 -1.027878000 0.108491000

C -4.993396000 -0.353114000 0.317293000

C -6.152893000 -1.068212000 0.641807000

H -7.089850000 -0.526318000 0.797943000

C -6.116798000 -2.454702000 0.764053000

H -7.025459000 -3.007492000 1.015488000

C -4.917630000 -3.133393000 0.565320000

H -4.877981000 -4.222141000 0.660694000

C -3.752414000 -2.428608000 0.238975000

C -2.485306000 -3.189943000 0.037908000

C -1.673081000 -3.494599000 1.147782000

C -0.512865000 -4.250870000 0.946774000

H 0.110064000 -4.507240000 1.808451000

C -0.153493000 -4.683162000 -0.328534000

H 0.754690000 -5.274817000 -0.470830000

C -0.950439000 -4.357063000 -1.421762000

H -0.665597000 -4.687276000 -2.424114000

C -2.125240000 -3.616985000 -1.255478000

C -5.069786000 1.131038000 0.202376000

C -4.929488000 1.922295000 1.358660000

C -4.988917000 3.314618000 1.236261000

H -4.872708000 3.933684000 2.129912000

C -5.186624000 3.913193000 -0.005395000

H -5.229874000 5.002141000 -0.087225000

C -5.323958000 3.122284000 -1.143097000

H -5.473931000 3.590868000 -2.119464000

C -5.266206000 1.727313000 -1.057324000

C 5.199612000 -1.053330000 2.612541000

H 6.027714000 -0.329860000 2.658167000

H 4.273860000 -0.464188000 2.716069000

H 5.281067000 -1.729676000 3.474635000

C 4.935577000 -1.118205000 -2.430405000

H 4.989399000 -1.814965000 -3.278031000

H 3.978794000 -0.575639000 -2.490542000

H 5.726288000 -0.361682000 -2.547053000

C 2.430671000 3.223161000 2.477718000

H 3.464312000 3.582930000 2.598232000

H 1.821776000 3.627410000 3.297992000

H 2.464157000 2.127340000 2.582549000

C 2.603131000 3.058534000 -2.569847000

H 2.628599000 1.958461000 -2.616091000

H 2.062719000 3.425342000 -3.453260000

H 3.648106000 3.399102000 -2.632667000

C -2.046583000 -3.020027000 2.521571000

H -2.105428000 -1.920935000 2.553123000

H -3.036298000 -3.396908000 2.821355000

H -1.309411000 -3.346827000 3.267914000

C -2.989682000 -3.284362000 -2.435989000

H -4.012860000 -3.668627000 -2.303180000

H -3.083466000 -2.194870000 -2.566466000

H -2.575330000 -3.705953000 -3.361837000

C -4.698368000 1.283527000 2.697098000

H -4.562309000 2.042768000 3.479413000

H -5.540935000 0.635805000 2.984808000

H -3.804254000 0.641518000 2.678384000

C -5.381801000 0.883979000 -2.294346000

H -6.126344000 0.083401000 -2.170404000

H -5.662343000 1.493713000 -3.164085000

H -4.426134000 0.384936000 -2.526311000

**INT1:** G = -2345.85486281

In 5.536096000 7.801641000 0.967570000

C 3.916417000 7.156390000 -0.310276000

H 2.940726000 7.669799000 -0.346129000

C 4.117456000 6.116900000 -1.140250000

H 3.296385000 5.828570000 -1.820919000

N 6.336286000 9.467836000 -0.439081000

H 6.181289000 9.107872000 -1.383270000

H 7.330847000 9.668495000 -0.342050000

H 5.842284000 10.359028000 -0.372986000

C 5.251264000 8.622343000 2.977697000

C 4.904428000 9.970810000 3.196416000

C 4.638568000 10.446778000 4.485403000

H 4.369795000 11.497731000 4.625630000

C 4.706634000 9.588590000 5.579194000

H 4.492743000 9.959275000 6.584841000

C 5.041010000 8.253166000 5.380715000

H 5.089127000 7.567087000 6.231078000

C 5.310581000 7.765455000 4.093967000

C 5.642321000 6.315394000 3.965110000

C 4.647728000 5.393932000 3.580351000

C 4.967869000 4.030613000 3.543350000

H 4.204520000 3.313162000 3.232678000

C 6.236585000 3.583674000 3.896347000

H 6.468610000 2.516600000 3.861866000

C 7.215772000 4.501246000 4.267125000

H 8.220417000 4.155112000 4.524745000

C 6.938934000 5.870707000 4.295917000

C 4.802375000 10.916264000 2.049683000

C 3.631816000 10.934564000 1.265715000

C 3.573990000 11.788968000 0.158205000

H 2.673362000 11.798101000 -0.461441000

C 4.641248000 12.628308000 -0.153108000

H 4.580338000 13.292281000 -1.018997000

C 5.784926000 12.622388000 0.643484000

H 6.623551000 13.280611000 0.400667000

C 5.884053000 11.766106000 1.746355000

In 5.867634000 4.834158000 -1.234964000

C 7.389869000 5.239448000 1.001138000

H 8.274380000 4.617590000 0.815618000

C 7.237052000 6.483335000 0.499073000

H 8.060464000 6.850270000 -0.135877000

N 5.390931000 3.064240000 -0.237733000

H 6.632425000 4.779072000 1.640308000

H 4.398393000 2.844124000 -0.308659000

H 5.913019000 2.238338000 -0.521291000

C 7.318962000 4.789906000 -2.894666000

C 8.326424000 3.806639000 -2.988825000

C 9.171707000 3.742977000 -4.103435000

H 9.941398000 2.966987000 -4.144988000

C 9.032303000 4.648606000 -5.150243000

H 9.687867000 4.590239000 -6.022777000

C 8.048468000 5.628038000 -5.074632000

H 7.925125000 6.346654000 -5.889992000

C 7.201556000 5.706882000 -3.960078000

C 6.181959000 6.795866000 -3.959798000

C 6.579743000 8.115851000 -3.668467000

C 5.625584000 9.138851000 -3.721913000

H 5.929780000 10.168486000 -3.510471000

C 4.300772000 8.863360000 -4.053144000

H 3.566196000 9.671571000 -4.087623000

C 3.920145000 7.559448000 -4.347920000

H 2.882679000 7.339506000 -4.613298000

C 4.848846000 6.513973000 -4.311594000

C 8.528074000 2.798105000 -1.909962000

C 9.460490000 3.050338000 -0.886706000

C 9.630698000 2.097309000 0.123456000

H 10.343187000 2.294598000 0.929249000

C 8.907746000 0.908494000 0.109621000

H 9.047416000 0.174040000 0.906683000

C 8.011650000 0.652444000 -0.924754000

H 7.451513000 -0.286470000 -0.943036000

C 7.812066000 1.586351000 -1.946846000

C 7.139919000 11.721901000 2.567270000

H 6.943192000 11.978350000 3.619209000

H 7.569144000 10.707058000 2.575652000

H 7.895124000 12.418131000 2.177095000

C 2.459103000 10.068124000 1.623029000

H 1.639004000 10.195037000 0.903378000

H 2.733940000 9.003460000 1.650780000

H 2.083071000 10.316747000 2.627783000

C 8.016342000 6.855343000 4.645819000

H 7.756984000 7.448432000 5.536020000

H 8.970636000 6.345040000 4.836151000

H 8.167031000 7.575246000 3.825098000

C 3.260117000 5.848214000 3.225482000

H 3.232359000 6.305219000 2.221874000

H 2.562444000 4.999616000 3.216000000

H 2.888853000 6.604296000 3.932769000

C 8.006954000 8.422880000 -3.309180000

H 8.355053000 7.789026000 -2.478075000

H 8.685985000 8.221724000 -4.151670000

H 8.127938000 9.477813000 -3.023884000

C 4.427885000 5.113612000 -4.649559000

H 5.055731000 4.689548000 -5.448223000

H 4.538074000 4.439058000 -3.784868000

H 3.378152000 5.081392000 -4.971343000

C 10.274552000 4.313071000 -0.892279000

H 10.844921000 4.422917000 0.040673000

H 10.988029000 4.317153000 -1.731887000

H 9.640296000 5.202770000 -1.024748000

C 6.860030000 1.300439000 -3.071960000

H 7.398124000 1.205569000 -4.028770000

H 6.304240000 0.369092000 -2.895868000

H 6.137769000 2.120419000 -3.202660000

**TS2‘:** G = -2345.80918226 (i = -1115 cm-1)

In -1.360672000 1.173006000 1.253961000

C -2.369899000 -0.267027000 0.086120000

H -3.469137000 -0.302220000 0.111333000

C -1.681956000 -1.156736000 -0.651081000

H -2.316079000 -1.890037000 -1.184879000

N 0.507489000 1.512817000 0.255051000

H 0.302550000 1.840047000 -0.693658000

H 1.212980000 0.400810000 0.079517000

H 1.105223000 2.216891000 0.687926000

C -1.513116000 2.235749000 3.109160000

C -1.661308000 3.628273000 3.130003000

C -1.766937000 4.289362000 4.357797000

H -1.884555000 5.376044000 4.383840000

C -1.721617000 3.559812000 5.546624000

H -1.806280000 4.080174000 6.503934000

C -1.566716000 2.173248000 5.525345000

H -1.526509000 1.609295000 6.461062000

C -1.462066000 1.503511000 4.302008000

C -1.283417000 0.027546000 4.184561000

C -2.411507000 -0.794609000 3.981928000

C -2.218996000 -2.163300000 3.769876000

H -3.088017000 -2.804707000 3.603304000

C -0.940135000 -2.711273000 3.765779000

H -0.805417000 -3.782610000 3.598002000

C 0.167879000 -1.894189000 3.972052000

H 1.171228000 -2.327877000 3.973908000

C 0.017346000 -0.518371000 4.176867000

C -1.680621000 4.330560000 1.815853000

C -2.873445000 4.370147000 1.064186000

C -2.845636000 4.939427000 -0.214253000

H -3.767735000 4.976462000 -0.800387000

C -1.665899000 5.458720000 -0.738297000

H -1.658756000 5.894941000 -1.740235000

C -0.494450000 5.419914000 0.013172000

H 0.432972000 5.826119000 -0.399617000

C -0.482948000 4.860099000 1.294327000

In 0.400033000 -1.813660000 -0.942230000

C 2.705285000 -1.134759000 1.094360000

H 3.771748000 -1.363759000 1.230785000

C 2.215028000 -0.523810000 0.000979000

H 2.957221000 -0.253512000 -0.766613000

N 0.290958000 -3.606416000 0.135491000

H 2.046313000 -1.455283000 1.910917000

H 0.016071000 -3.415772000 1.099814000

H 1.178691000 -4.105824000 0.182001000

C 1.287982000 -1.828920000 -2.956416000

C 2.187955000 -2.842392000 -3.344283000

C 2.776973000 -2.829958000 -4.615638000

H 3.467235000 -3.632084000 -4.892273000

C 2.484646000 -1.815032000 -5.521280000

H 2.945022000 -1.810945000 -6.512617000

C 1.595011000 -0.808989000 -5.156666000

H 1.348905000 -0.010433000 -5.862575000

C 1.000592000 -0.810036000 -3.888085000

C 0.039603000 0.286063000 -3.568919000

C 0.517811000 1.547021000 -3.163022000

C -0.403667000 2.561137000 -2.874053000

H -0.040338000 3.540548000 -2.549446000

C -1.772855000 2.336576000 -2.995009000

H -2.478773000 3.135728000 -2.756401000

C -2.234745000 1.096195000 -3.421815000

H -3.308537000 0.917629000 -3.522170000

C -1.342949000 0.060185000 -3.714960000

C 2.518868000 -3.971860000 -2.426811000

C 3.699999000 -3.936373000 -1.660116000

C 3.978928000 -5.001238000 -0.797303000

H 4.889560000 -4.971815000 -0.192432000

C 3.119417000 -6.093416000 -0.710671000

H 3.351334000 -6.920378000 -0.034620000

C 1.967132000 -6.131017000 -1.489193000

H 1.288791000 -6.985665000 -1.422287000

C 1.648439000 -5.075652000 -2.349512000

C 0.785450000 4.813881000 2.096429000

H 0.709035000 5.436278000 3.001785000

H 1.003447000 3.792878000 2.447637000

H 1.640710000 5.172216000 1.507216000

C -4.157189000 3.831307000 1.630171000

H -4.990562000 3.983313000 0.931123000

H -4.090564000 2.754231000 1.852256000

H -4.406695000 4.323272000 2.582873000

C 1.218811000 0.360770000 4.375798000

H 1.181253000 0.879733000 5.345951000

H 2.147441000 -0.223967000 4.331282000

H 1.273080000 1.146367000 3.605119000

C -3.793396000 -0.207913000 3.969245000

H -3.940866000 0.446752000 3.094107000

H -4.556901000 -0.996473000 3.931634000

H -3.974748000 0.416868000 4.856440000

C 1.992629000 1.807370000 -3.049345000

H 2.497762000 1.008036000 -2.488942000

H 2.467859000 1.838356000 -4.042725000

H 2.189711000 2.766904000 -2.550247000

C -1.849773000 -1.270214000 -4.188973000

H -1.466748000 -1.503057000 -5.195040000

H -1.507171000 -2.085561000 -3.534172000

H -2.947721000 -1.288529000 -4.220379000

C 4.649830000 -2.778912000 -1.767224000

H 5.501140000 -2.902854000 -1.083421000

H 5.042041000 -2.675196000 -2.790678000

H 4.146965000 -1.830670000 -1.527589000

C 0.384197000 -5.109761000 -3.156268000

H 0.578920000 -4.924611000 -4.223599000

H -0.124111000 -6.078218000 -3.053310000

H -0.311365000 -4.324692000 -2.819168000

**INT2‘:** G = -2345.83117845

In 5.839426000 8.644880000 0.737172000

C 4.428559000 7.721099000 -0.621309000

H 3.624339000 8.449522000 -0.830287000

C 4.304636000 6.533682000 -1.231684000

H 3.416646000 6.409602000 -1.876128000

N 6.959603000 10.158644000 -0.185921000

H 6.343791000 10.642257000 -0.842222000

H 8.858771000 7.559808000 1.860016000

H 7.238579000 10.876406000 0.483095000

C 5.398216000 8.705406000 2.886085000

C 4.652757000 9.843254000 3.258540000

C 4.241588000 10.038475000 4.580136000

H 3.662857000 10.929084000 4.840300000

C 4.555293000 9.091765000 5.552118000

H 4.234076000 9.236964000 6.586600000

C 5.262095000 7.947718000 5.194496000

H 5.488543000 7.187386000 5.947341000

C 5.679267000 7.742529000 3.871098000

C 6.388783000 6.471108000 3.552071000

C 5.683824000 5.408057000 2.952032000

C 6.363995000 4.214160000 2.681372000

H 5.830763000 3.394353000 2.194942000

C 7.710555000 4.071858000 3.001403000

H 8.225111000 3.135429000 2.778173000

C 8.397032000 5.122710000 3.602433000

H 9.455789000 5.012435000 3.852593000

C 7.748327000 6.327313000 3.892552000

C 4.260356000 10.809781000 2.193292000

C 3.138843000 10.515731000 1.389846000

C 2.830383000 11.371093000 0.325641000

H 1.967872000 11.144171000 -0.306606000

C 3.599607000 12.503581000 0.076069000

H 3.347487000 13.162082000 -0.758890000

C 4.686840000 12.801139000 0.893522000

H 5.289594000 13.692271000 0.698643000

C 5.033739000 11.962445000 1.956188000

In 5.538012000 4.778241000 -1.361542000

C 7.718488000 6.702619000 0.290430000

H 7.457748000 6.739596000 -0.772858000

C 8.555851000 7.610757000 0.811786000

H 8.948461000 8.429667000 0.204208000

N 4.745375000 3.136737000 -0.440224000

H 7.326398000 5.879283000 0.897226000

H 4.108907000 3.277102000 0.338727000

H 5.315974000 2.310892000 -0.281063000

C 7.186723000 4.499739000 -2.749592000

C 8.031321000 3.374407000 -2.702008000

C 9.080586000 3.239391000 -3.619400000

H 9.725648000 2.357740000 -3.569122000

C 9.298711000 4.212836000 -4.590263000

H 10.118665000 4.101171000 -5.304258000

C 8.463371000 5.323824000 -4.653894000

H 8.620910000 6.086591000 -5.421112000

C 7.409570000 5.474757000 -3.744119000

C 6.521644000 6.667716000 -3.866427000

C 6.987198000 7.940009000 -3.481472000

C 6.125267000 9.036930000 -3.592402000

H 6.479811000 10.020271000 -3.272877000

C 4.840070000 8.891085000 -4.100209000

H 4.181576000 9.759319000 -4.184029000

C 4.395985000 7.636155000 -4.503459000

H 3.389483000 7.517724000 -4.914122000

C 5.219027000 6.512997000 -4.388376000

C 7.816905000 2.293686000 -1.697874000

C 8.518987000 2.319282000 -0.477752000

C 8.295955000 1.296374000 0.449970000

H 8.842730000 1.305118000 1.396668000

C 7.400305000 0.266535000 0.175967000

H 7.234952000 -0.524967000 0.911180000

C 6.724028000 0.240234000 -1.040568000

H 6.025530000 -0.571336000 -1.261188000

C 6.922831000 1.245987000 -1.992759000

C 6.228253000 12.262155000 2.813494000

H 5.940125000 12.419323000 3.864484000

H 6.936753000 11.418374000 2.811794000

H 6.754500000 13.160163000 2.461713000

C 2.271125000 9.325542000 1.686626000

H 1.459844000 9.235835000 0.951356000

H 2.843879000 8.385965000 1.680474000

H 1.823246000 9.408984000 2.689423000

C 8.496063000 7.450396000 4.553941000

H 8.168106000 7.589732000 5.596366000

H 9.577238000 7.252855000 4.564501000

H 8.317170000 8.410353000 4.045584000

C 4.230425000 5.542489000 2.599622000

H 4.094787000 6.148601000 1.688781000

H 3.781110000 4.557773000 2.411910000

H 3.665607000 6.039540000 3.401713000

C 8.382469000 8.162282000 -2.967983000

H 8.812447000 7.255120000 -2.522243000

H 9.058729000 8.464042000 -3.785382000

H 8.382290000 8.965743000 -2.216070000

C 4.702023000 5.169383000 -4.822738000

H 5.478853000 4.568195000 -5.316363000

H 4.338456000 4.568936000 -3.969191000

H 3.851936000 5.282920000 -5.509883000

C 9.495045000 3.420465000 -0.182108000

H 9.947641000 3.295973000 0.810888000

H 10.302047000 3.449775000 -0.930439000

H 9.009591000 4.407404000 -0.212931000

C 6.183765000 1.214720000 -3.298933000

H 6.877324000 1.256438000 -4.152697000

H 5.575522000 0.304178000 -3.386642000

H 5.514394000 2.083569000 -3.398275000

**INT2:** G = -2345.86609608

In 5.625586000 8.243920000 0.900646000

C 4.393491000 8.295934000 -0.917214000

H 3.519884000 8.949991000 -1.081206000

C 4.847284000 7.540654000 -1.934845000

H 4.372146000 7.557510000 -2.929265000

N 5.833956000 10.782089000 0.547758000

H 6.170678000 10.897659000 -0.407197000

H 6.413839000 11.369501000 1.145102000

H 4.888068000 11.163500000 0.577258000

C 5.172933000 8.438987000 3.036567000

C 3.998179000 9.097876000 3.456303000

C 3.672558000 9.199933000 4.814787000

H 2.750227000 9.709217000 5.108623000

C 4.513199000 8.661750000 5.783937000

H 4.259865000 8.746545000 6.843815000

C 5.678533000 8.012272000 5.390042000

H 6.348735000 7.584086000 6.141024000

C 6.006052000 7.891197000 4.032060000

C 7.254434000 7.145134000 3.696990000

C 7.234053000 5.735739000 3.707298000

C 8.417582000 5.038337000 3.443647000

H 8.403595000 3.945678000 3.447273000

C 9.606129000 5.719217000 3.192600000

H 10.525271000 5.161676000 2.995229000

C 9.618781000 7.110197000 3.191264000

H 10.549447000 7.647908000 2.991029000

C 8.449876000 7.839470000 3.435393000

C 3.078147000 9.723732000 2.463541000

C 2.198921000 8.925729000 1.706522000

C 1.363776000 9.542473000 0.768037000

H 0.681125000 8.924759000 0.178301000

C 1.395001000 10.919675000 0.576968000

H 0.741930000 11.384972000 -0.165567000

C 2.254210000 11.705213000 1.341599000

H 2.272799000 12.789923000 1.202531000

C 3.093430000 11.124229000 2.298244000

In 6.457265000 6.195187000 -1.404243000

C 8.572655000 7.208756000 0.020248000

H 9.542141000 7.239576000 -0.493580000

C 7.635287000 8.175867000 -0.104258000

H 7.903168000 8.989522000 -0.798753000

N 5.770687000 5.881468000 0.625584000

H 8.437367000 6.361412000 0.703974000

H 4.891767000 5.363698000 0.651860000

H 6.416010000 5.366285000 1.223618000

C 7.615497000 4.730514000 -2.507900000

C 7.547108000 3.362108000 -2.199881000

C 8.228959000 2.428989000 -2.991488000

H 8.166426000 1.365657000 -2.743827000

C 8.976921000 2.850225000 -4.088231000

H 9.502506000 2.117038000 -4.705272000

C 9.054561000 4.206764000 -4.398831000

H 9.642558000 4.542299000 -5.257493000

C 8.378336000 5.146696000 -3.614749000

C 8.459066000 6.604609000 -3.913571000

C 9.574685000 7.344509000 -3.475631000

C 9.577723000 8.731424000 -3.656898000

H 10.434155000 9.312837000 -3.304534000

C 8.507888000 9.372351000 -4.275243000

H 8.520428000 10.457621000 -4.403628000

C 7.427664000 8.628625000 -4.741068000

H 6.595144000 9.127850000 -5.243795000

C 7.388938000 7.240521000 -4.575341000

C 6.747240000 2.913743000 -1.025126000

C 7.392266000 2.686371000 0.206138000

C 6.623781000 2.310488000 1.311551000

H 7.120213000 2.127370000 2.267966000

C 5.242270000 2.172878000 1.207770000

H 4.653811000 1.884171000 2.082263000

C 4.612971000 2.402996000 -0.011347000

H 3.528558000 2.291791000 -0.096837000

C 5.351466000 2.771359000 -1.141585000

C 3.993910000 11.989523000 3.135469000

H 3.609261000 12.086241000 4.163429000

H 5.002090000 11.558647000 3.225635000

H 4.074506000 13.001395000 2.713101000

C 2.149965000 7.436541000 1.892268000

H 1.249101000 7.012858000 1.427265000

H 3.023274000 6.950440000 1.427162000

H 2.162728000 7.161454000 2.957100000

C 8.472640000 9.339319000 3.402245000

H 7.994721000 9.772535000 4.293769000

H 9.501713000 9.718306000 3.334095000

H 7.913902000 9.714186000 2.529067000

C 5.962623000 4.993616000 4.003982000

H 5.149216000 5.327070000 3.341729000

H 6.096056000 3.911422000 3.872628000

H 5.623329000 5.178121000 5.034943000

C 10.752300000 6.658356000 -2.844406000

H 10.438351000 5.893144000 -2.119694000

H 11.356673000 6.136897000 -3.604686000

H 11.406294000 7.380662000 -2.335680000

C 6.239427000 6.440783000 -5.117256000

H 6.590970000 5.683623000 -5.835610000

H 5.710937000 5.890629000 -4.323690000

H 5.511738000 7.089318000 -5.623734000

C 8.875463000 2.877606000 0.338394000

H 9.219401000 2.623124000 1.350155000

H 9.430830000 2.260810000 -0.383901000

H 9.156237000 3.924016000 0.135898000

C 4.654737000 3.028535000 -2.448034000

H 5.174822000 2.543926000 -3.287295000

H 3.617943000 2.666506000 -2.418998000

H 4.620957000 4.105758000 -2.684814000

**INT3:** G = -2345.89069056

In 5.399216000 7.892057000 1.286110000

C 4.401088000 8.025882000 -0.610383000

H 3.364674000 8.383252000 -0.709956000

C 5.137283000 7.719059000 -1.696644000

H 4.662713000 7.843084000 -2.686823000

N 6.813678000 9.712073000 0.900665000

H 7.623609000 9.399657000 0.348486000

H 7.171216000 10.205570000 1.716875000

H 6.313837000 10.389784000 0.324895000

C 4.925809000 8.105201000 3.397991000

C 3.648147000 8.585773000 3.739190000

C 3.302875000 8.797572000 5.079239000

H 2.304495000 9.168999000 5.326285000

C 4.225988000 8.539884000 6.089615000

H 3.955672000 8.709602000 7.134953000

C 5.493673000 8.061555000 5.765745000

H 6.220283000 7.851409000 6.555564000

C 5.845755000 7.839268000 4.429604000

C 7.188952000 7.288397000 4.091651000

C 7.347367000 5.890261000 4.010344000

C 8.578567000 5.370937000 3.601209000

H 8.703208000 4.287438000 3.525225000

C 9.637403000 6.217587000 3.279687000

H 10.589768000 5.800115000 2.943777000

C 9.483486000 7.595807000 3.397029000

H 10.320493000 8.259879000 3.164891000

C 8.267370000 8.148719000 3.817660000

C 2.662539000 8.873973000 2.657893000

C 1.856690000 7.835037000 2.153094000

C 0.959692000 8.119007000 1.118423000

H 0.331047000 7.316415000 0.723622000

C 0.865313000 9.402013000 0.587778000

H 0.161889000 9.608388000 -0.222670000

C 1.674554000 10.419943000 1.083850000

H 1.607600000 11.426489000 0.662093000

C 2.579746000 10.173229000 2.120931000

In 7.167183000 6.886114000 -1.508870000

C 9.702847000 8.469640000 -0.441491000

H 10.532287000 9.191878000 -0.477981000

C 8.816540000 8.328658000 -1.440150000

H 8.967301000 8.980282000 -2.313493000

N 6.967472000 6.498828000 0.691855000

H 9.662918000 7.860472000 0.472380000

H 6.672493000 5.536578000 0.866758000

H 7.825520000 6.619076000 1.231247000

C 7.532151000 5.042947000 -2.658826000

C 7.000396000 3.792581000 -2.289313000

C 7.217601000 2.655871000 -3.078116000

H 6.791670000 1.696937000 -2.768533000

C 7.963349000 2.744387000 -4.250644000

H 8.128890000 1.856285000 -4.865944000

C 8.489743000 3.973861000 -4.637630000

H 9.069705000 4.057880000 -5.561327000

C 8.276320000 5.114209000 -3.852686000

C 8.832841000 6.420459000 -4.309489000

C 10.165476000 6.759404000 -4.007131000

C 10.666796000 7.990136000 -4.441625000

H 11.700913000 8.256638000 -4.206490000

C 9.862683000 8.877620000 -5.150698000

H 10.265697000 9.838652000 -5.480539000

C 8.542917000 8.539999000 -5.437229000

H 7.909427000 9.234146000 -5.996446000

C 8.012641000 7.310951000 -5.029488000

C 6.184455000 3.664416000 -1.048417000

C 6.792983000 3.230640000 0.145701000

C 6.016661000 3.135936000 1.305383000

H 6.483960000 2.794196000 2.231914000

C 4.668127000 3.485541000 1.292343000

H 4.075724000 3.416914000 2.208264000

C 4.075863000 3.917494000 0.109453000

H 3.017497000 4.191097000 0.095188000

C 4.816183000 3.995946000 -1.076103000

C 3.460369000 11.272839000 2.642706000

H 3.290604000 11.451187000 3.715688000

H 4.526361000 11.013195000 2.540564000

H 3.281669000 12.213100000 2.103279000

C 1.975145000 6.440356000 2.697190000

H 1.210018000 5.781098000 2.264657000

H 2.962696000 6.007421000 2.467443000

H 1.875955000 6.422115000 3.793004000

C 8.146589000 9.632563000 4.019808000

H 8.371590000 9.898948000 5.066074000

H 8.857478000 10.183057000 3.385895000

H 7.126240000 9.993713000 3.827158000

C 6.203879000 4.980339000 4.348512000

H 5.382970000 5.090826000 3.622992000

H 6.520164000 3.928768000 4.349972000

H 5.782613000 5.219727000 5.336122000

C 11.020654000 5.830233000 -3.197252000

H 10.582335000 5.677871000 -2.197707000

H 11.095543000 4.835875000 -3.662773000

H 12.034572000 6.234166000 -3.069753000

C 6.595308000 6.944346000 -5.367643000

H 6.553371000 6.008068000 -5.945561000

H 5.993666000 6.772891000 -4.461586000

H 6.111357000 7.737314000 -5.954369000

C 8.260539000 2.917831000 0.182985000

H 8.571545000 2.591288000 1.185171000

H 8.525517000 2.131174000 -0.539249000

H 8.854421000 3.804206000 -0.093132000

C 4.160260000 4.414326000 -2.358085000

H 4.235611000 3.619219000 -3.116394000

H 3.098969000 4.650997000 -2.201560000

H 4.650899000 5.303455000 -2.779928000

**TS3:** G = -2345.85916151 (i = -1007 cm-1)

In -1.714203000 -0.573022000 -0.176682000

C -0.771818000 -1.954579000 1.204848000

H -1.331113000 -2.653247000 1.846620000

C 0.571339000 -2.019930000 1.156725000

H 1.104005000 -2.771704000 1.763314000

N -0.965211000 -1.507249000 -2.011590000

H 0.435014000 -1.250845000 -2.033426000

H -1.371006000 -1.206700000 -2.897720000

H -1.082321000 -2.520900000 -1.972962000

C -3.692758000 0.295503000 -0.443508000

C -4.797709000 -0.422226000 0.048268000

C -6.100106000 0.054116000 -0.143591000

H -6.947011000 -0.517056000 0.246903000

C -6.314804000 1.245384000 -0.832583000

H -7.332200000 1.614507000 -0.985417000

C -5.229744000 1.964620000 -1.329208000

H -5.392773000 2.899593000 -1.872804000

C -3.924250000 1.497792000 -1.137148000

C -2.767886000 2.278183000 -1.662011000

C -2.214078000 3.305360000 -0.873053000

C -1.085112000 3.984840000 -1.340384000

H -0.649256000 4.780408000 -0.730861000

C -0.506204000 3.649269000 -2.562070000

H 0.385184000 4.178101000 -2.908892000

C -1.069725000 2.643802000 -3.343314000

H -0.624793000 2.387873000 -4.308759000

C -2.211975000 1.956650000 -2.914395000

C -4.563887000 -1.705802000 0.770241000

C -4.375393000 -1.695824000 2.165946000

C -4.121272000 -2.904018000 2.822202000

H -3.974153000 -2.901211000 3.905639000

C -4.045922000 -4.098964000 2.112332000

H -3.844462000 -5.035763000 2.637839000

C -4.219883000 -4.098344000 0.731716000

H -4.154905000 -5.035260000 0.171865000

C -4.480506000 -2.908974000 0.042531000

In 1.660334000 -0.483153000 0.061448000

C 2.025147000 0.126354000 -2.895752000

H 2.888399000 0.223834000 -3.566929000

C 1.700579000 -1.023353000 -2.267663000

H 2.362092000 -1.881011000 -2.461066000

N -0.039064000 0.878472000 -0.178689000

H 1.413134000 1.031850000 -2.782722000

H -0.096892000 1.533583000 0.602308000

H -0.028046000 1.458287000 -1.018110000

C 3.557473000 0.342803000 0.762600000

C 3.530213000 1.334279000 1.760840000

C 4.719681000 1.818998000 2.317883000

H 4.676207000 2.589900000 3.092471000

C 5.947425000 1.315368000 1.896329000

H 6.874880000 1.689588000 2.337052000

C 5.988323000 0.326718000 0.916547000

H 6.948322000 -0.080023000 0.586362000

C 4.804585000 -0.161503000 0.349799000

C 4.873282000 -1.219065000 -0.697523000

C 5.193110000 -0.862761000 -2.021554000

C 5.208592000 -1.856329000 -3.004863000

H 5.445750000 -1.582323000 -4.036602000

C 4.916079000 -3.179775000 -2.686024000

H 4.924067000 -3.945580000 -3.465691000

C 4.620077000 -3.527282000 -1.371330000

H 4.404275000 -4.568045000 -1.115374000

C 4.602692000 -2.559914000 -0.360052000

C 2.220205000 1.860392000 2.240509000

C 1.689969000 3.036551000 1.674801000

C 0.443943000 3.495097000 2.114085000

H 0.032159000 4.411705000 1.685540000

C -0.277839000 2.791097000 3.075449000

H -1.256249000 3.154372000 3.398956000

C 0.249732000 1.626159000 3.623499000

H -0.313535000 1.072899000 4.379416000

C 1.507141000 1.153797000 3.229226000

C -4.638425000 -2.914618000 -1.451471000

H -5.542149000 -2.372365000 -1.766658000

H -3.785470000 -2.413939000 -1.939801000

H -4.692607000 -3.941444000 -1.838903000

C -4.414090000 -0.405032000 2.931761000

H -4.304542000 -0.581626000 4.010602000

H -3.601493000 0.267542000 2.610569000

H -5.354072000 0.141032000 2.760090000

C -2.850255000 0.923788000 -3.798026000

H -3.751063000 1.333831000 -4.284029000

H -2.161583000 0.598695000 -4.590774000

H -3.181932000 0.044211000 -3.228565000

C -2.812358000 3.639518000 0.461475000

H -2.715446000 2.790795000 1.157544000

H -2.319106000 4.511143000 0.912185000

H -3.888536000 3.851228000 0.377625000

C 5.492252000 0.564825000 -2.376088000

H 4.699082000 1.237775000 -2.014427000

H 6.427970000 0.909065000 -1.908314000

H 5.589435000 0.690666000 -3.463569000

C 4.337784000 -2.956576000 1.064424000

H 5.199379000 -2.713896000 1.706312000

H 3.477643000 -2.418324000 1.488777000

H 4.138592000 -4.033955000 1.143589000

C 2.425302000 3.755943000 0.582366000

H 1.896662000 4.671736000 0.284271000

H 3.448073000 4.023929000 0.886337000

H 2.522776000 3.110495000 -0.306008000

C 2.085296000 -0.080147000 3.858911000

H 3.072775000 0.125755000 4.299616000

H 1.423569000 -0.467877000 4.645099000

H 2.231455000 -0.880430000 3.117665000

**INT4:** G = -2345.86999478

In 5.492071000 8.276537000 1.306873000

C 4.368120000 8.097990000 -0.555594000

H 3.350652000 8.508542000 -0.660792000

C 4.983369000 7.625398000 -1.655034000

H 4.480238000 7.647106000 -2.635513000

N 6.697926000 9.985346000 0.904224000

H 7.789428000 9.436792000 -0.893758000

H 6.994237000 10.511252000 1.724769000

H 6.151220000 10.642239000 0.345046000

C 4.912882000 8.156949000 3.414365000

C 3.640682000 8.642652000 3.766573000

C 3.253051000 8.733518000 5.109095000

H 2.257933000 9.112260000 5.359113000

C 4.133457000 8.351861000 6.118494000

H 3.835473000 8.433364000 7.166975000

C 5.394661000 7.861739000 5.786780000

H 6.087740000 7.553141000 6.574825000

C 5.783867000 7.756083000 4.446213000

C 7.109663000 7.168750000 4.098039000

C 7.232440000 5.764423000 4.050450000

C 8.436991000 5.201399000 3.621029000

H 8.532566000 4.113421000 3.575045000

C 9.507143000 6.010587000 3.243847000

H 10.438692000 5.558651000 2.893638000

C 9.390543000 7.394864000 3.328067000

H 10.236544000 8.031996000 3.055352000

C 8.202388000 7.992114000 3.771016000

C 2.699846000 9.062373000 2.687594000

C 1.846626000 8.107453000 2.100213000

C 0.983502000 8.510016000 1.076646000

H 0.317657000 7.772713000 0.619813000

C 0.971325000 9.829048000 0.631886000

H 0.292338000 10.130114000 -0.169896000

C 1.831115000 10.761834000 1.203658000

H 1.829125000 11.796423000 0.849806000

C 2.703675000 10.396176000 2.234843000

In 6.868694000 6.609702000 -1.400220000

C 9.269976000 7.938059000 -0.927211000

H 9.962227000 7.306889000 -1.489328000

C 8.425676000 8.797138000 -1.528861000

H 8.409513000 8.899480000 -2.619863000

N 6.869068000 6.579132000 0.769139000

H 9.317362000 7.885336000 0.165744000

H 6.458359000 5.706133000 1.106621000

H 7.776290000 6.641050000 1.231411000

C 7.669281000 4.977185000 -2.597128000

C 7.246038000 3.691505000 -2.211415000

C 7.606222000 2.566309000 -2.959951000

H 7.265760000 1.576287000 -2.644288000

C 8.384008000 2.711668000 -4.105875000

H 8.664263000 1.834242000 -4.693954000

C 8.789970000 3.980637000 -4.509639000

H 9.384206000 4.102535000 -5.419421000

C 8.432861000 5.115682000 -3.769582000

C 8.854224000 6.458325000 -4.260116000

C 10.184545000 6.884227000 -4.076374000

C 10.556181000 8.157076000 -4.521321000

H 11.582753000 8.498448000 -4.363223000

C 9.637356000 8.986894000 -5.157239000

H 9.940686000 9.979484000 -5.499078000

C 8.333111000 8.546872000 -5.363331000

H 7.614392000 9.189642000 -5.878576000

C 7.922834000 7.283046000 -4.924126000

C 6.379324000 3.555561000 -1.006736000

C 6.955754000 3.237226000 0.238597000

C 6.130215000 3.166691000 1.363975000

H 6.569317000 2.910335000 2.329944000

C 4.766449000 3.435066000 1.268536000

H 4.137343000 3.394107000 2.161025000

C 4.205860000 3.754094000 0.036382000

H 3.135891000 3.963110000 -0.040549000

C 4.994839000 3.800187000 -1.119258000

C 3.653902000 11.401878000 2.819319000

H 3.639553000 11.386306000 3.918906000

H 4.688902000 11.178623000 2.509407000

H 3.411871000 12.418266000 2.478729000

C 1.891401000 6.671500000 2.536852000

H 1.090864000 6.087755000 2.061461000

H 2.856449000 6.212486000 2.263889000

H 1.797194000 6.573551000 3.628683000

C 8.134543000 9.481982000 3.943256000

H 8.441091000 9.760650000 4.965488000

H 8.808818000 9.994158000 3.242481000

H 7.115892000 9.865298000 3.801282000

C 6.076465000 4.891673000 4.443363000

H 5.213029000 5.058696000 3.780474000

H 6.348036000 3.828320000 4.402390000

H 5.731510000 5.119574000 5.462503000

C 11.196240000 5.989447000 -3.417586000

H 10.780878000 5.485386000 -2.531836000

H 11.523434000 5.188797000 -4.100515000

H 12.087932000 6.556845000 -3.116346000

C 6.518781000 6.816209000 -5.183748000

H 6.506396000 5.798129000 -5.600505000

H 5.921489000 6.783660000 -4.259395000

H 6.006367000 7.489233000 -5.884503000

C 8.435696000 3.025346000 0.363698000

H 8.707192000 2.720528000 1.383405000

H 8.800618000 2.262227000 -0.339421000

H 8.981103000 3.954316000 0.128838000

C 4.363698000 4.072901000 -2.454527000

H 4.471902000 3.203095000 -3.121821000

H 3.294090000 4.295980000 -2.344408000

H 4.835710000 4.920451000 -2.972751000

**7M:** G = -2267.50979961

In 6.463053000 6.691386000 9.055399000

In 4.264184000 7.671988000 10.957293000

C 5.683716000 5.124327000 10.344314000

H 6.007141000 4.071108000 10.364645000

C 4.715664000 5.555342000 11.179585000

H 4.242307000 4.856218000 11.887787000

N 4.453849000 7.599007000 8.740528000

H 3.772814000 7.014312000 8.255024000

H 4.435013000 8.498899000 8.262013000

N 6.406033000 8.213830000 10.681688000

H 7.008582000 8.026825000 11.483863000

H 6.600756000 9.175531000 10.405091000

C 7.942377000 6.926943000 7.505405000

C 9.261639000 7.281841000 7.829487000

C 10.219630000 7.412367000 6.817264000

H 11.246094000 7.686945000 7.075978000

C 9.867399000 7.189479000 5.487107000

H 10.619598000 7.288745000 4.700351000

C 8.558913000 6.837992000 5.157854000

H 8.281955000 6.661938000 4.114713000

C 7.594852000 6.703963000 6.162857000

C 9.602557000 7.522629000 9.260867000

C 9.847289000 6.429809000 10.115153000

C 10.073360000 6.673191000 11.475029000

H 10.260811000 5.827845000 12.142466000

C 10.066274000 7.969946000 11.979068000

H 10.243671000 8.144643000 13.043179000

C 9.830695000 9.044534000 11.125808000

H 9.817306000 10.063986000 11.521159000

C 9.590755000 8.838094000 9.764592000

C 6.186326000 6.326086000 5.853202000

C 5.774736000 4.989168000 6.021115000

C 4.426860000 4.667929000 5.824829000

H 4.101682000 3.632800000 5.958771000

C 3.506107000 5.645725000 5.461175000

H 2.456012000 5.381167000 5.314284000

C 3.923455000 6.962388000 5.289297000

H 3.199106000 7.733331000 5.013370000

C 5.259708000 7.321740000 5.487896000

C 2.963810000 9.133354000 11.864491000

C 3.343838000 9.713466000 13.086345000

C 2.505851000 10.642288000 13.712563000

H 2.807656000 11.089641000 14.663674000

C 1.289788000 10.991242000 13.126610000

H 0.634493000 11.713718000 13.619815000

C 0.906588000 10.420718000 11.913746000

H -0.046989000 10.695002000 11.453985000

C 1.739542000 9.492073000 11.278707000

C 4.653699000 9.311859000 13.675288000

C 4.745939000 8.114654000 14.412735000

C 6.001853000 7.695385000 14.865458000

H 6.079918000 6.763442000 15.431562000

C 7.142215000 8.448430000 14.602978000

H 8.117303000 8.105916000 14.958229000

C 7.040161000 9.635684000 13.883405000

H 7.936965000 10.222875000 13.668688000

C 5.803372000 10.079686000 13.406507000

C 1.362743000 8.871810000 9.976493000

C 0.781394000 7.589336000 9.956761000

C 0.516318000 6.986843000 8.721548000

H 0.069355000 5.989309000 8.701048000

C 0.811797000 7.642086000 7.530026000

H 0.600694000 7.158817000 6.572919000

C 1.381292000 8.911920000 7.559323000

H 1.622277000 9.424820000 6.624103000

C 1.669881000 9.538935000 8.774773000

C 3.520848000 7.294501000 14.700229000

H 3.082631000 6.887494000 13.774584000

H 3.757016000 6.450123000 15.362027000

H 2.734597000 7.902317000 15.173058000

C 5.708194000 11.329420000 12.580689000

H 6.698997000 11.779015000 12.426557000

H 5.267067000 11.113043000 11.594398000

H 5.056505000 12.080445000 13.053049000

C 2.343092000 10.880285000 8.799125000

H 1.760798000 11.618721000 9.370596000

H 3.326612000 10.815888000 9.293570000

H 2.492733000 11.268232000 7.781948000

C 0.459609000 6.872052000 11.237003000

H -0.061672000 5.925742000 11.038586000

H 1.371585000 6.642847000 11.811622000

H -0.171959000 7.488879000 11.894366000

C 6.759132000 3.924743000 6.414467000

H 7.146796000 4.090462000 7.432684000

H 6.294549000 2.929634000 6.388688000

H 7.634882000 3.921068000 5.747926000

C 5.689717000 8.754123000 5.359972000

H 6.162297000 9.106809000 6.290908000

H 6.439396000 8.885628000 4.564393000

H 4.833015000 9.405411000 5.137402000

C 9.854412000 5.022955000 9.587441000

H 10.262409000 4.326319000 10.332546000

H 8.836936000 4.680729000 9.335213000

H 10.448796000 4.941017000 8.665525000

C 9.272382000 9.995524000 8.863553000

H 9.978724000 10.066437000 8.022568000

H 8.272053000 9.877092000 8.415547000

H 9.294160000 10.945464000 9.415520000

# References

[57] SAINT V8.41, Bruker AXS, Madison, WI, USA, **2024**.

[58] L. Krause, R. Herbst-Irmer, G. M. Sheldrick, D. Stalke, “Comparison of silver and molybdenum microfocus X-ray sources for single-crystal structure determination” *J. Appl. Crystallogr.* **2015**, *48*, 3–10.

[59] G. M. Sheldrick, “SHELXT—Integrated space-group and crystal-structure determination” *Acta Cryst.* **2015**, *A71*, 3–8.

[60] G. M. Sheldrick, “Crystal structure refinement with SHELXL” *Acta Cryst.* **2015**, *C71*, 3–8.

[61] F. Neese, “Software Update: The ORCA Program System—Version 6.0” *Rev. Comput. Mol. Sci.* **2025**, *15*, e70019.

[62] J. P. Perdew, M. Ernzerhof, K. Burke, “Rationale for mixing exact exchange with density functional approximations” *J. Chem. Phys.* **1996**, *105*, 9982–9985.

[63] C. Adamo, V. Barone, “Toward reliable density functional methods without adjustable parameters: The PBE0 model” *J. Chem. Phys.* **1999**, *110*, 6158–6170.

[64] S. Grimme, J. Antony, S. Ehrlich, H. Krieg, H. “A consistent and accurate ab initio parametrization of density functional dispersion correction (DFT-D) for the 94 elements H–Pu” *J. Chem. Phys.* **2010**, *132*, 154104.

[65] S. Grimme, S. Ehrlich, L. Goerigk, “Effect of the damping function in dispersion corrected density functional theory” *J. Comput. Chem.* **2011**, *32*, 1456–1465.

[66] F. Neese, “An improvement of the resolution of the identity approximation for the formation of the Coulomb matrix” *J. Comp. Chem.* **2003**, *24*, 1740–1747.

[67] F. Weigend, R. Alhrichs, “Balanced basis sets of split valence, triple zeta valence and quadruple zeta valence quality for H to Rn: Design and assessment of accuracy” *Phys. Chem. Chem. Phys.* **2005**, *7*, 3297–3305.

[68] V. Barone, M. Cossi, “Quantum Calculation of Molecular Energies and Energy Gradients in Solution by a Conductor Solvent Model” *J. Phys. Chem. A* **1998**, *102*, 1995–2001.

[69] V. Asgeirsson, B. Orri Birgisson, R. Björnsson, U. Becker, F. Neese, C. Riplinger, H. Jónsson, “Nudged Elastic Band Method for Molecular Reactions Using Energy-Weighted Springs Combined with Eigenvector Following” *J. Chem. Theory Comput.* **2021**, *17*, 4929–4945.
